# A General and Scalable Synthesis of Polysubstituted Indoles

**DOI:** 10.3390/molecules25235595

**Published:** 2020-11-28

**Authors:** David Tejedor, Raquel Diana-Rivero, Fernando García-Tellado

**Affiliations:** 1 Instituto de Productos Naturales y Agrobiología, Consejo Superior de Investigaciones Científicas, Astrofísico Francisco Sánchez 3, 38206 La Laguna, Spain; raquel.diana.rivero@gmail.com; 2 Doctoral and Postgraduate School, Universidad de La Laguna, Apartado Postal 456, 38200 La Laguna, Spain

**Keywords:** indol, indole-3-carboxylates, indole-3-carboxyamides, 3-aroylindoles, 3-acylindoles, *N*-oxyenamines, [3,3]-sigmatropic rearrangement, annulation, DABCO, organocatalysis

## Abstract

A consecutive 2-step synthesis of *N*-unprotected polysubstituted indoles bearing an electron-withdrawing group at the C-3 position from readily available nitroarenes is reported. The protocol is based on the [3,3]-sigmatropic rearrangement of *N*-oxyenamines generated by the DABCO-catalyzed reaction of *N*-arylhydroxylamines and conjugated terminal alkynes, and delivers indoles endowed with a wide array of substitution patterns and topologies.

## 1. Introduction

**Scheme 1:**
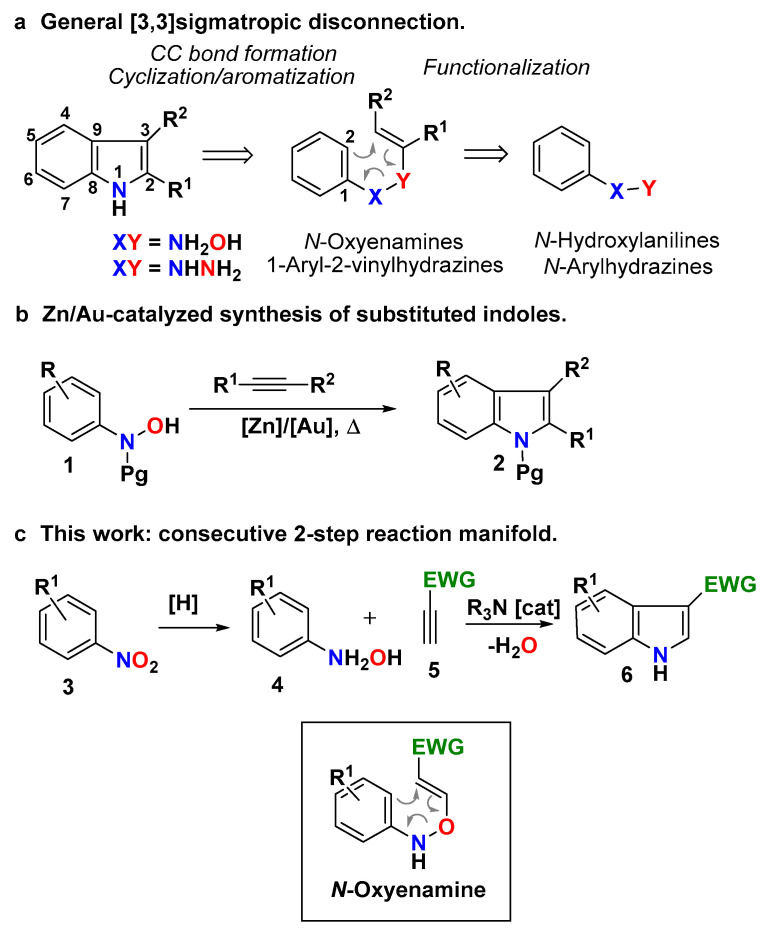
Strategy for the synthesis of indoles 6 with electron-withdrawing group at the C-3 position (3-EWG indoles). (**a**) General [3,3] sigmatropic disconnection. (**b**) Zn/Au- catalyzed synthesis of indoles. (**c**) This work: consecutive two-step reaction manifold.

The indole moiety occupies a privileged place in the realm of heterocyclic chemistry [1]. It constitutes the core structure of many natural products and synthetic bioactive compounds exhibiting a wide array of biological activities [2,3,4,5,6], and has also found use as structural parts of material science devices [7,8,9] and chiral catalysts [10,11,12]. Recent studies have shown that the substitution at the C-3 position with electron-withdrawing groups (3-EWG-indoles) not only endows these structures with important pharmaceutical/therapeutic activities [13,14,15,16,17,18,19,20,21], but also it converts them into versatile synthetic molecular platforms in heterocyclic chemistry, such as, in dearomatization-based synthetic disconnections [22]. The catalytic asymmetric dearomatization of 3-nitroindoles constitutes an iconic example of this synthetic potential [23,24,25,26,27,28,29,30,31,32,33,34]. The most traditional synthetic approach to these functionalized indoles relies on the Friedel–Crafts acylation of preformed indoles [35,36,37]. This transformation suffers important drawbacks such as mandatory nitrogen protection, regioselectivity control, stoichiometric use of metal salts and Lewis acid promoters, and strict exclusion of moisture. Although modern alternatives using metal-catalysis [38,39,40,41,42] or photocatalysis [43,44,45,46] have overcome some of these difficulties, the implementation of real-world strategies to prepare these heterocycles from simple and widely accessible starting materials remains a synthetic challenge in medicinal and synthetic chemistry. In spite of the many advances achieved in the synthesis of indoles during the last 100 years [47], the de novo synthesis of 3-EWG-indoles remains largely unexplored. A large number of strategies for the synthesis of indoles use alkynes and a convenient nitrogen source as substrates [48] and the annelation of the five-membered ring to an existing functionalized benzene ring as the main synthetic disconnection [49]. The inherent alkyne reactivity and the broad access to nitrogen sources constitute practical advantages for these strategies. Among them, the [3,3]-sigmatropic rearrangement of *N*-oxyenamines [50] or 1-aryl-2-vinylhydrazines [51,52] have been objects of profuse research (Scheme 1a). Prototypical examples of these strategies constitute the Bartoli [53,54] (*N*-oxyenamines) and the Fischer [55] (1-aryl-2-vinylhydrazines) syntheses of indoles. Although both reactions have been largely used in the preparation of indoles [47], they present important drawbacks for general preparative synthetic use. Whereas those based on the Fischer disconnection suffer the limitations associated with the hydrazine synthesis and drastic reaction conditions, the Bartoli method, although synthetically powerful, requires the use of *ortho*-substituted nitroarenes and functional tolerance to Grignard reagents. Recently, an elegant example of the use of the *N*-oxyenamine disconnection to gain access to polysusbtituted indoles has been reported by Zhang and co-workers (Scheme 1b) [56]. The methodology uses the Zn/Au-catalyzed reaction of N-protected *N*-arylhydroxamic acids (or *N*-aryl-*N*-hydroxycarbamates) **1** and alkynes to construct the indole core with a diverse pattern of substitution at both rings. Although the methodology was effective for the synthesis of alkyl indole-3-carboxylates (**2**, the unique reported example: Pg = Ac; R^1^ = CH_2_CH_2_Ph, R^2^ = CO_2_Me), its extension to other 3-EWG-indoles remains to be assessed [57].

We envisioned that a simpler and general methodology using the *N*-oxyenamine disconnection could be arranged on the basis of the well-established addition of nucleophiles onto terminal conjugated alkynes catalyzed by tertiary amines (Scheme 1c) [58,59,60,61]. When searching for precedents for this disconnection, we found a reduced number of reported examples, all of them circumscribed to the synthesis of alkyl indole-3-carboxyates [62,63,64,65,66], and with important drawbacks for a practical and general application. They mostly use *N*-arylhydroxylamine salts or *N*-protected *N*-arylhydroxylamines to force the *O*-addition reaction, and require large excesses of alkyne and long reaction times to deliver the indole-3-carboxylates in low to moderate yields. Therefore, a general and practical protocol for accessing NH-free 3-EWG-indoles based on this disconnection remains to be established. In terms of sustainability, such protocol should be direct, operationally simple and scalable, avoiding non-productive N-protection/N-deprotection steps. We report herein our results on the implementation of a strategy fulfilling these requirements and overcoming the practical shortcomings found in previous reports. The strategy delivers NH-free multisubstituted 3-EWG-indoles through a consecutive 2-step reaction manifold, and it uses nitroarenes and activated terminal alkynes as building blocks (Scheme 1c). The reaction manifold takes advantage of the *O*-reactivity of *N*-arylhydroxylamines toward terminal conjugated alkynes and of the rich arsenal of commercially available functionalized nitroarenes. Whereas the first one avoids unnecessary *N*-protection/*N*-deprotection steps (step-economy, reagent-economy), the second one offers an almost countless catalogue of functional diversity on the benzene ring (general outcome). These properties make this reaction manifold a convenient, instrumentally simple, robust and reliable synthetic tool for the exploration/annotation of the chemical space of 3-EWG-indoles. 

## 2. Results and Discussion

### 2.1. Initial Experiments and Optimization of the Reaction Conditions for a Consecutive 2-Step Protocol

**Scheme 2:**
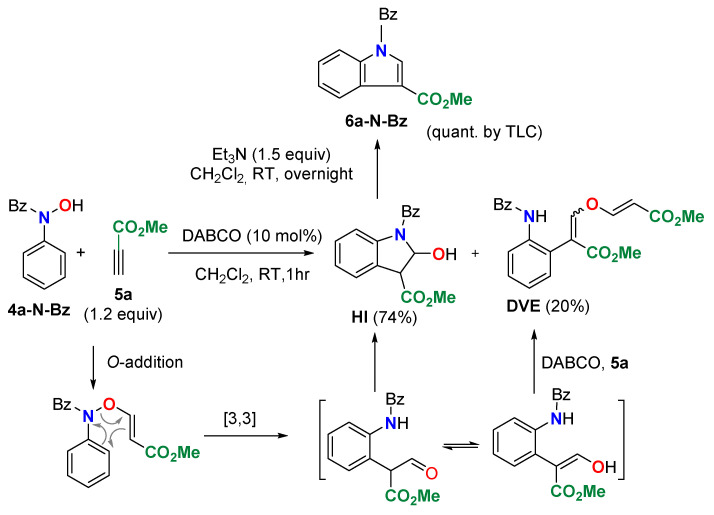
DABCO-catalyzed reaction of *N*-Benzoyl-*N*-phenylhydroxylamine (**4a**-**N**-**Bz**) and methyl propiolate (**5a**).

The initial studies began using commercially available *N*-benzoyl-*N*-phenylhydroxylamine (**4a**-**N-Bz**) as the nitrogen species needed for the synthesis of indoles and the standard conditions reported by our group for the DABCO-catalyzed synthesis of propargyl vinyl ethers [59] (Scheme 2). Although the addition of the hydroxylamine to methyl propiolate (**5a**) took place in the presence of DABCO, and this was followed by the desired cyclization, the hydroxyindoline intermediate **HI** was isolated instead of the expected indole **6a-N-Bz**. Furthermore, a side-product incorporating a second unit of methyl propiolate was also obtained (**DVE**). It forms from the DABCO-catalyzed addition of methyl propiolate (**5a**) on the enol ether generated on the [3,3]sigmatropic rearrangement of the intermediate *N*-oxyenamine. The overnight reaction of intermediate **HI** with Et_3_N afforded the indole **6a-N-Bz** in quantitative yield. 

At this time, we decided to question the need for the protecting group on the nitrogen atom. Indeed, when starting from *N*-phenylhydroxylamine (**4a**) and methyl propiolate (**5a**) (1.2 equiv) as the reactants, the reaction proceeded smoothly to the desired unprotected indole **6a** (Table 1, entry 1). The observed outcome was different to that reported by Hwu et al. [64] in which *N*-vinyl protected indoles were obtained when *N*-phenylhydroxylamine (**4a**) reacted with an excess of methyl propiolate (**5a**) in the presence of a catalytic amount of DMAP. Strikingly, the report by Hwu et al. claims that DABCO and Et_3_N did not give significant amounts of the desired indoles under their reaction conditions.

Once the feasibility of the reaction using free *N*-phenylhydroxylamine (**4a**) was established, we next undertook the optimization of the reaction (Table 1). Under all assayed conditions, the formation of the desired indole **6a** was accompanied by the formation of very small amounts of side-product **DVE**. Based on these results, and taking into consideration the simplicity of the experimental procedure, we chose the reaction conditions depicted in entry 4 as the standardized conditions for the formation of the desired indoles **6** from the appropriate *N*-arylhydroxylamines **4**. 

**Scheme 3:**
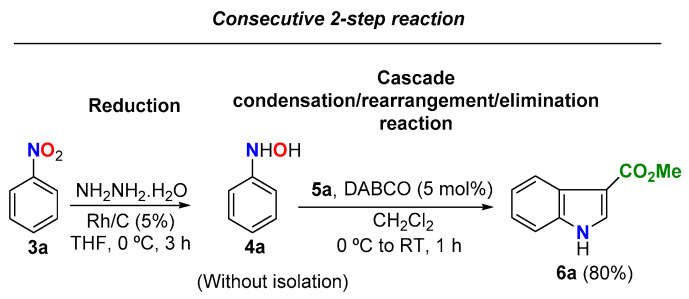
Coupling reduction and cascade condensation/rearrangement/elimination reactions in a consecutive 2-step protocol.

Once this reaction was optimized, we coupled this reaction with the reduction of the nitrobenzene (**3a**) to set up a consecutive 2-step reaction manifold (Scheme 3). Thus, reduction of nitrobenzene (**3a**) with hydrazine monohydrate and Rh on activated charcoal (5%) in THF gave *N*-phenylhydroxylamine (**4a**), which, after filtration of the reaction mixture and concentration, was directly submitted to the next reaction step to deliver indole **6a** in 80% overall yield. Under these conditions, the overall yield of indole **6a** was comparable to that obtained directly from **4a** (80% vs 82%).

### 2.2. Scope of the Consecutive 2-Step Protocol

**Scheme 4:**
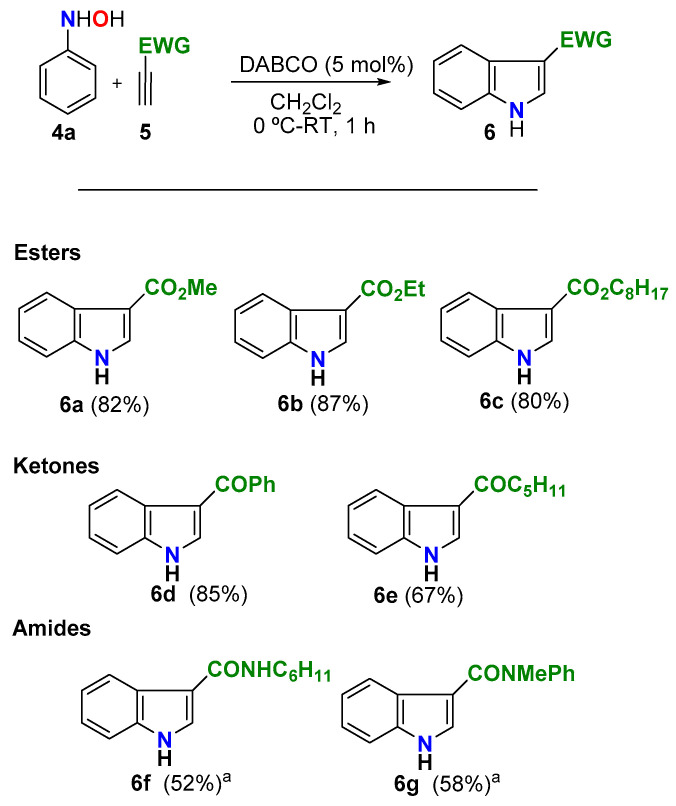
Alkyne scope of the protocol. Reaction conditions: *N*-phenylhydroxylamine (**4a**) (4.0 mmol), alkyne **5** (4.2 mmol), DABCO (5 mol%), CH_2_Cl_2_ (20 mL), 0 °C to RT, 1 h. ^a^ Overnight.

With a convenient protocol in hand, we next explored its scope with regard to the alkyne using *N*-phenylhydroxylamine (**4a**) as the nitrogen component (Scheme 4). The reaction was well suited to prepare 3-EWG-indoles incorporating carboxylates (**6a**–**c**), aroyl (acyl) groups (**6d,e**), or carboxamides (**6f**,**g**). In the case of *N*,*N*-disubstituted propionamides **5f** and **5g**, the poorest Michael acceptors of the series, the reaction needed more time (overnight) to deliver the corresponding *N*,*N*-disubstituted indole-3-carboxamides **6f** and **6g** in synthetically relevant 52% and 58% yields respectively. The efficiency and simplicity of this transformation are emphasized when compared with the current multistep methodologies to prepare these indole-3-carboxamides [67].

**Scheme 5:**
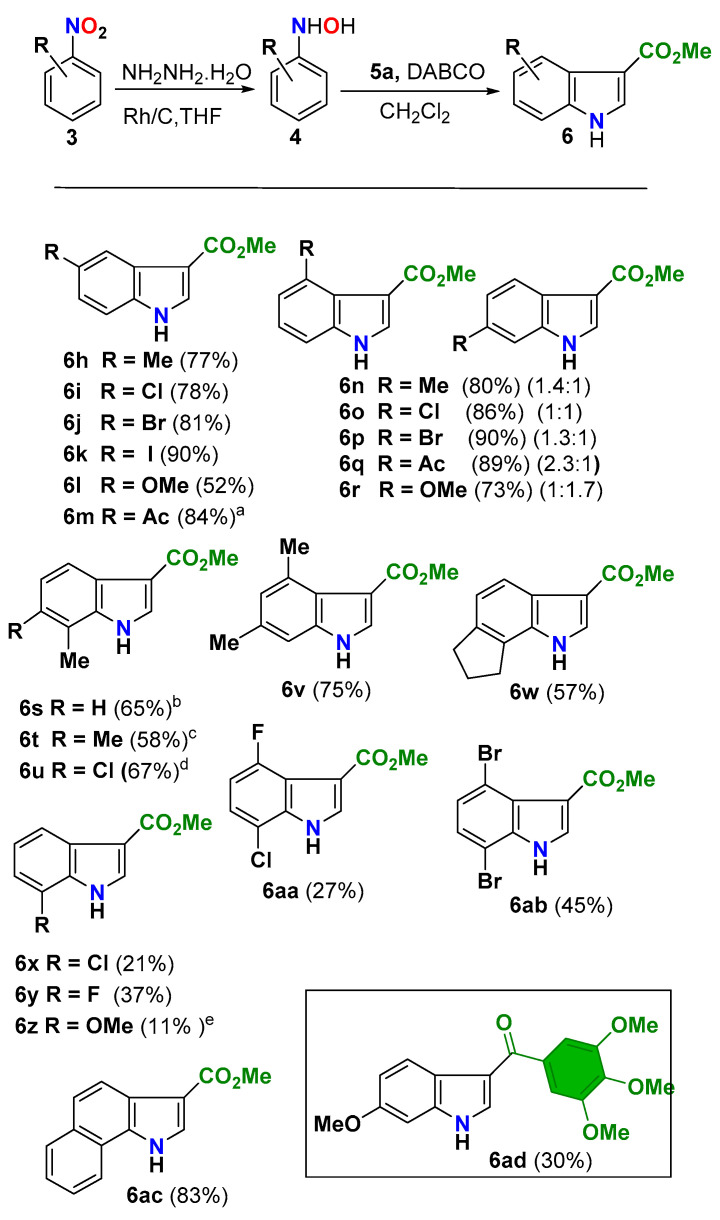
Nitroarene scope of the protocol. Reaction conditions: 1) nitroarene **3** (4 mmol), NH_2_NH_2_.H_2_O (4.4 mmol), Rh/C (5%) (20 mg), THF (20 mL), 0 °C to RT, 3 h, filtration on celite plug and concentration; 2) methyl propiolate (**5a**) (4.2 mmol), DABCO (5 mol%), CH_2_Cl_2_ (20 mL), 0 °C to RT, 1 h. ^a^50 mmol scale. ^b^**10s** (21%). ^c^**10t** (34%). ^d^**10u** (27%). ^e^**10z** (22%) (See Scheme 6 for structures).

The scope of the consecutive 2-step manifold with regard to the substitution at the benzene ring was explored using methyl propiolate (**5a**) as the alkyne component and differently substituted nitroarenes **3** (Scheme 5). The manifold tolerated a diverse substitution pattern on the benzene ring to deliver the corresponding methyl indole-3-carboxylates **6** with overall good efficiency and broad functional/structural diversity. A convenient grade of topological diversity could be generated, spanning from the simple monosubstituted derivatives (e.g., **6h**–**r**, 52–90% yield) to more complex polycyclic (**6w**, 57%) or fused aromatic systems (**6ac**, 83%). *Meta*-substituted *N*-arylhydroxylamines afforded separable mixtures of the corresponding C-4/C-6 regioisomers in excellent yields (80–90%) and with a slight substituent- dependent regioselectivity (Scheme 5, compounds **6n**–**r**). A balance between steric and electronic effects of the substituent seems to steer the outcome of the [3,3]-sigmatropic rearrangement, in line with the reported results in the Fischer synthesis of indoles endowed with this substitution pattern [52,65,68]. Thus, electron-donating substituents (R = OMe) unbalanced the chemical outcome towards the less crowed isomer (**6r**, 1.7:1 / C-6: C-4 ratio) whereas electron-withdrawing groups did it in the opposite direction (**6o**–**q**; from 1:1 to 2.3:1 / C-4:C-6 ratio). It is remarkable that the [3,3]-sigmatropic rearrangement of benzyl ketene acetals bearing a *meta*-OMe substituent on the aromatic ring preponderantly delivers the C-4 isomer. Theoretical studies pointed to a stabilizing interaction between the lone pair of the substituent and the LUMO orbital of the transition state as being responsible for the observed regioselectivity [69]. The observed inverted regioselectivity in our reaction seems to rule out the existence of this kind of stabilizing interactions. 

**Scheme 6:**
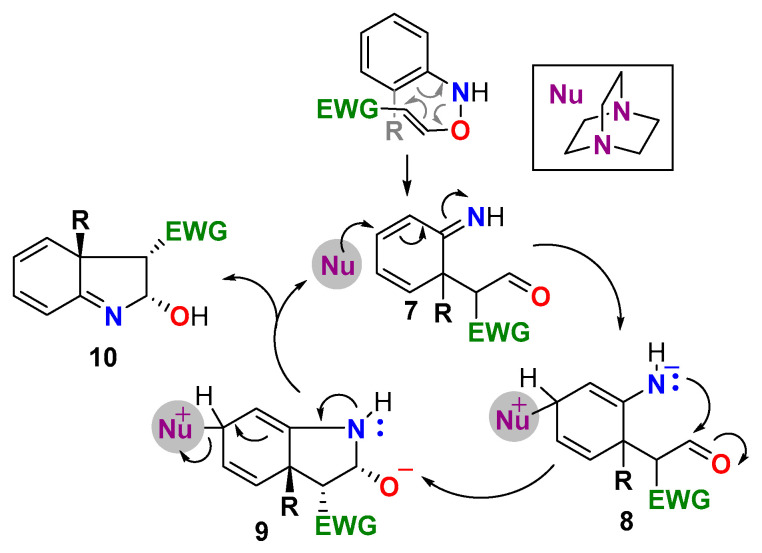
Alternative [3,3]-sigmatropic rearrangement outcome of *ortho*-substituted *N*-oxyenamines.

As expected, substitution at the *ortho*-position posed a regioselectivity problem in the [3,3]-sigmatropic rearrangement, since the C-C bond formation at the most substituted *ortho*-position becomes a competitive pathway (Scheme 6). Indeed, in some cases, we were able to isolate the product **10** obtained from this pathway. The effect of this mechanistic divergence was mainly reflected in a diminished efficiency in the reactions of nitroarenes bearing a good leaving group at that position (**6x**–**z**, **6aa**–**ab**). In the case of simple alkyl substituents (**6s**–**u**) or fused bicycles (**6w**), the manifold mostly afforded the expected C-7 substituted indole derivatives in moderate to good yields. The versatility of this methodology is highlighted when compared with Zhangs’ protocol [56] (Scheme 1B), which is sensitive to steric impediments and shows a reduced tolerance to alkyl substitution at this position [70]. However, the easy access to *ortho*-substituted nitroarenes together with the simplicity of the 2-step reaction manifold compensate for these deficiencies and make this methodology a viable synthetic alternative to prepare C-7 substituted 3-EWG indoles. 

The reaction manifold was well suited to scale up. As a proof of concept, we carried out the synthesis of methyl 6-acetylindole-3-carboxylate (**6m**) at 50 mmol scale from 4-acetylnitrobenzene (**3m**) and methyl propiolate (**5a**). The scaled manifold afforded indole **6m** in an excellent 84% yield (9.08 g) (Scheme 5).

Finally, the anticancer activity of some 3-aroyl indoles [17,19] led us to evaluate our methodology as a novel and practical access to the chemical/therapeutic space of these derivatives. Among the huge number of possible 3-aroylindoles, we chose (6-methoxy-1H-indole-3-yl) (3,4,5-trimethoxyphenyl) methanone, the so-called BPRL0T075 (**6ad**, box in Scheme 5), an extremely potent cytotoxic compound with good pharmacological properties [21]. The manifold delivered it in a 30% yield along with its C-4 isomer (25%).

## 3. Materials and Methods 

### 3.1. General Information

^1^H-NMR and ^13^C-NMR spectra (see supplementary materials) of DMSO-d6 or CDCl_3_ solutions were recorded either at 400 and 100 MHz or at 500 and 125 MHz (Bruker Ac 200 and AMX2-500 respectively). Mass spectra (low resolution) (EI/CI) were obtained with a Hewlett-Packard 5995 gas chromatograph/mass spectrometer. High-resolution mass spectra were recorded with a Micromass Autospec mass spectrometer. Analytical thin-layer chromatography plates used were E. Merck Brinkman UV-active silica gel (Kieselgel 60 F254) on aluminum. Flash column chromatography was carried out with E. Merck silica gel 60 (particle size less than 0.020 mm) using appropriate mixtures of ethyl acetate and hexanes, or ethyl acetate and dichloromethane as eluents. All reactions were performed in oven-dried glassware. All materials were obtained from commercial suppliers and used as received. *N*-phenylhydroxylamine (4a) was prepared on a multigram scale following a standard experimental procedure [71]. 

### 3.2. General Procedure for the Synthesis of Indoles ***6*** Directly from N-phenylhydroxylamine (***4a***). Preparation of Methyl Indol-3-carboxylate (***6a***)

To a solution of *N*-phenylhydroxylamine (**4a**) (436 mg, 4.0 mmol) in dichloromethane (20 mL) was added the activated alkyne **5** (4.2 mmol) and the resulting solution was cooled to 0 °C using an ice bath. DABCO (22 mg, 0.2 mmol) was added all at once and the reaction mixture was stirred for 1 h. The solvent was removed under reduced pressure, and the residue was purified by flash column chromatography (silica gel; n-hexane/ethyl acetate: 70/30 v/v) to give indole **6a** (565.2 mg, 80%). Off-white solid: ^1^H-NMR (CDCl_3_, 400 MHz): δ = 3.93 (s, 3H), 7.25–7.28 (m, 2H), 7.40–7.42 (m, 1H), 7.91 (d, 1H, *J* = 3.0 Hz), 8.18-8.20 (m, 1H), 8.82 (bs, 1H). ^13^C-NMR (CDCl_3_, 100 MHz): δ = 51.1, 108.7, 111.5, 121.5, 122.0, 123.2, 125.6, 131.1, 136.1, 165.8 ppm. The values are in accordance with the literature [72].

### 3.3. General Procedure for the Consecutive 2-Step Synthesis of Indoles ***6*** from Nitroarenes ***3***. Preparation of Methyl Indol-3-carboxylate (***6a***) 

Under a nitrogen atmosphere, the nitrobenzene (**3a**) (4 mmol) was dissolved in dry tetrahydrofuran (20 mL) and Rh/C (5%) (20 mg) was added to the solution. The resulting mixture was cooled to 0 °C, hydrazine monohydrate (250 mg, 5 mmol) was then added dropwise and the reaction was stirred at 0 °C for 3 h. The reaction mixture was filtered through a short pad of celite (aided with the use of dichloromethane) and the solvents evaporated under reduced pressure. The resulting crude product was dissolved in dichloromethane (20 mL) and methyl propiolate (**5a**) (344 mg, 4.2 mmol) was added. The resulting solution was cooled to 0 °C using an ice bath and DABCO (22 mg, 0.2 mmol) was added at once. The reaction mixture was stirred for 1 h and the solvent was removed under reduced pressure. The crude residue was purified by flash column chromatography (silica gel; *n*-hexane/ethyl acetate: 70/30 *v*/*v*) to give indole **6a** [72].

### 3.4. Characterization Data for Indoles ***6*** and 3,3a-Dihydro-2H-Indoles ***10***

*Ethyl 1H-indole-3-carboxylate* (**6b**) (330.2 mg, 87%). Off-white solid: ^1^H-NMR (DMSO-d_6_, 500 MHz): δ = 1.33 (t, 3H, *J* = 7.1 Hz), 4.28 (q, 2H, *J* = 7.1 Hz), 7.16–7.21 (m, 2H), 7.47 (dd, 1H, *J* = 6.5 and 1.6 Hz), 8.00 (dd, 1H, *J* = 6.8 and 2.0 Hz), 8.06 (d, 1H, *J* = 2.3 Hz), 11.90 (bs, 1H). ^13^C-NMR (DMSO-d_6_, 125 MHz): δ = 14.5, 59.0, 106.6, 112.3, 120.5, 121.2, 122.3, 125.6, 132.4, 136.4, 164.4 ppm. The values are in accordance with the literature [73].

*Octyl 1H-indole-3-carboxylate* (**6c**) (217.5 mg, 80%). Off-white solid: ^1^H-NMR (CDCl_3_, 400 MHz): δ = 0.88 (t, 3H, *J* = 6.8 Hz), 1.28–1.39 (m, 8H), 1.43–1.47 (m, 2H), 1.76–1.83 (m, 2H), 4.34 (t, 2H, *J* = 6.6 Hz), 7.24–7.29 (m, 2H), 7.39–7.42 (m, 1Hz), 7.91 (d, 1H, *J* = 2.1 Hz), 8.16–8.20 (m, 1H), 8.80 (bs, 1H). ^13^C-NMR (CDCl_3_, 100 MHz): δ = 14.0, 22.6, 26.2, 29.0, 29.2, 29.3, 31.8, 64.1, 109.2, 111.5, 121.6, 122.0, 123.1, 125.8, 131.0, 136.2, 165.5 ppm. HRMS (ESI^-^) m/z [M-H]^−^ calculated for C_17_H_22_NO_2_ 272.1651, found 272.1650.

*(1H-Indol-3-yl)(phenyl)methanone* (**6d**) (351.4 mg, 83%). Off-white solid: ^1^H-NMR (DMSO-d_6_, 400 MHz): δ = 7.22–7.28 (m, 2H), 7.52–7.62 (m, 4H), 7.78–7.80 (m, 2H), 7.93 (s, 1H), 8.26 (dd, 1H, *J* = 6.7 and 1.7 Hz), 12.10 (bs, 1H). ^13^C-NMR (DMSO-d_6_, 100 MHz): δ = 112.2, 115.0, 121.4, 121.9, 123.1, 126.2, 128.3, 128.4, 131.0, 136.7, 140.5, 190.0 ppm. The values are in accordance with the literature [74].

*1-(1H-Indol-3-yl)hexan-1-one* (**6e**) (144.5 mg, 67%). Off-white solid: ^1^H-NMR (DMSO-d_6_, 400 MHz): δ = 0.87 (t, 3H, *J* = 6.8 Hz), 1.29–1.34 (m, 4H), 1.60–1.68 (m, 2H), 2.82 (t, 2H, *J* = 7.4 Hz), 7.14–7.22 (m, 2H), 7.45–7.47 (m, 1H), 8.20 (dd, 1H, *J* = 6.9 and 1.5 Hz), 8.30 (d, 1H, *J* = 3.0 Hz), 11.88 (bs, 1H). ^13^C-NMR (DMSO-d_6_, 100 MHz): δ = 13.9, 22.0, 24.6, 31.1, 38.7, 112.0, 116.4, 121.4, 121.5, 122.6, 125.4, 133.6, 136.6, 195.5 ppm. The values are in accordance with the literature [75].

*N-cyclohexyl-1H-indole-3-carboxamide* (**6f**) (127.1 mg, 52%). The reaction time was increased to overnight. Off-white solid: ^1^H-NMR (DMSO-d_6_, 400 MHz): δ = 1.12–1.36 (m, 5H), 1.60–1.63 (m, 1H), 1.70–1.77 (m, 2H), 1.82–1.88 (m, 2H), 3.86–4.08 (m, 1H), 7.06–7.14 (m, 2H), 7.40 (d, 1H, *J* = 8.0 Hz), 7.60 (d, 1H, *J* = 6.1 Hz), 8.05 (s, 1H), 8.14 (d, 1H, *J* = 7.8 Hz), 11.5 (bs, 1H). ^13^C-NMR (DMSO-d_6_, 100 MHz): δ = 25.0 (2C), 25.4, 32.9 (2C), 47.4, 110.8, 111.6, 120.1, 121.1, 121.7, 126.3, 127.5, 136.1, 163.7 ppm. HRMS (ESI^+^) m/z [M + Na]^+^ calculated for C_15_H_18_N_2_ONa 265.1317, found 265.1314.The product had been previously described in the literature but in CDCl_3_ [76].

*N-Methyl-N-phenyl-1H-indole-3-carboxamide* (**6g**) (290.4 mg, 58%). The reaction time was increased to overnight. Off-white solid: ^1^H-NMR (DMSO-d_6_, 500 MHz): δ = 3.36 (s, 3H), 6.36 (d, 1H, *J* = 3.0 Hz), 7.07 (dd, 1H, *J* = 7.4 and 1.2 Hz), 7.11 (dd, 1H, *J* = 7.5 and 1.5 Hz), 7.28–7.32 (m, 4H), 7.37–7.40 (m, 2H), 8.04 (d, 1H, *J* = 8.0 Hz), 11.28 (bs, 1H). ^13^C-NMR (DMSO-d_6_, 125 MHz): δ = 37.9, 109.6, 111.5, 120.3, 121.3, 121.9, 126.8, 127.2, 127.5 (2C), 128.9, 129.4 (2C), 135.1, 145.5, 165.1 ppm. The values are in accordance with the literature [77].

*Methyl 5-methyl-1H-indole-3-carboxylate* (**6h**) (533.5 mg, 77%). Off-white solid: ^1^H-NMR (CDCl_3_, 400 MHz): δ = 2.48 (s, 3H), 3.92 (s, 3H), 7.08 (dd, 1H, *J* = 8.3 and 1.2 Hz), 7.29 (d, 1H, *J* = 8.3 Hz), 7.86 (d, 1H, *J* = 3.0 Hz), 7.98 (d, 1H, *J* = 1.2 Hz), 8.72 (bs, 1H). ^13^C-NMR (CDCl_3_, 100 MHz): δ = 21.5, 51.0, 108.2, 111.1, 121.1, 124.8, 126.0, 131.1, 131.6, 134.4, 165.8 ppm. The values are in accordance with the literature [78].

*Methyl 5-chloro-1H-indole-3-carboxylate* (**6i**) (309.3 mg, 78%). Off-white solid: ^1^H-NMR (DMSO-d_6_, 400 MHz): δ = 4.36 (s, 3H), 7.74 (d, 1H, *J* = 8.6 Hz), 8.05 (d, 1H, *J* = 8.6 Hz), 8.55 (s, 1H), 8.69 (s, 1H), 12.65 (s, 1H). ^13^C-NMR (DMSO-d_6_, 100 MHz): δ = 51.6, 107.2, 114.8, 120.6, 123.4, 127.1, 127.8, 134.7, 135.9, 165.4. The values are in accordance with the literature [73].

*Methyl 5-bromo-1H-indole-3-carboxylate* (**6j**) (820.0 mg, 81%). Off-white solid: ^1^H-NMR (CDCl_3_, 400 MHz): δ = 3.90 (s, 3H), 7.28 (d, 1H, *J* = 8.6 Hz), 7.36 (dd, 1H, *J* = 8.6 and 1.8 Hz), 7.91 (d, 1H, *J* = 2.9 Hz), 8.32 (d, 1H, *J* = 1.8 Hz), 8.61 (bs, 1H). ^13^C-NMR (CDCl_3_, 100 MHz): δ = 51.2, 108.8, 112.9, 115.7, 124.3, 126.3, 127.4, 131.7, 134.7, 165.1 ppm. The values are in accordance with the literature [79]. 

*Methyl 5-iodo-1H-indole-3-carboxylate* (**6k**) (542.9 mg, 90%). Off-white solid: ^1^H-NMR (DMSO-d_6_, 400 MHz): δ = 3.81 (s, 3H), 7.34 (d, 1H, *J* = 8.5 Hz), 7.48 (dd, 1H, *J* = 8.5 and 1.5 Hz), 8.09 (d, 1H, *J* = 2.9 Hz), 8.33 (d, 1H, *J* = 1.3 Hz), 12.09 (bs, 1H). ^13^C-NMR (DMSO-d_6_, 100 MHz): δ = 50.8, 85.7, 105.7, 114.8, 128.1, 128.7, 130.4, 133.2, 135.5, 164.4. ppm. The values are in accordance with the literature [80].

*Methyl 5-methoxy-1H-indole-3-carboxylate* (**6l**). (214.2 mg, 52%). Off-white solid: ^1^H-NMR (CDCl_3_, 400 MHz): δ = 3.87 (s, 3H), 3.91 (s, 3H), 6.90 (dd, 1H, *J* = 8.8 and 2.5 Hz), 7.28 (d, 1H, *J* = 8.8 Hz), 7.66 (d, 1H, *J* = 2.5 Hz), 7.85 (d, 1H, *J* = 3.0 Hz), 8.74 (bs, 1H). ^13^C-NMR (CDCl_3_, 100 MHz): δ = 51.0, 55.8, 102.8, 108.3, 112.3, 113.7, 126.7, 131.0, 131.2, 155.9, 165.8 ppm. The values are in accordance with the literature [79].

*Methyl 5-acetyl-1H-indole-3-carboxylate* (**6m**). (9.083 g, 84%). Off-white solid: ^1^H-NMR (DMSO-d_6_, 400 MHz): δ = 2.63 (s, 3H), 3.84 (s, 3H), 7.56 (d, 1H, *J* = 8.6 Hz), 7.84 (dd, 1H, *J* = 8.6 and 1.5 Hz), 8.22 (d, 1H, *J* = 3.0 Hz), 8.65 (d, 1H, *J* = 1.5 Hz), 12.25 (bs, 1H). ^13^C-NMR (DMSO-d_6,_ 100 MHz): δ = 26.7, 50.8, 107.7, 112.3, 122.0, 122.5, 125.2, 130.8, 134.2, 138.9, 164.4, 197.4 ppm. HRMS (ESI^+^) m/z [M + Na]^+^ calculated for C_12_H_11_NO_3_Na 240.0637, found 240.0634.

*Methyl 4-methyl-1H-indole-3-carboxylate* (**6n**; C-4 isomer) (348.7 mg, 46%). Off-white solid: ^1^H-NMR (CDCl_3_, 400 MHz): δ = 2.87 (s, 3H), 3.87 (s, 3H), 7.03 (d, 1H, *J* = 7.1 Hz), 7.15 (t, 1H, *J* = 7.6 Hz), 7.22 (d, 1H, *J* = 8.0 Hz), 7.89 (d, 1H, *J* = 3.0 Hz), 8.76 (bs, 1H). ^13^C-NMR (CDCl_3_, 100 MHz): δ = 22.3, 51.1, 109.2, 109.5, 123.4, 124.0, 124.4, 132.1, 132.4, 136.9, 165.4 ppm. The values are in accordance with the literature [81].

*Methyl 6-methyl-1H-indole-3-carboxylate* (**6n**; C-6 isomer) (257.7 mg, 34%). Off-white solid: ^1^H-NMR (CDCl_3_, 400 MHz): δ = 2.45 (s, 3H), 3.91 (s, 3H), 7.10 (d, 1H, *J* = 8.0 Hz), 7.18 (s, 1H), 7.83 (d, 1H, *J* = 3.0 Hz), 8.04 (d, 1H, *J* = 8.0 Hz), 8.70 (bs, 1H). ^13^C-NMR (CDCl_3_, 100 MHz): δ = 21.6, 51.0, 108.5, 111.4, 121.0, 123.6, 123.8, 130.5, 133.1, 136.5, 165.8 ppm. The values are in accordance with the literature [81].

*Methyl 4-chloro-1H-indole-3-carboxylate* (**6o**; C-4 isomer) (186.0 mg, 44%). Pale yellow solid: ^1^H-NMR (DMSO-d_6,_ 400 MHz) δ = 3.76 (s, 3H), 7.13 – 7.26 (m, 2H), 7.46 (dd, 1H, *J* = 6.7 and 2.3 Hz), 8.13 (d, 1H, *J* = 2.6 Hz), 12.18 (s, 1H). ^13^C-NMR (DMSO-d_6_, 100 MHz) δ = 51.8, 107.8, 112.4, 123.3, 123.6, 124.1, 125.8, 135.0, 139.3, 164.4. HRMS (ESI^-^) m/z [M − H]^−^ calculated for C_10_H_7_NO_2_Cl 208.0165, found 208.0163.

*Methyl 6-chloro-1H-indole-3-carboxylate* (**6o**; C-6 isomer) (175.6 mg, 42%). Pale yellow solid: ^1^H-NMR (CDCl_3,_ 400 MHz) δ = 3.94 (s, 3H), 7.26 (d, 1H, *J* = 9.4 Hz), 7.43 (s, 1H), 7.92 (s, 1H), 8.11 (d, 1H, *J* = 8.6 Hz), 8.60 (s, 1H). ^13^C-NMR (DMSO-*d_6_*_,_ 100 MHz) δ = 52.7, 108.3, 113.8, 123.4, 123.5, 125.9, 129.0, 135.1, 138.4, 166.7. The values are in accordance with the literature [62].

*Methyl 4-bromo-1H-indole-3-carboxylate* (**6p**; C-4 isomer) (258.8 mg, 51%). Pale yellow solid: ^1^H-NMR (DMSO-d_6,_ 400 MHz) δ = 3.77 (s, 3H), 7.11 (t, 1H, *J* = 7.9 Hz), 7.39 (d, 1H, *J* = 7.5 Hz), 7.51 (d, 1H, *J* = 7.8 Hz), 8.12 (s, 1H), 12.16 (s, 1H). ^13^C-NMR (DMSO-d_6,_ 100 MHz) δ = 51.7, 108.4, 112.9, 113.7, 124.4, 125.0, 127.1, 134.7, 139.0, 164.5. HRMS (ESI^+^) m/z [M + Na]^+^ calculated for C_10_H_8_NO_2_BrNa 275.9636, found 275.9642.

*Methyl 6-bromo-1H-indole-3-carboxylate* (**6p***;* C-6 isomer) (200.1 mg, 39%). Pale yellow solid: ^1^H-NMR (DMSO-d_6,_ 400 MHz) δ = 3.80 (s, 3H), 7.32 (dd, 1H, *J* = 8.5 and 1.8 Hz), 7.67 (s, 1H), 7.92 (d, 1H, *J* = 8.5 Hz), 8.11 (d, 1H, *J* = 2.8 Hz), 12.03 (s, 1H). ^13^C-NMR (DMSO-d_6,_ 100 MHz) δ = 51.7, 107.5, 115.9, 115.9, 123, 125.1, 125.6, 134.2, 138.1, 165.3. HRMS (ESI^-^) m/z [M − H]^−^ calculated for C_10_H_7_NO_2_Br 251.9660, found 251.9657.

*Methyl 4-acetyl-1H-indole-3-carboxylate* (**6q**; C-4 isomer) (539.8 mg, 62%). Off-white solid: ^1^H-NMR (DMSO-*d_6_*_,_ 400 MHz): δ = 2.45 (s, 3H), 3.72 (s, 3H), 7.18 (d, 1H, *J* = 7.2 Hz), 7.26 (t, 1H, *J* = 7.5 Hz), 7.60 (d, 1H, *J* = 7.9 Hz), 8.14 (s, 1H), 12.17 (bs, 1H). ^13^C-NMR (CDCl_3_, 100 MHz): δ = 31.1, 51.0, 108.2, 114.3, 119.8, 120.7, 122.5, 132.6, 135.3, 137.0, 165.2, 205.2 ppm. HRMS (ESI^+^) m/z [M + Na]^+^ calculated for C_12_H_11_NO_3_Na 240.0637, found 240.0634.

*Methyl 6-acetyl-1H-indole-3-carboxylate* (**6q**; C-6 isomer) (237.7 mg, 27%). Off-white solid: ^1^H-NMR (DMSO-d_6,_ 400 MHz): δ = 2.66 (s, 3H), 3.86 (s, 3H), 7.84 (dd, 1H, *J* = 8.4 and 1.5 Hz), 8.10 (d, 1H, *J* = 8.4 Hz), 8.15 (d, 1H, *J* = 1.5 Hz), 8.33 (d, 1H, *J* = 3.0 Hz), 12.39 (bs, 1H). ^13^C-NMR (DMSO-*d_6_*_,_ 100 MHz): δ = 26.7, 50.8, 106.7, 113.4, 120.1, 121.1, 129.9, 131.5, 135.7, 135.9, 164.4, 197.3 ppm. HRMS (ESI^+^) m/z [M+Na]^+^ calculated for C_12_H_11_NO_3_Na 240.0637, found 240.0640.

Methyl 4-methoxy-1*H*-indole-3-carboxylate (**6r**; C-4 isomer) (111.8 mg, 27%). Off-white solid: ^1^H-NMR (CDCl_3_, 400 MHz): δ = 3.86 (s, 3H), 3.93 (s, 3H), 6.66 (d, 1H, *J* = 7.8 Hz), 7.00 (d, 1H, *J* = 8.0 Hz), 7.16 (t, 1H, *J* = 7.0 Hz), 7.81 (d, 1H, *J* = 2.8 Hz), 9.11 (bs, 1H). ^13^C-NMR (CDCl_3_, 100 MHz): δ = 51.3, 55.9, 102.9, 105.0, 108.6, 115.4, 124.2, 131.3, 138.4, 154.2, 165.0 ppm. HRMS (ESI^+^) m/z [M + Na]^+^ calculated for C_11_H_11_NO_3_Na 228.0637, found 228.0634.

*Methyl 6-methoxy-1H-indole-3-carboxylate* (**6r**; C-6 isomer) (189.5 mg, 46%). Off-white solid: ^1^H-NMR (DMSO-d_6,_ 400 MHz): δ = 3.78 (s, 6H), 6.83 (dd, 1H, *J* = 8.6 and 2.2 Hz), 6.96 (d, 1H, *J* = 2.2 Hz), 7.84 (d, 1H, *J* = 8.6 Hz), 7.94 (d, 1H, *J* = 3.0 Hz), 11.70 (bs, 1H). ^13^C-NMR (DMSO-*d_6_*_,_ 100 MHz): δ = 50.5, 55.2, 95.2, 106.3, 111.3, 119.7, 121.0, 131.2, 137.1, 156.1, 164.7 ppm. The values are in accordance with the literature [82]. 

*Methyl 7-methyl-1H-indole-3-carboxylate* (**6s**) (245.9.5 mg, 65%). Off-white solid: ^1^H-NMR (CDCl_3_, 400 MHz): δ = 2.50 (s, 3H), 3.92 (s, 3H), 7.06 (d, 1H, *J* = 7.1 Hz), 7.19 (t, 1H, *J* = 7.6 Hz), 7.90 (d, 1H, *J* = 2.8 Hz), 8.02 (d, 1H, *J* = 8.0 Hz), 8.71 (bs, 1H). ^13^C-NMR (CDCl_3_, 100 MHz): δ = 16.5, 51.1, 109.2, 119.2, 120.7, 122.2, 123.8, 125.4, 130.7, 135.7, 165.8 ppm. The values are in accordance with the literature [83].

*Methyl 6,7-dimethyl-1H-indole-3-carboxylate* (**6t**) (235.0 mg, 58%). Off-white solid: ^1^H-NMR (DMSO-d_6_, 400 MHz): δ = 2.32 (s, 3H), 2.39 (s, 3H), 3.79 (s, 3H), 6.99 (d, 1H, *J* = 8.0 Hz), 7.71 (d, 1H, *J* = 8.0 Hz), 7.99 (d, 1H, *J* = 3.0 Hz), 11.78 (bs, 1H). ^13^C-NMR (DMSO-d_6_, 100 MHz): δ = 13.1, 18.9, 50.5, 106.6, 117.4, 119.4, 123.8, 123.9, 129.5, 131.6, 136.5, 164.8 ppm. HRMS (ESI^+^) m/z [M + Na]^+^ calculated for C_12_H_13_NO_2_Na 226.0844, found 226.0845.

*Methyl 6-chloro-7-methyl-1H-indole-3-carboxylate* (**6u**) (299.8 mg, 67%). Off-white solid: ^1^H-NMR (CDCl_3_, 400 MHz): δ = 2.52 (s, 3H), 3.91 (s, 3H), 7.26 (d, 1H, *J* = 8.6 Hz), 7.90 (d, 1H, *J* = 2.9 Hz), 7.93 (d, 1H, *J* = 8.6 Hz), 8.56 (bs, 1H). ^13^C-NMR (CDCl_3_, 100 MHz): δ = 13.8, 51.2, 109.6, 118.6, 119.8, 123.5, 124.1, 128.9, 131.0, 136.2, 165.3 ppm. HRMS (ESI^-^) m/z [M − H]^−^ calculated for C_11_H_9_ClNO_2_ 222.0322, found 222.0314.

*Methyl 4,6-dimethyl-1H-indole-3-carboxylate* (**6v**) (607.7 mg, 75%). Off-white solid: ^1^H-NMR (DMSO-d_6_, 400 MHz): δ = 2.33 (s, 3H), 2.70 (s, 3H), 3.73 (s, 3H), 6.74 (s, 1H), 7.07 (s, 1H), 7.96 (d, 1H, *J* = 3.0 Hz), 11.71 (bs, 1H). ^13^C-NMR (DMSO-d_6_, 100 MHz): δ = 20.9, 21.9, 50.6, 107.2, 109.7, 122.1, 124.9, 130.3, 131.6, 132.7, 137.6, 164.6 ppm. HRMS (ESI^-^) m/z [M − H]^−^ calculated for C_12_H_12_NO_2_ 202.0868, found 202.0872.

*Methyl 1,6,7,8-tetrahydrocyclopenta[g]indole-3-carboxylate* (**6w**) (305.6 mg, 71%). Off-white solid: ^1^H-NMR (DMSO-d_6_, 400 MHz): δ = 2.08–2.15 (m, 2H), 2.95 (t, 2H, *J* = 7.3 Hz), 3.04 (t, 2H, *J* = 7.3 Hz), 3.79 (s, 3H), 7.08 (d, 1H, *J* = 8.0 Hz), 7.79 (d, 1H, *J* = 8.0 Hz), 7.99 (d, 1H, *J* = 3.0 Hz), 11.88 (bs, 1H). ^13^C-NMR (DMSO-d_6_, 100 MHz): δ = 25.0, 29.9, 32.6, 50.6, 106.9, 118.1, 118.5, 124.4, 126.5, 131.6, 133.5, 138.3, 164.9 ppm. HRMS (ESI^+^) m/z [M + Na]^+^ calculated for C_13_H_13_NO_2_Na 238.0844, found 238.0848.

*Methyl 7-chloro-1H-indole-3-carboxylate* (**6x**) (175.7 mg, 21%). Pale yellow solid: ^1^H-NMR (DMSO-d_6_, 400 MHz): δ = 3.82 (s, 3H), 7.18 (t, 1H, *J* = 7.8 Hz), 7.29 (dd, 1H, *J* = 7.7 and 1.1 Hz), 7.98 (d, 1H, *J* = 7.9 Hz), 8.11 (d, 1H, *J* = 3.1 Hz), 12.36 (s, 1H). ^13^C-NMR (DMSO-d_6,_ 100 MHz) δ = 51.7, 108.6, 117.6, 120.4, 122.9, 123.3, 128.4, 134.1, 134.2, 165.3. HRMS (ESI^-^) m/z [M − H]^−^ calculated for C_10_H_7_NO_2_Cl 208.0165, found 208.0158.

*Methyl 7-fluoro-1H-indole-3-carboxylate* (**6y**) (285.6 mg, 37%). Off-white solid: ^1^H-NMR (DMSO-d_6_, 400 MHz): δ = 3.81 (s, 3H), 7.05 (dd, 1H, *J* = 11.5 and 8.0 Hz), 7.13–7.18 (m, 1H), 7.81 (d, 1H, *J* = 8.0 Hz), 8.12 (s, 1H), 12.49 (bs, 1H). ^13^C-NMR (DMSO-d_6_, 100 MHz): δ = 50.8, 107.3 (d, *J* = 16 Hz), 107.4, 116.5 (d, *J* = 3.5 Hz), 121.8 (d, *J* = 6.3 Hz), 124.2 (d, *J* = 13.5 Hz), 129.3 (d, *J* = 5.3 Hz), 133.1, 149.2 (d, *J* = 244 Hz), 164.4 ppm. HRMS (ESI^-^) m/z [M − H]^−^ calculated for C_10_H_7_FNO_2_ 192.0461, found 192.0457.

*Methyl 7-methoxy-1H-indole-3-carboxylate* (**6z**) (90.4 mg, 11%). Off-white solid: ^1^H-NMR (CDCl_3_, 500 MHz): δ = 3.91 (s, 3H), 3.94 (s, 3H), 6.70 (d, 1H, *J* = 7.8 Hz), 7.17 (t, 1H, *J* = 8.0 Hz), 7.75 (d, 1H, *J* = 8.0 Hz), 7.87 (d, 1H, *J* = 3.0 Hz), 8.90 (bs, 1H). ^13^C-NMR (CDCl_3_, 125 MHz): δ = 51.0, 55.4, 102.9, 109.2, 113.9, 122.5, 126.7, 127.1, 130.3, 146.1, 165.7 ppm. The values are in accordance with the literature [84].

*Methyl 7-chloro-4-fluoro-1H-indole-3-carboxylate* (**6aa**) (245.2 mg, 27%). Off-white solid: ^1^H-NMR (CDCl_3_, 400 MHz): δ = 3.90 (s, 3H), 6.87 (dd, 1H, *J* = 10.5 and 8.5 Hz), 7.16 (dd, 1H, *J* = 8.5 and 3.6 Hz), 7.96 (d, 1H, *J* = 3.0 Hz), 9.13 (bs, 1H). ^13^C-NMR (CDCl_3_, 100 MHz): δ = 51.8, 108.8 (d, *J* = 23 Hz), 109.4 (d, *J* = 3.5 Hz), 112.1 (d, *J* = 4.1 Hz), 115.1 (d, *J* = 22 Hz), 123.0 (d, *J* = 8.0 Hz), 132.2, 135.6 (d, *J* = 10.0 Hz), 155.1 (d, *J* = 253 Hz), 164.0 ppm. HRMS (ESI^+^) m/z [M + Na]^+^ calculated for C_10_H_7_ClFNO_2_Na 250.0047, found 250.0051.

*Methyl 4,7-dibromo-1H-indole-3-carboxylate* (**6ab**) (598.0 mg, 45%). Off-white solid: ^1^H-NMR (DMSO-*d_6_*_,_ 500 MHz): δ = 3.78 (s, 3H), 7.33 (d, 1H, *J* = 8.0 Hz), 7.37 (d, 1H, *J* = 8.0 Hz), 8.09 (d, 1H, *J* = 3.1 Hz), 12.40 (bs, 1H). ^13^C-NMR (DMSO-d_6,_ 125 MHz): δ = 51.1, 104.6, 109.1, 112.3, 125.3, 126.1, 127.3, 134.3, 136.0, 163.3 ppm. HRMS (ESI^+^) m/z [M + Na]^+^ calculated for C_10_H_7_Br_2_NO_2_Na 357.8700, found 357.8698.

*Methyl 1H-benzo[g]indole-3-carboxylate* (**6ac**) (745.8 mg, 83%). Yellow-brown solid: ^1^H-NMR (DMSO-d_6,_ 400 MHz): δ = 3.85 (s, 3H), 7.48 (t, 1H, *J* = 7.5 Hz), 7.60 (t, 1H, *J* = 7.5 Hz), 7.65 (d, 1H, *J* = 8.7 Hz), 7.97 (d, 1H, *J* = 8.2 Hz), 8.10–8.19 (m, 2H), 8.44 (d, 1H, *J* = 8.2 Hz), 12.84 (s, 1H). ^13^C-NMR (DMSO-*d_6_*_,_ 100 MHz): δ = 51.6, 109.1, 121.0, 121.6, 122.8, 122.9, 122.9, 125.3, 126.8, 129.3, 130.9, 130.9, 132.1, 165.8. The values are in accordance with the literature [85].

*(6-Methoxy-1H-indol-3-yl)(3,4,5-trimethoxyphenyl)methanone* (**6ad**; C-6 isomer) (257.3 mg, 30%). Off-white solid: ^1^H-NMR (CDCl_3_, 500 MHz): δ = 3.79 (s, 3H), 3.83 (s, 6H), 3.91 (s, 3H), 6.87 (d, 1H, *J* = 2.2 Hz), 6.93 (dd, 1H, *J* = 8.7 and 2.2 Hz), 7.07 (s, 2H), 7.59 (d, 1H, *J* = 3.0 Hz), 8.21 (d, 1H, *J* = 8.7 Hz), 9.48 (bs, 1H). ^13^C-NMR (CDCl_3_, 125 MHz): δ = 55.5, 56.2 (2C), 60.9, 95.1, 106.4 (2C), 112.2, 116.7, 120.4, 122.9, 132.9, 135.9, 137.5, 140.9, 152.9 (2C), 157.5, 190.6 ppm. The values are in accordance with the literature [20].

*(4-Methoxy-1H-indol-3-yl)(3,4,5-trimethoxyphenyl)methanone* (**6ad**; C-4 isomer) (212.1 mg, 25%). Off-white solid: ^1^H-NMR (DMSO-d_6,_ 500 MHz): δ = 3.65 (s, 3H), 3.75 (s, 3H), 3.77 (s, 6H), 6.63 (d, 1H, *J* = 7.8 Hz), 7.09 (s, 2H), 7.10 (d, 1H, *J* = 7.8 Hz), 7.16 (t, 1H, *J* = 7.9 Hz), 7.75 (d, 1H, *J* = 3.0 Hz), 11.86 (bs, 1H). ^13^C-NMR (DMSO-*d_6_*, 125 MHz): δ = 55.0, 55.9 (2C), 60.1, 101.9, 105.2, 107.0 (2C), 115.7, 115.9, 123.7, 131.6, 135.3, 138.2, 140.7, 152.3 (2C), 153.6, 188.3 ppm. The values are in accordance with the literature [20].

*(±)-Methyl (2R,3R,3aS)-2-hydroxy-3a-methyl-3,3a-dihydro-2H-indole-3-carboxylate* (**10s**) (144.1 mg, 21%). Off-white solid: ^1^H-NMR (CDCl_3_, 400 MHz): δ = 1.00 (s, 3H), 2.95 (d, 1H, *J* = 7.4 Hz), 3.79 (s, 3H), 5.7 (bs, 1H), 5.89 (d, 1H, *J* = 7.4 Hz), 6.09–6.13 (m, 1H), 6.44–6.47 (m, 2H), 6.58–6.62 (m, 1H). ^13^C-NMR (CDCl_3_, 100 MHz): δ = 20.0, 52.1, 53.3, 61.8, 91.0, 120.7, 121.3, 136.0, 140.4, 170.5, 178.3 ppm. HRMS (ESI^+^) m/z [M + Na]^+^ calculated for C_11_H_13_NO_3_Na 230.0793, found 230.0785.

*(±)-(2R,3R,3aR)-Methyl 2-hydroxy-3a,4-dimethyl-3,3a-dihydro-2H-indole-3-carboxylate* (**10t**) (150.6 mg, 34%). Off-white solid: ^1^H-NMR (CDCl_3_, 500 MHz): δ = 1.13 (s, 3H), 1.91 (s, 3H), 2.91 (d, 1H, *J* = 7.4 Hz), 3.77 (s, 3H), 5.4 (bs, 1H), 5.80 (d, 1H, *J* = 6.0 Hz), 5.85 (d, 1H, *J* = 7.4 Hz), 6.27 (d, 1H, *J* = 9.7 Hz), 6.50 (dd, 1H, *J* = 9.7 and 6.0 Hz). ^13^C-NMR (CDCl_3_, 125 MHz): δ = 15.4, 20.1, 52.0, 57.9, 60.5, 94.1, 118.4, 118.6, 136.5, 150.3, 171.6, 179.3 ppm. HRMS (ESI^+^) m/z [M + Na]^+^ calculated for C_12_H_15_NO_3_Na 244.0950, found 244.0952.

*(±)-(2R,3R,3aS)-Methyl 4-chloro-2-hydroxy-3a-methyl-3,3a-dihydro-2H-indole-3-carboxylate* (**10u**) (128.8 mg, 27%). Off-white solid: ^1^H-NMR (CDCl_3_, 500 MHz): δ = 1.30 (s, 3H), 3.02 (d, 1H, *J* = 7.5 Hz), 3.77 (s, 3H), 5.7 (bs, 1H), 5.75 (d, 1H, *J* = 7.5 Hz), 6.16 (d, 1H, *J* = 6.3 Hz), 6.42 (d, 1H, *J* = 9.6 Hz), 6.55 (dd, 1H, *J* = 9.6 and 6.3 Hz). ^13^C-NMR (CDCl_3_, 125 MHz): δ = 19.8, 52.2, 59.9, 61.0, 93.8, 119.5, 120.4, 135.5, 144.5, 170.8, 177.5 ppm. HRMS (ESI^+^) m/z [M + Na]^+^ calculated for C_11_H_12_ClNO_3_ 264.0403, found 264.0404.

The relative stereochemistry was assigned based on the ^1^HNMR, COSY, HSQC and NOESY experiments. The *syn* relationship between H(2) and H(3) was assigned based on the observed ^3^*J*(H,H) = 7.5 Hz and their NOE interactions. The *syn* relationship between Me(3a) and H(3) was assigned based on their NOE interactions.

*(±)-Methyl (2R,3R,3aR)-2-hydroxy-3a-methoxy-3,3a-dihydro-2H-indole-3-carboxylate* (**10z**) (195.8 mg, 22%). Off-white solid: ^1^H-NMR (CDCl_3_, 500 MHz): δ = 2.87 (d, 1H, *J* = 6.1 Hz), 3.03 (s, 3H), 3.79 (s, 3H), 3.9 (bs, 1H), 6.21 (d, 1H, *J* = 6.1 Hz), 6.43 (d, 1H, *J* = 9.6 Hz), 6.51 (ddd, 1H, *J* = 9.6, 5.5 and 1.0 Hz), 6.58–6.62 (m, 2H), 6.67 (d, 1H, *J* = 9.6 Hz). ^13^C-NMR (CDCl_3_, 125 MHz): δ = 52.2, 52.4, 65.4, 81.4, 91.8, 123.3, 127.4, 131.4, 134.4, 168.6, 172.3 ppm. HRMS (ESI^+^) m/z [M + Na]^+^ calculated for C_11_H_13_NO_4_Na 246.0742, found 246.0735.

## 4. Conclusions

In summary, we reported a practical (scalable) and instrumentally simple methodology to synthesize N-unprotected polysubstituted indoles endowed with an electron-withdrawing group at C-3 position. The importance of these N-unprotected heterocycles in organic synthesis (molecular platforms), asymmetric catalysis (chiral catalysts), medicinal chemistry (therapeutics) and materials sciences (molecular devices) ensures the practical use of this methodology in different fields of organic chemistry. Studies aimed at the use of these 3-EWG-indoles as molecular platforms for catalytic asymmetric dearomatization and their therapeutic/biological annotations are in progress in our lab.

## Figures and Tables

**Table 1:**
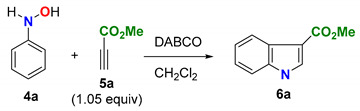
Optimization of the reaction conditions.

entry	DBACO	Temperature	Time	Yield (6a)
1	10 mol%	RT	1 h	68%
2	10 mol%	0 °C to RT	1 h	83%
3	1 mol%	0 °C to RT	Overnight	75%
4	5 mol%	0 °C to RT	1 h	82%
5	5 mol%	−25 °C to RT	2 h	86%

Red: To highlight that these are the best conditions.

## Supplementary Materials

^1^H and ^13^C-NMR spectra for compounds **6** and **10** are available online.
