# Supplementary material for: A General and Scalable Synthesis of Polysubstituted Indoles
Source: Molecules. 2020 Nov 28;25(23):5595. doi: 10.3390/molecules25235595 (PMC7730962; doi:10.3390/molecules25235595)
Supplement: Supplementary file 1 [file molecules-25-05595-s001.pdf]

Supporting Information

A general and scalable synthesis of polysubstituted indoles.

David Tejedor,*[a] Raquel Diana-Rivero[ab] and Fernando García Tellado*[a]

[a]*Instituto de Productos Naturales y Agrobiología, Consejo Superior de Investigaciones Científicas, Avda. Astrofísico Francisco Sánchez 3, 38206 La Laguna, Tenerife, Islas Canarias, Spain*

[b]*Doctoral and Postgraduate School, Universidad de La Laguna, Apartado Postal 456, 38200 La Laguna, Tenerife, Spain.*

fgarcia@ipna.csic.es; dtejedor@ipna.csic.es

Contents: Page

General information S1

^{1}H and ^{13}C spectra S2-S42

General information. ^{1}H NMR and ^{13}C NMR spectra of DMSO-d6 or $CDCl_3$ solutions were recorded either at 400 and 100 MHz or at 500 and 125 MHz (Bruker Ac 200 and AMX2-500 respectively).

Compound	Solvent	Field
**6a, 6c, 6f, 6h, 6j, 6l, 6n (C-4 and C-6 isomers), 6o (C-6 isomer), 6r (C-4 isomer), 6s, 6u, 6z, 6aa, 10s**	$CDCl_3$	400/100 MHz
**6d, 6e, 6f, 6i, 6k, 6m, 6o (C-4 isomer), 6p (C-4 and C-6 isomers), 6q (C-4 and C-6 isomers), 6r (C-6 isomer), 6t, 6v, 6x, 6w, 6y, 6ac**	DMSO-d6	400/100 MHz
**6z, 10t, 10u, 10z**	$CDCl_3$	500/125 MHz
**6b, 6g, 6ab, 6ad (C-4 and C-6 isomers)**	DMSO-d6	500/125 MHz

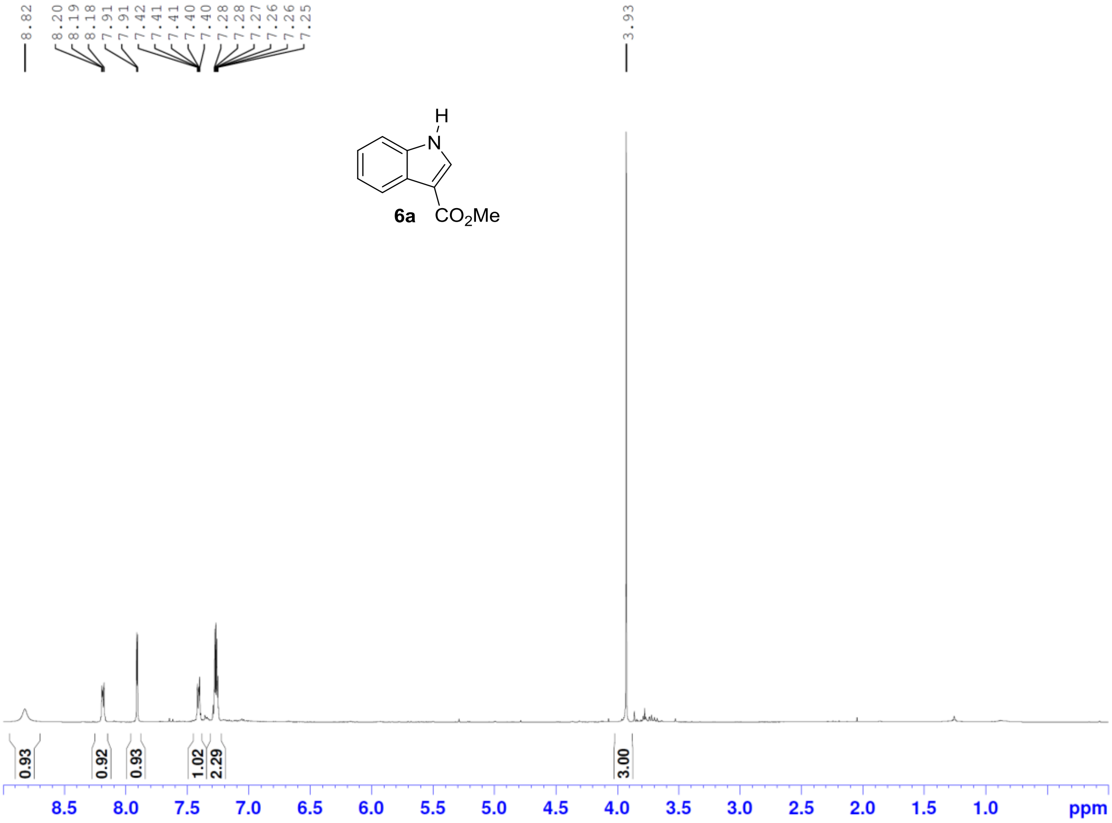
8.82
8.20
8.19
8.18
7.91
7.91
7.42
7.41
7.41
7.40
7.40
7.28
7.28
7.27
7.26
7.26
7.25
3.93
H
N
6a
CO_2Me
0.93
0.92
0.93
1.02
2.29
3.00
8.5 8.0 7.5 7.0 6.5 6.0 5.5 5.0 4.5 4.0 3.5 3.0 2.5 2.0 1.5 1.0 ppm

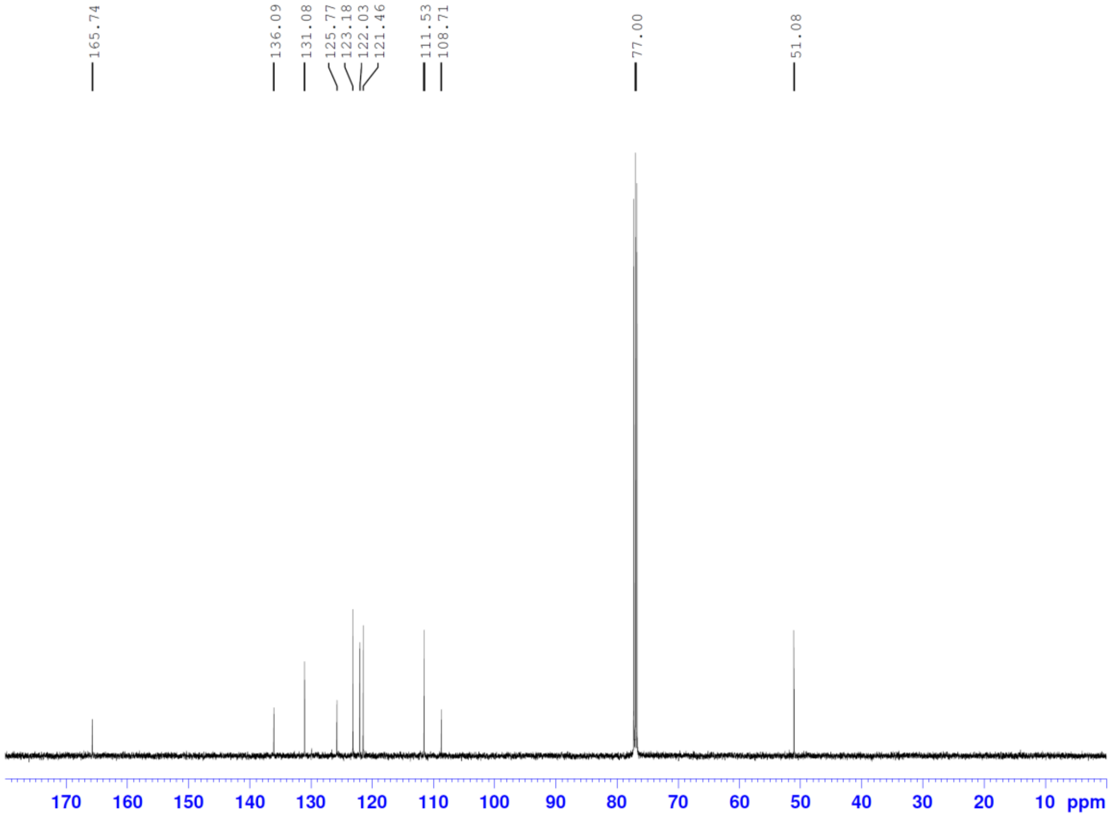
165.74
136.09
131.08
125.77
123.18
122.03
121.46
111.53
108.71
77.00
51.08
170 160 150 140 130 120 110 100 90 80 70 60 50 40 30 20 10 ppm

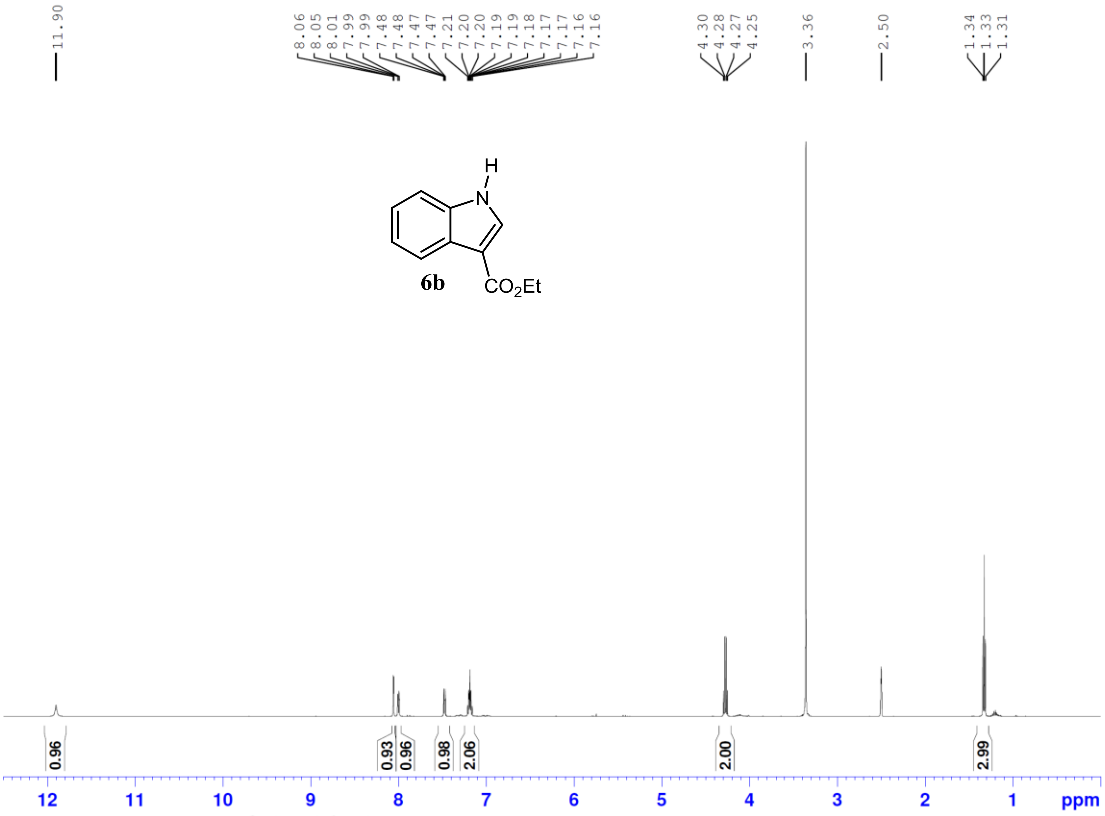
11.90
8.06 8.05 8.01 7.99 7.99 7.48 7.48 7.47 7.47 7.21 7.20 7.20 7.19 7.19 7.18 7.17 7.17 7.16 7.16
4.30 4.28 4.27 4.25
3.36
2.50
1.34 1.33 1.31
H
N
6b
CO_2Et
0.96
0.93
0.96
0.98
2.06
2.00
2.99
12 11 10 9 8 7 6 5 4 3 2 1 ppm

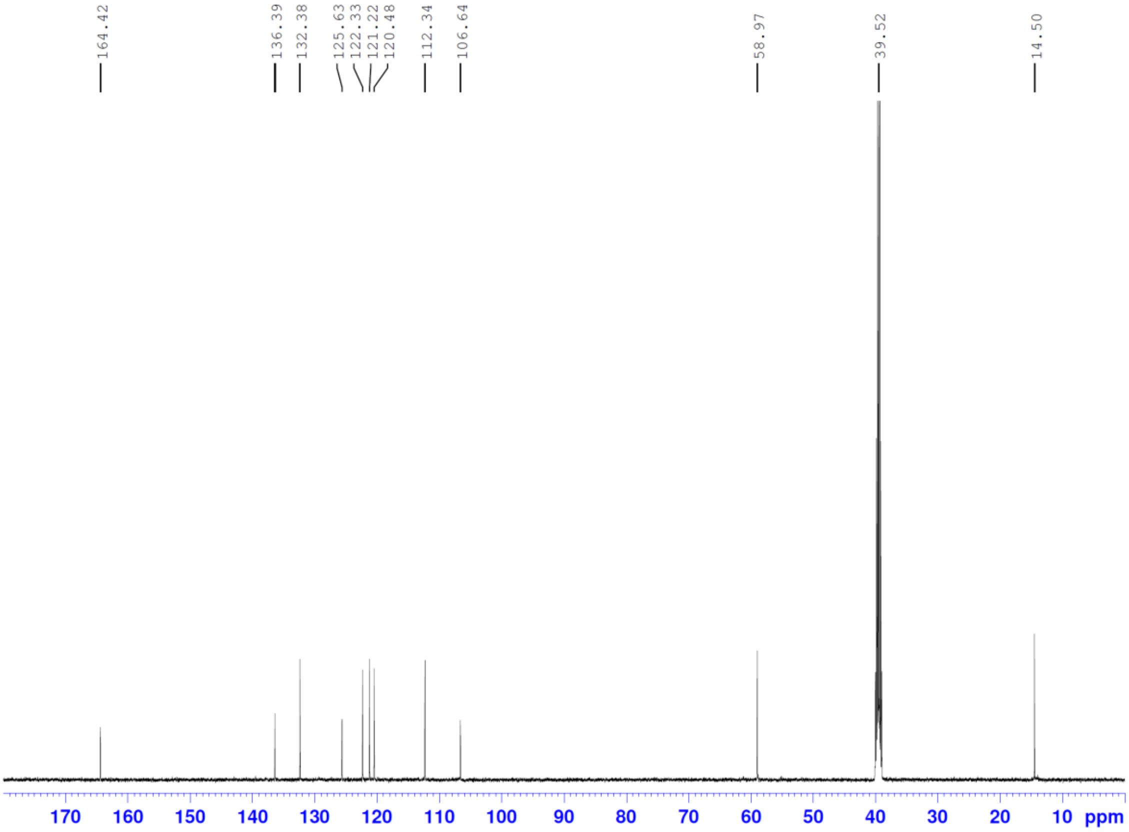
164.42
136.39
132.38
125.63
122.33
121.22
120.48
112.34
106.64
58.97
39.52
14.50
170 160 150 140 130 120 110 100 90 80 70 60 50 40 30 20 10 ppm

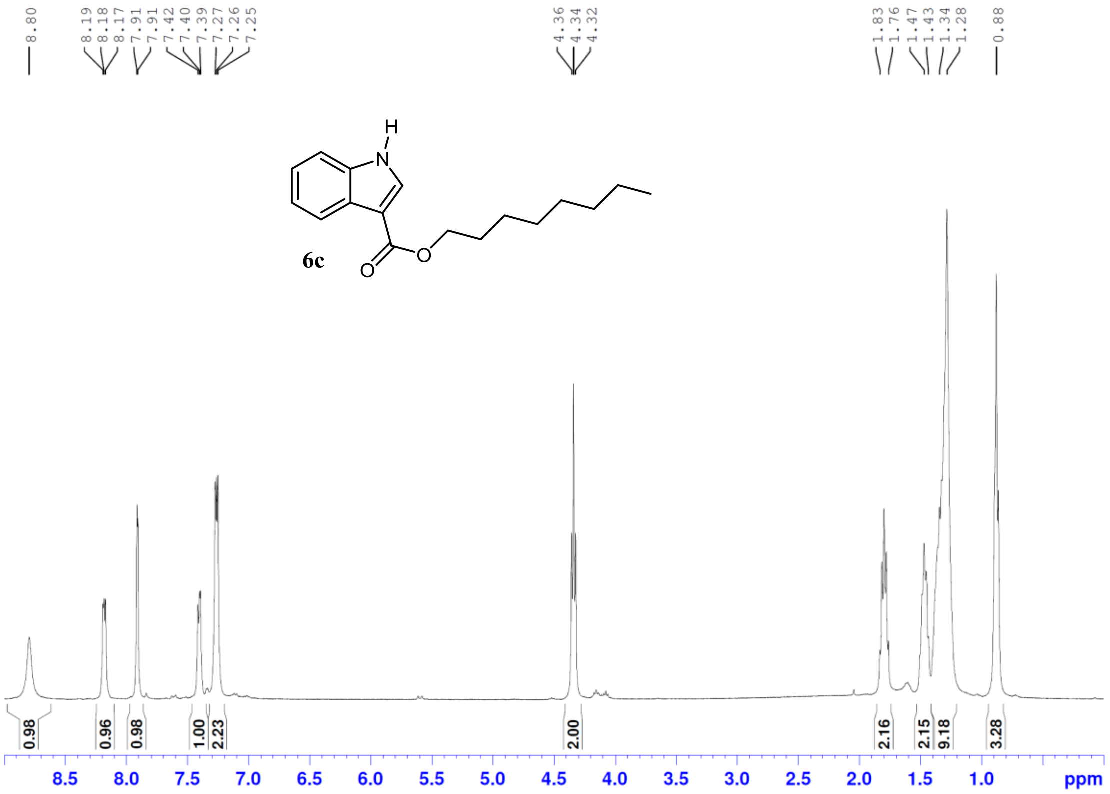
8.80
8.19
8.18
8.17
7.91
7.91
7.42
7.40
7.39
7.27
7.26
7.25
4.36
4.34
4.32
1.83
1.76
1.47
1.43
1.34
1.28
0.88
0.98
0.96
0.98
1.00
2.23
2.00
2.16
2.15
9.18
3.28
8.5 8.0 7.5 7.0 6.5 6.0 5.5 5.0 4.5 4.0 3.5 3.0 2.5 2.0 1.5 1.0 ppm
H
N
O
O

6c

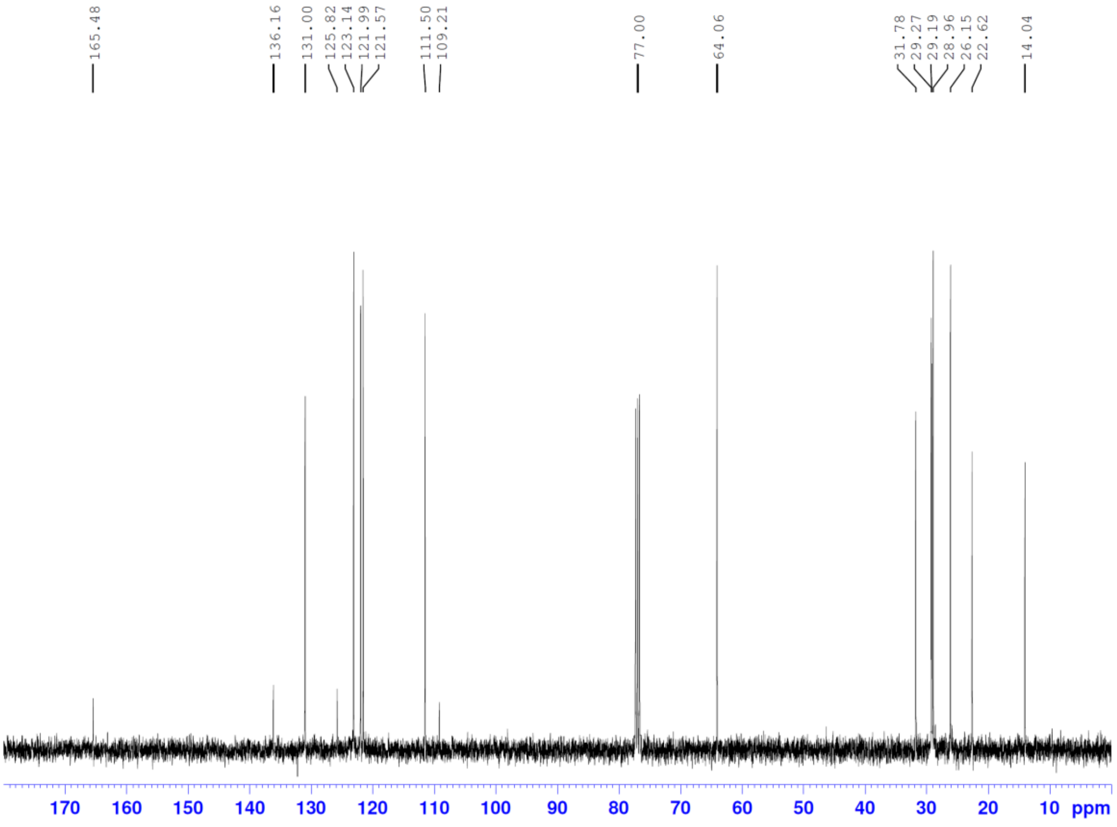
165.48
136.16
131.00
125.82
123.14
121.99
121.57
111.50
109.21
77.00
64.06
31.78
29.27
29.19
28.96
26.15
22.62
14.04
170 160 150 140 130 120 110 100 90 80 70 60 50 40 30 20 10 ppm

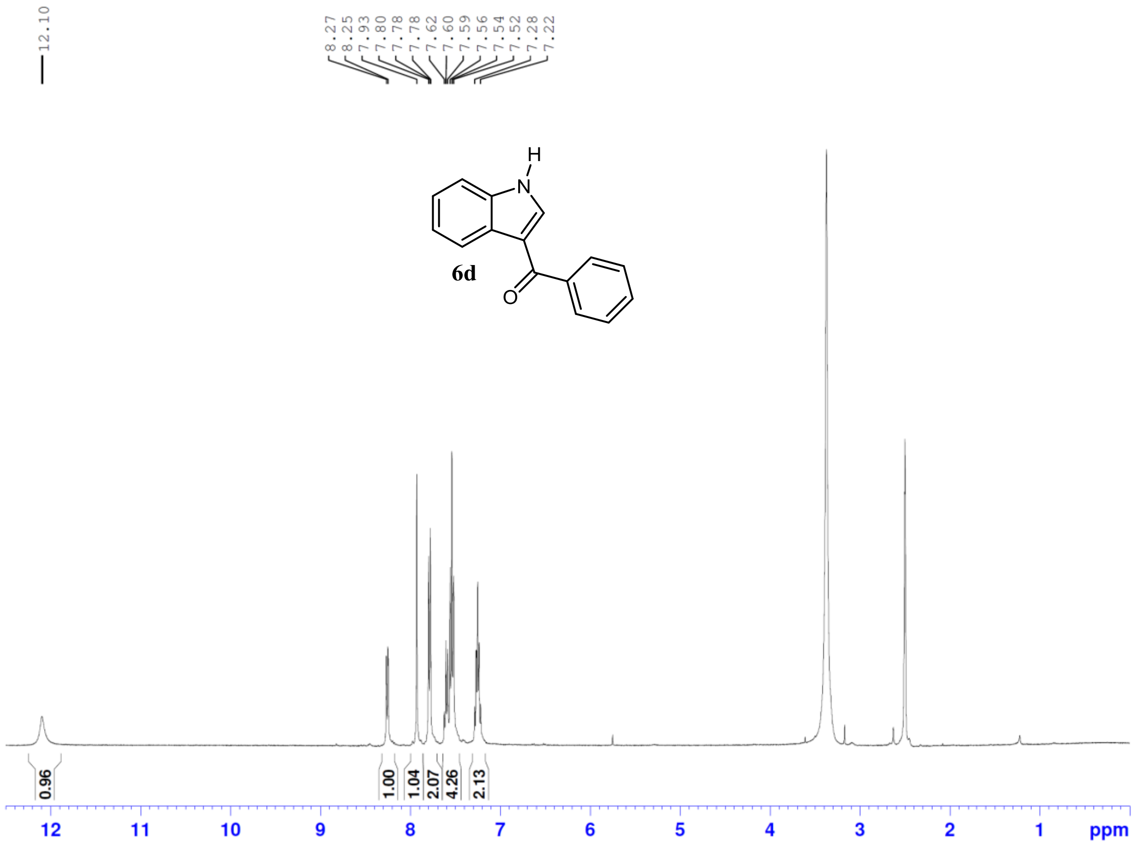
12.10
8.27
8.25
7.93
7.80
7.78
7.78
7.62
7.60
7.59
7.56
7.54
7.52
7.28
7.22
H
N
O
6d
0.96
1.00
1.04
2.07
4.26
2.13
12 11 10 9 8 7 6 5 4 3 2 1 ppm

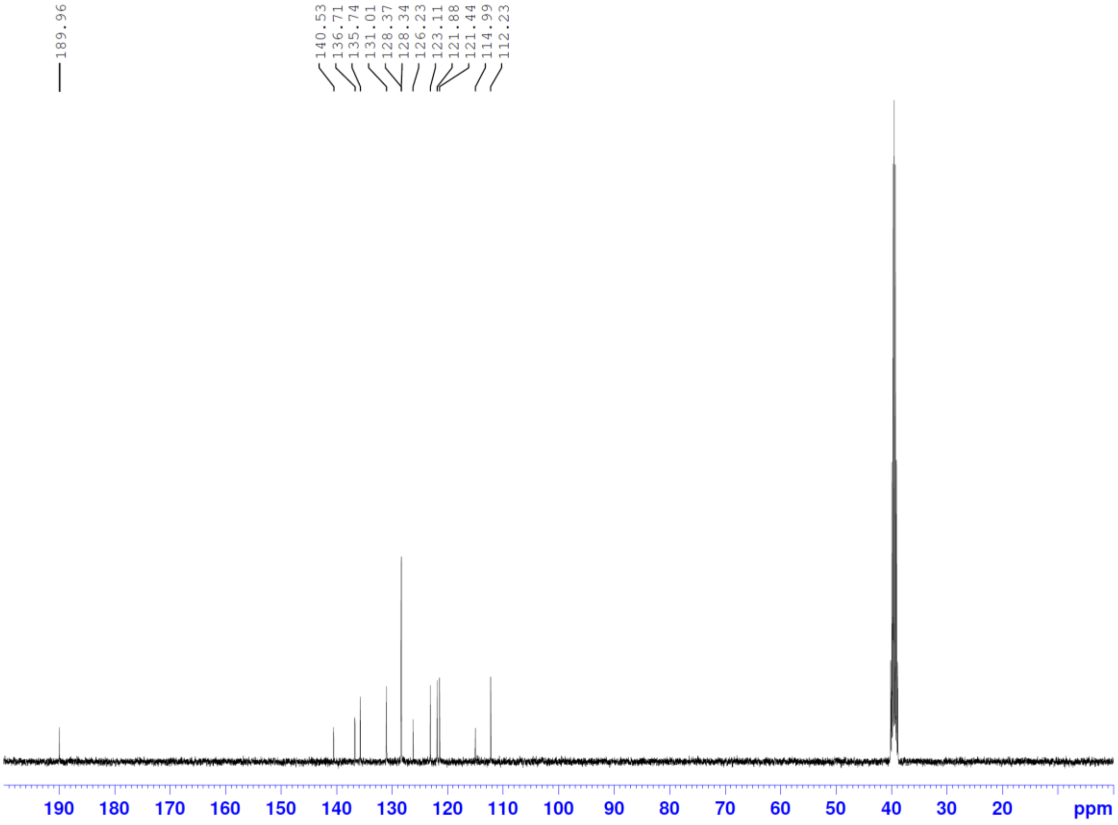
189.96
140.53
136.71
135.74
131.01
128.37
128.34
126.23
123.11
121.88
121.44
114.99
112.23
190 180 170 160 150 140 130 120 110 100 90 80 70 60 50 40 30 20 ppm

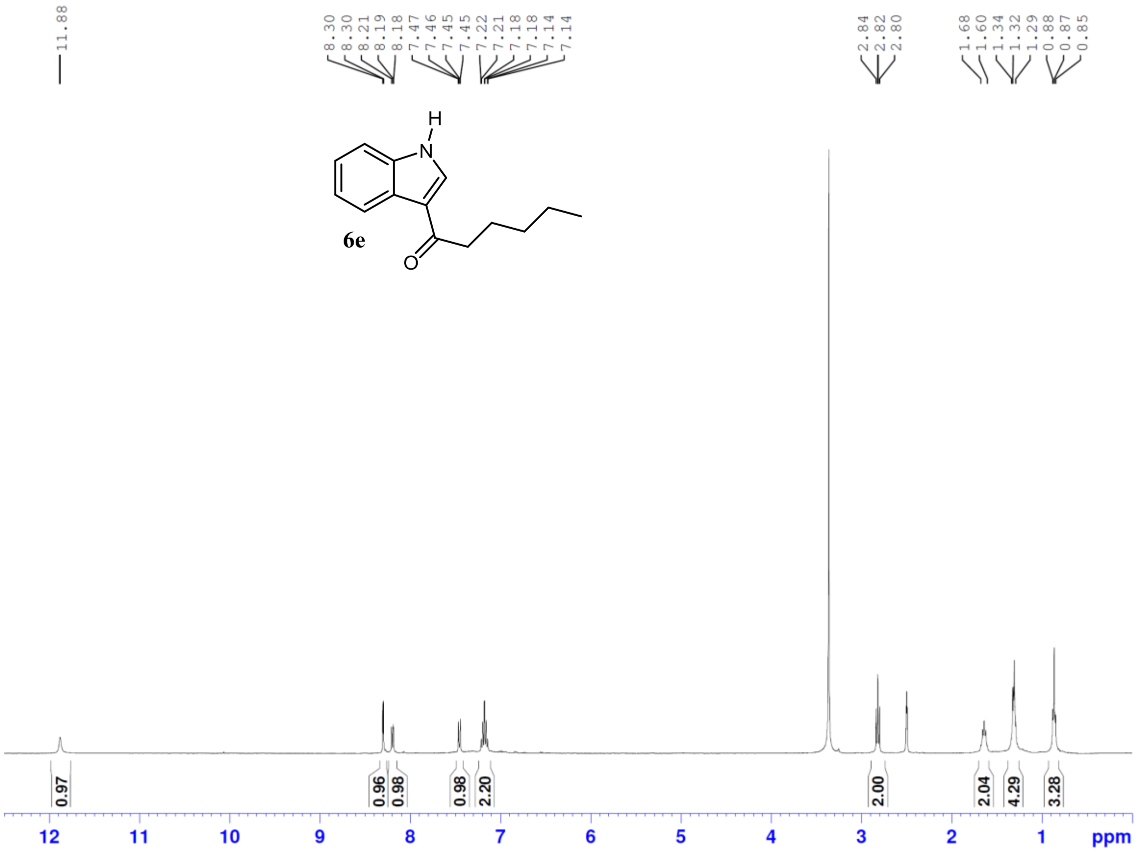
11.88
8.30 8.30 8.21 8.19 8.18 7.47 7.46 7.45 7.45 7.22 7.21 7.18 7.18 7.14 7.14
2.84 2.82 2.80
1.68 1.60 1.34 1.32 1.29 0.88 0.87 0.85
6e
0.97 0.96 0.98 0.98 2.20 2.00 2.04 4.29 3.28
12 11 10 9 8 7 6 5 4 3 2 1 ppm

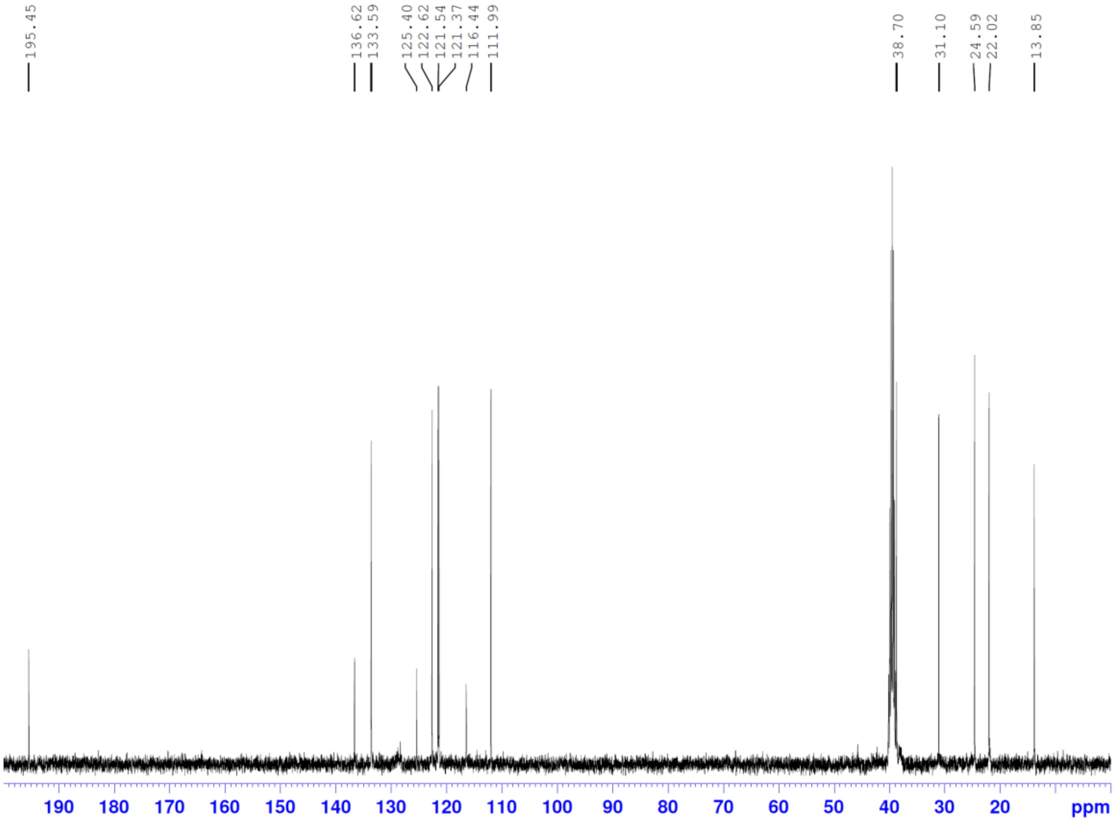
195.45
136.62 133.59 125.40 122.62 121.54 121.37 116.44 111.99
38.70 31.10 24.59 22.02 13.85
190 180 170 160 150 140 130 120 110 100 90 80 70 60 50 40 30 20 ppm

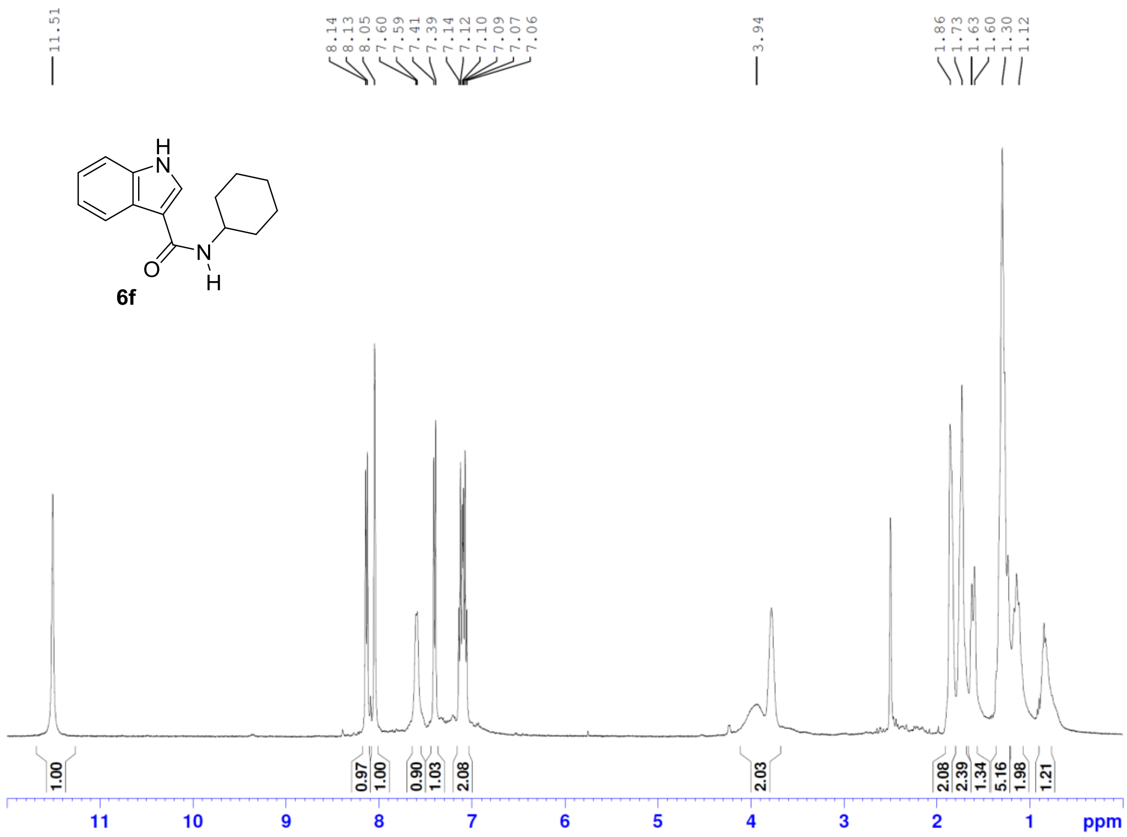
11.51
8.14
8.13
8.05
7.60
7.59
7.41
7.39
7.14
7.12
7.10
7.09
7.07
7.06
3.94
1.86
1.73
1.63
1.60
1.30
1.12
6f
1.00
0.97
1.00
0.90
1.03
2.08
2.03
2.08
2.39
1.34
5.16
1.98
1.21
11
10
9
8
7
6
5
4
3
2
1
ppm

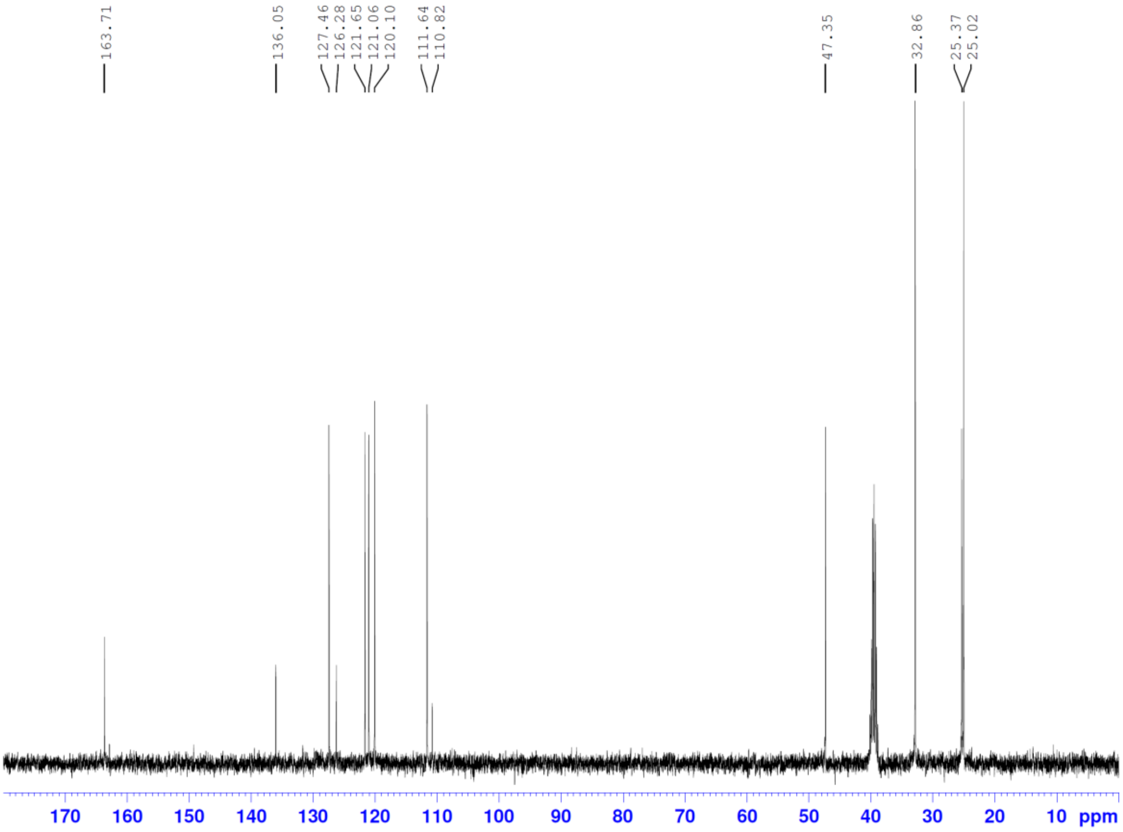
163.71
136.05
127.46
126.28
121.65
121.06
120.10
111.64
110.82
47.35
32.86
25.37
25.02
170
160
150
140
130
120
110
100
90
80
70
60
50
40
30
20
10
ppm

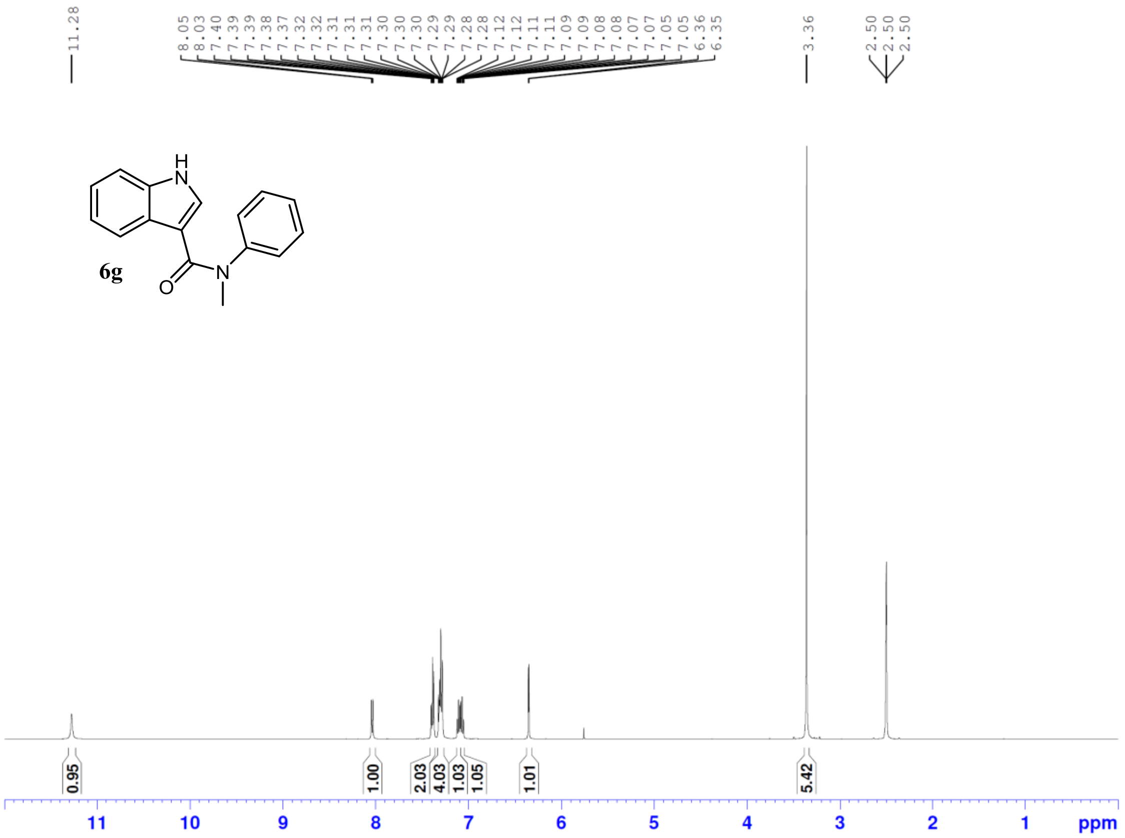
11.28
8.05 8.03 7.40 7.39 7.39 7.38 7.37 7.32 7.32 7.31 7.31 7.30 7.30 7.29 7.29 7.28 7.28 7.12 7.12 7.11 7.11 7.09 7.09 7.08 7.08 7.07 7.07 7.05 7.05 6.36 6.35
3.36
2.50 2.50 2.50
6g
0.95
1.00
2.03
4.03
1.03
1.05
1.01
5.42
11 10 9 8 7 6 5 4 3 2 1 ppm

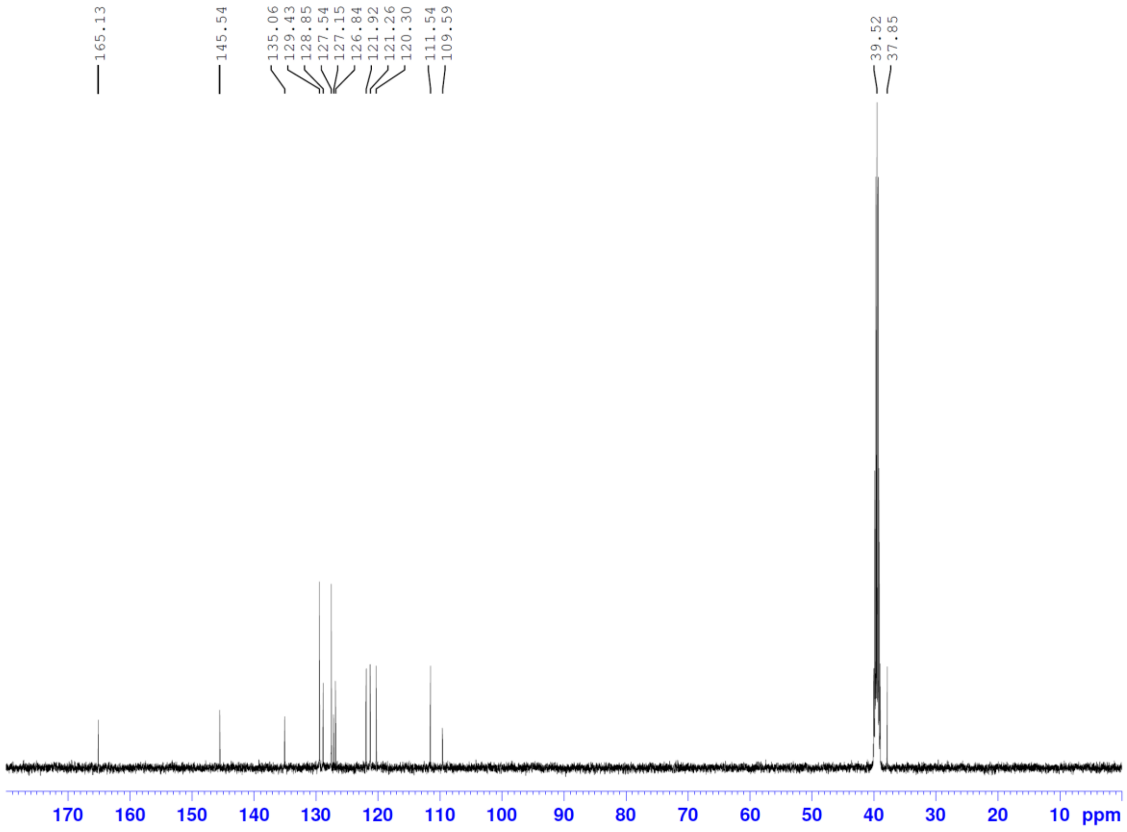
165.13
145.54
135.06 129.43 128.85 127.54 127.15 126.84 121.92 121.26 120.30
111.54 109.59
39.52 37.85
170 160 150 140 130 120 110 100 90 80 70 60 50 40 30 20 10 ppm

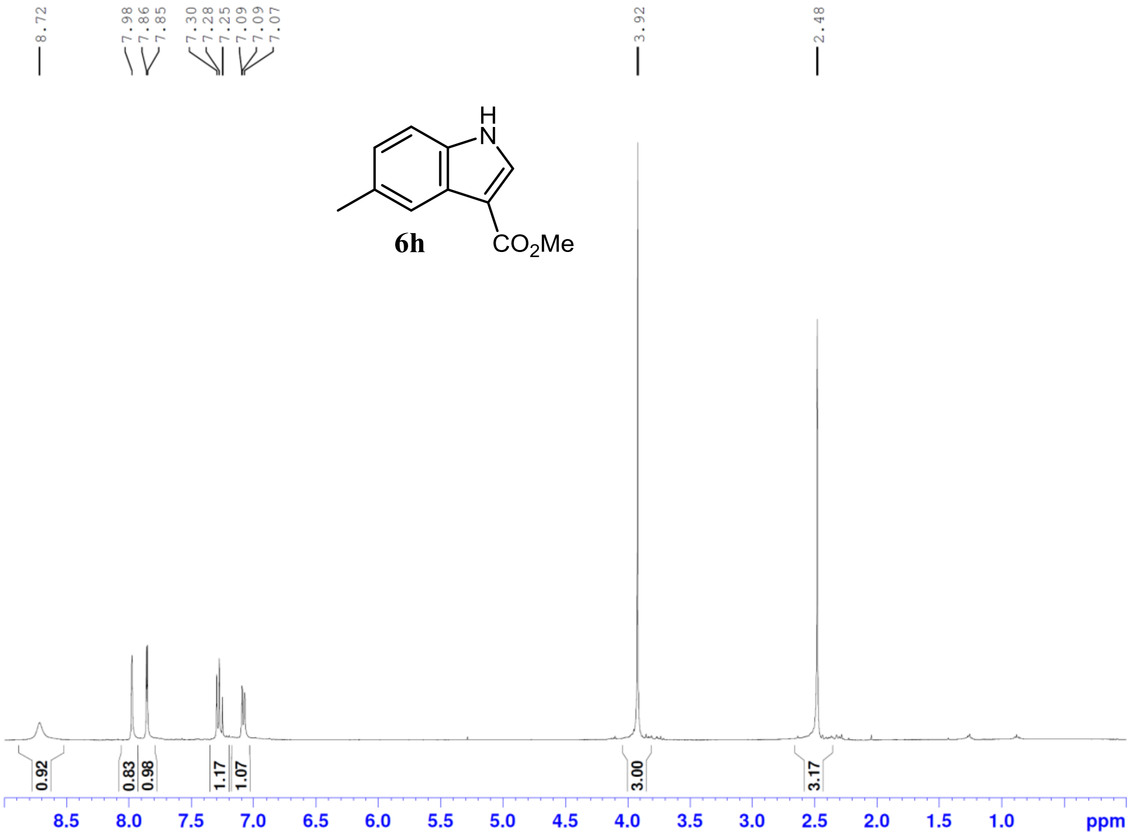
8.72
7.98
7.86
7.85
7.30
7.28
7.25
7.09
7.09
7.07
3.92
2.48
H
N
6h
CO2Me
0.92
0.83
0.98
1.17
1.07
3.00
3.17
8.5 8.0 7.5 7.0 6.5 6.0 5.5 5.0 4.5 4.0 3.5 3.0 2.5 2.0 1.5 1.0 ppm

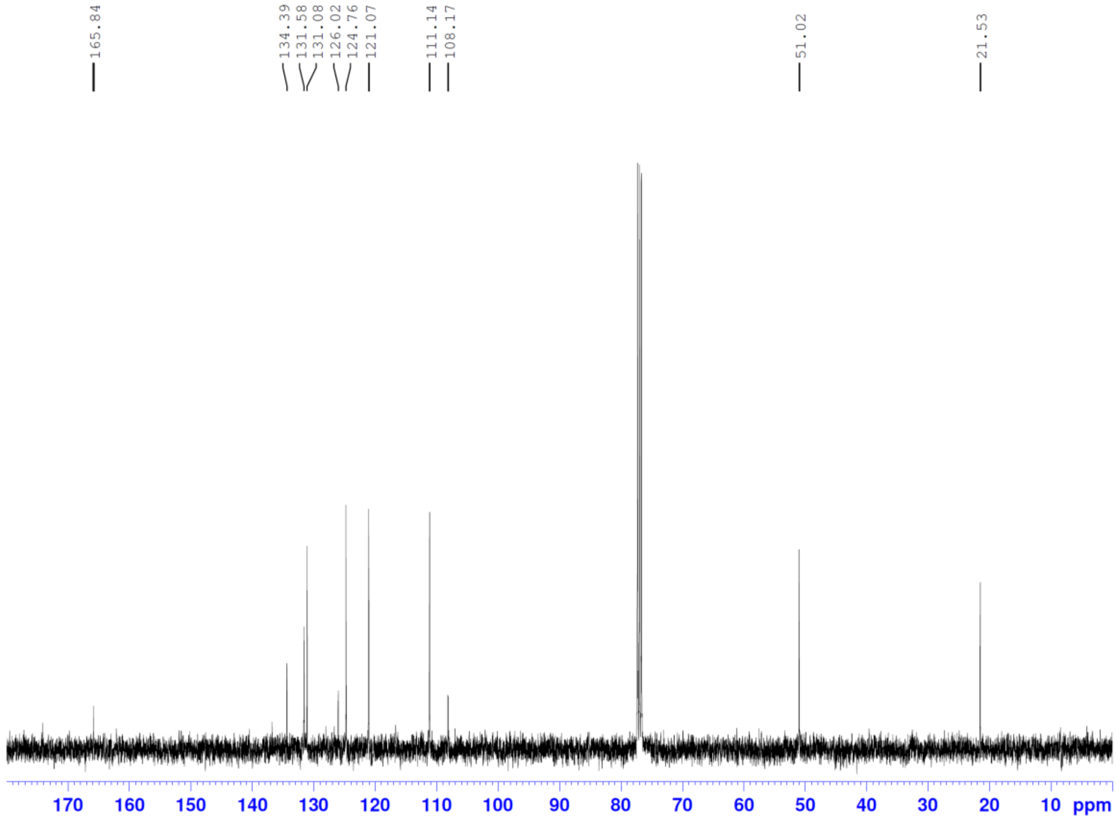
165.84
134.39
131.58
131.08
126.02
124.76
121.07
111.14
108.17
51.02
21.53
170 160 150 140 130 120 110 100 90 80 70 60 50 40 30 20 10 ppm

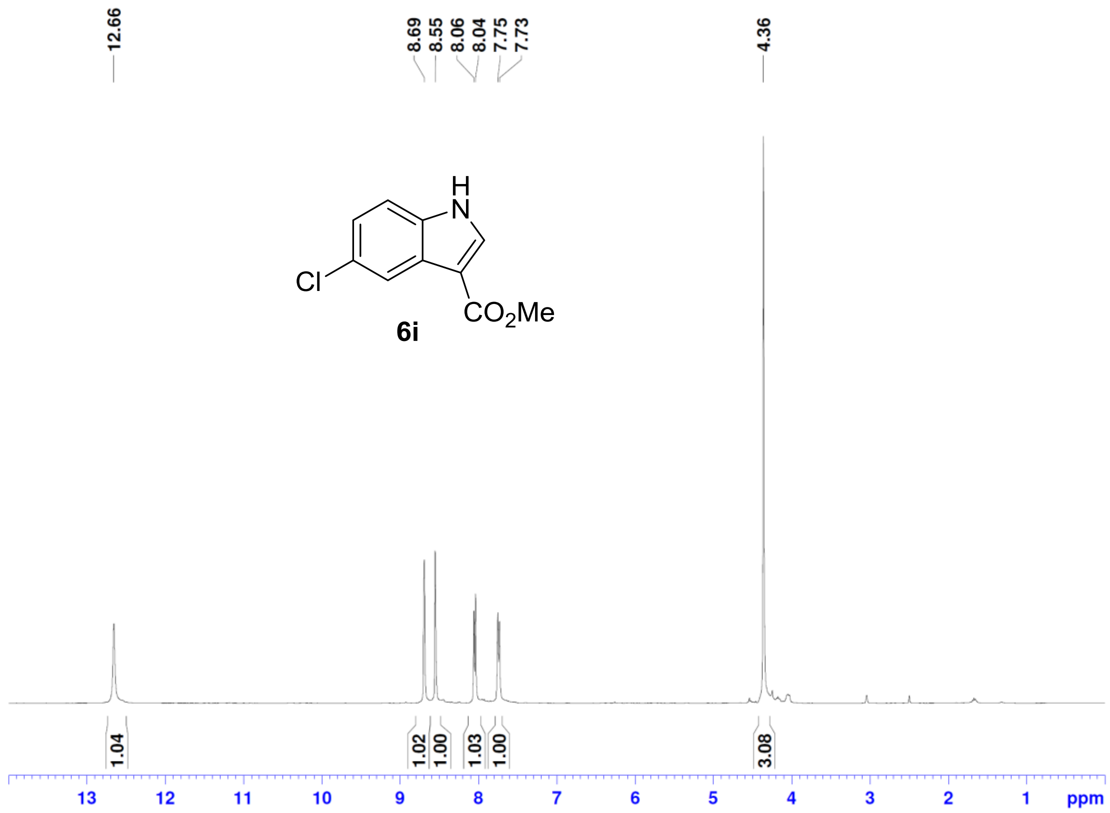
12.66
8.69
8.55
8.06
8.04
7.75
7.73
4.36
Cl
H
N
CO2Me
6i
1.04
1.02
1.00
1.03
1.00
3.08
13 12 11 10 9 8 7 6 5 4 3 2 1 ppm

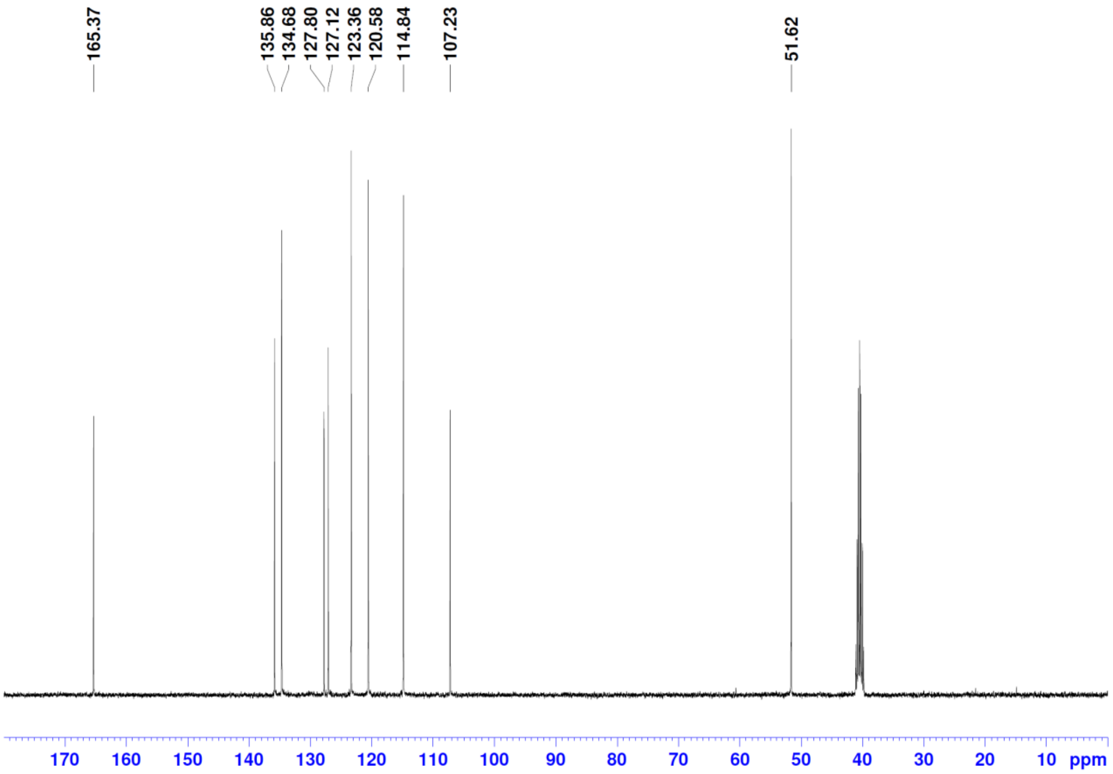
165.37
135.86
134.68
127.80
127.12
123.36
120.58
114.84
107.23
51.62
170 160 150 140 130 120 110 100 90 80 70 60 50 40 30 20 10 ppm

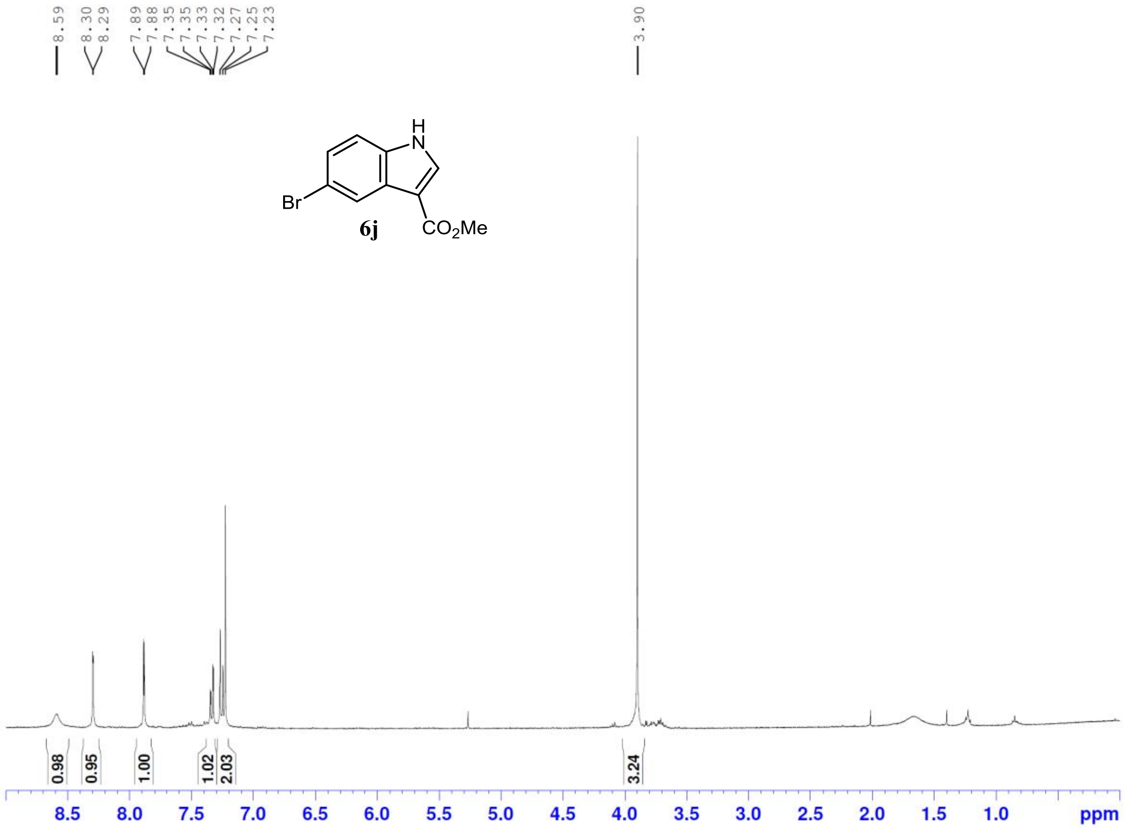

8.59
8.30
8.29
7.89
7.88
7.35
7.35
7.33
7.32
7.27
7.25
7.23
3.90
Br
6j
CO_2Me
H
N
0.98
0.95
1.00
1.02
2.03
3.24
8.5 8.0 7.5 7.0 6.5 6.0 5.5 5.0 4.5 4.0 3.5 3.0 2.5 2.0 1.5 1.0 ppm

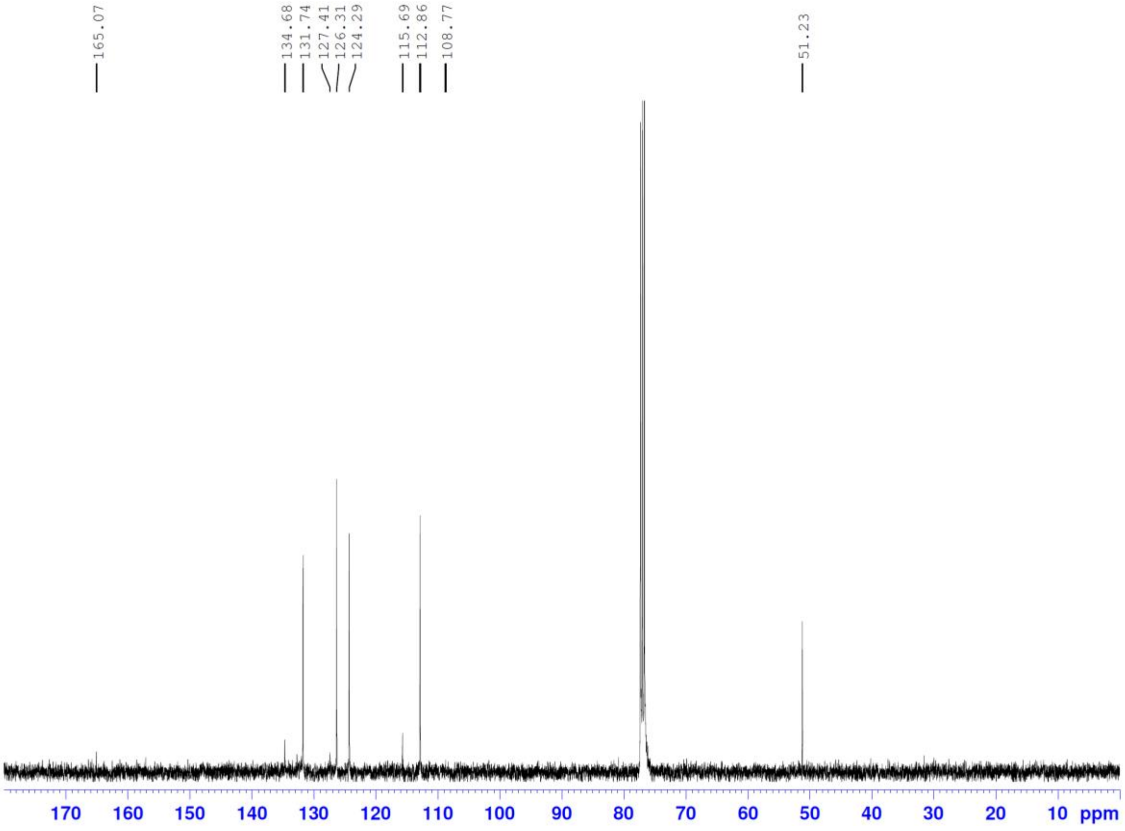

165.07
134.68
131.74
127.41
126.31
124.29
115.69
112.86
108.77
51.23
170 160 150 140 130 120 110 100 90 80 70 60 50 40 30 20 10 ppm

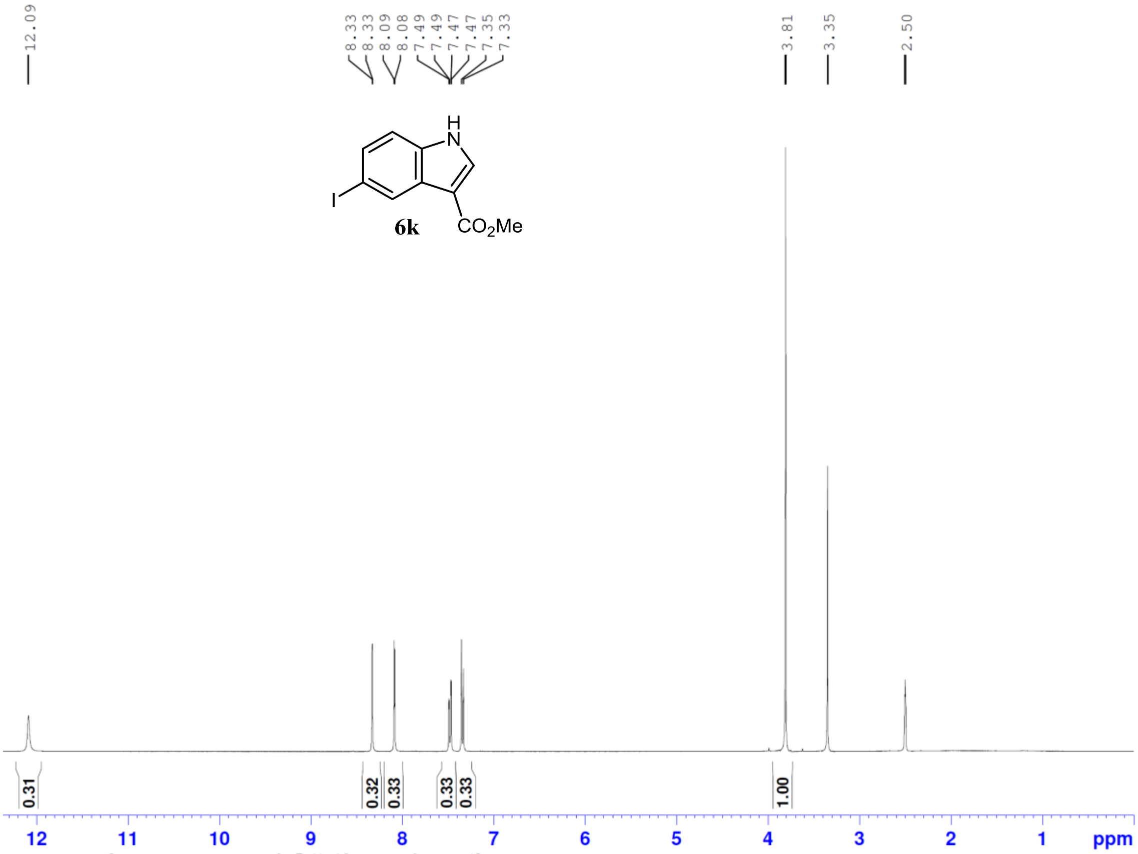
12.09
8.33
8.33
8.09
8.08
7.49
7.49
7.47
7.47
7.35
7.33
3.81
3.35
2.50
6k
CO_2Me
0.31
0.32
0.33
0.33
0.33
1.00
ppm

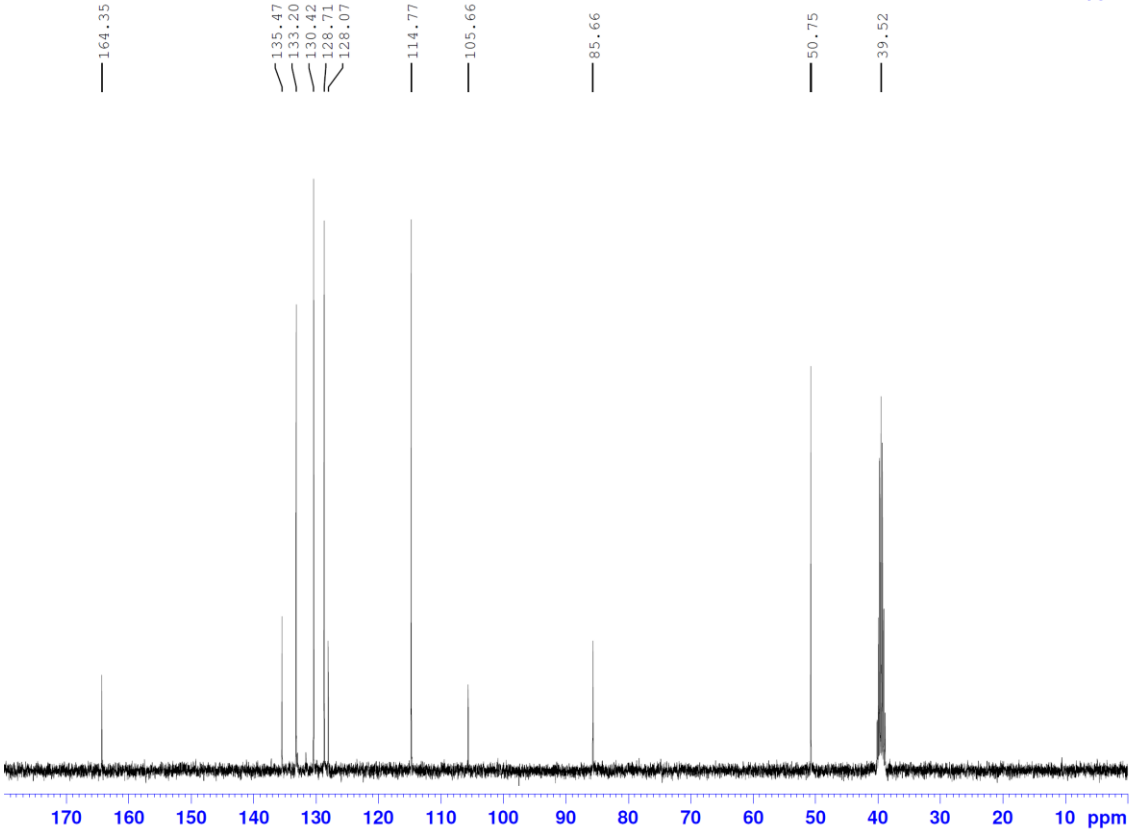
164.35
135.47
133.20
130.42
128.71
128.07
114.77
105.66
85.66
50.75
39.52
ppm

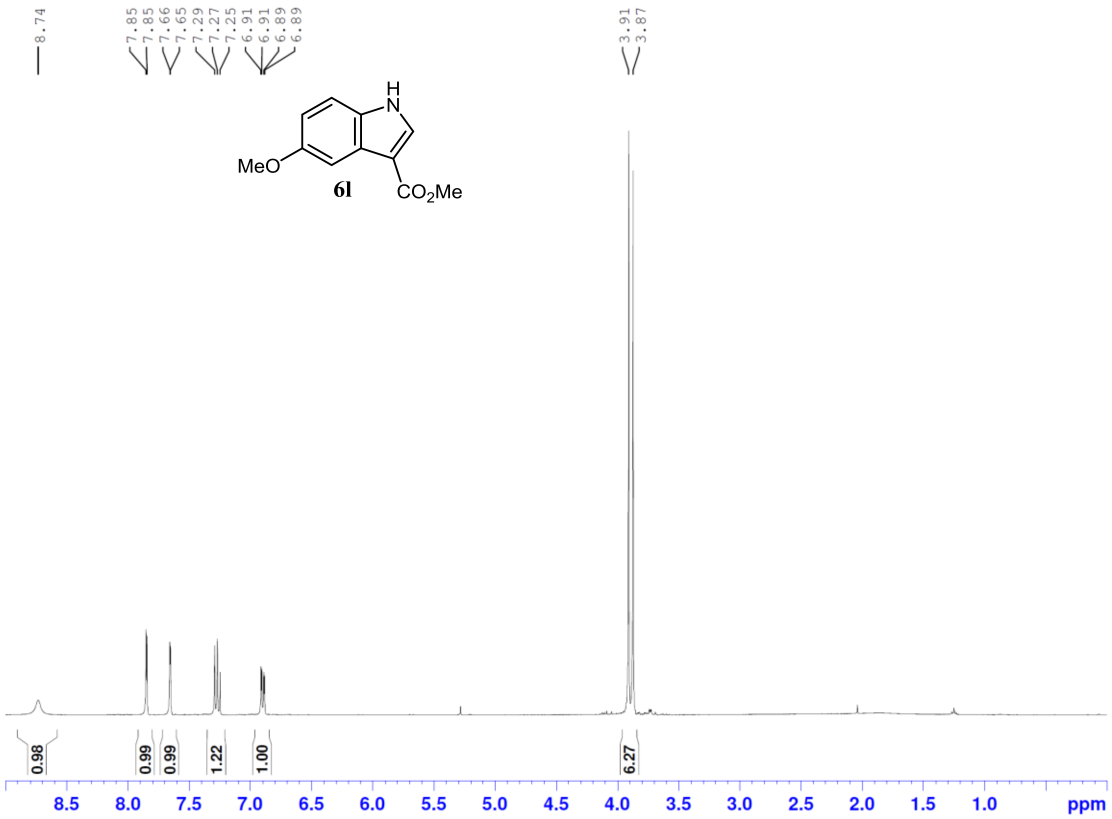
MeO
6l
CO2Me
ppm

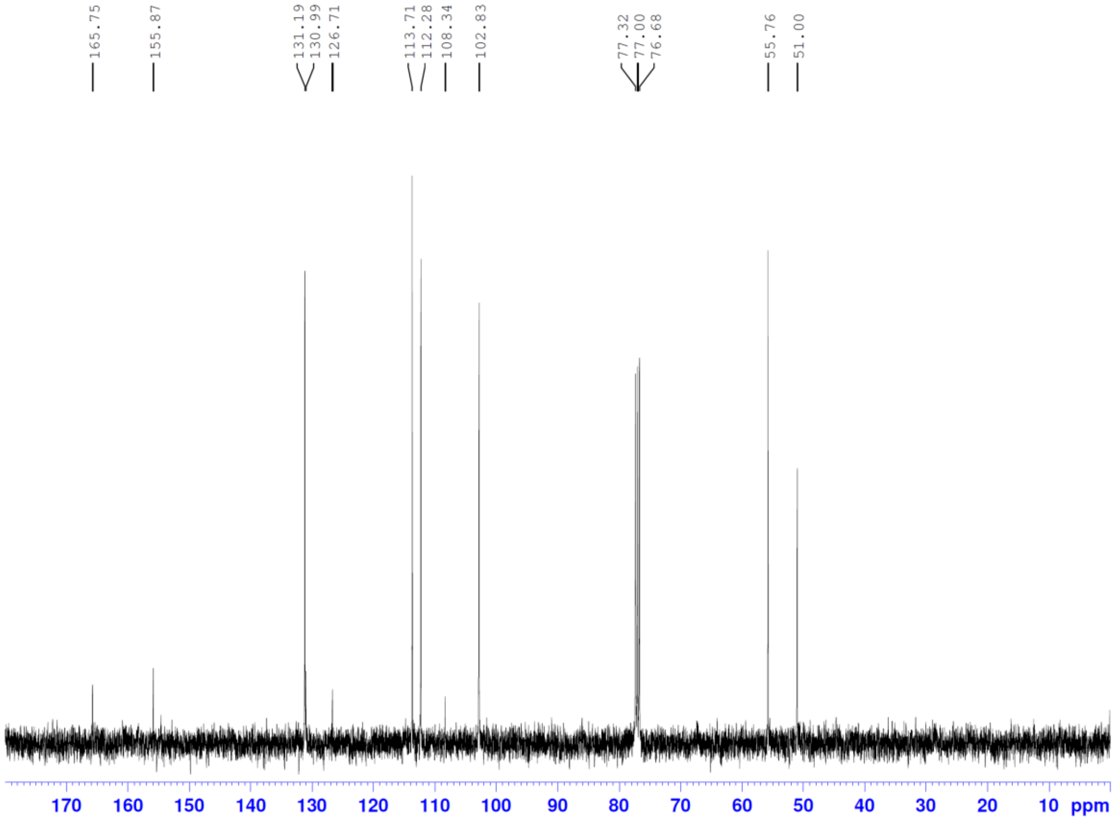
ppm

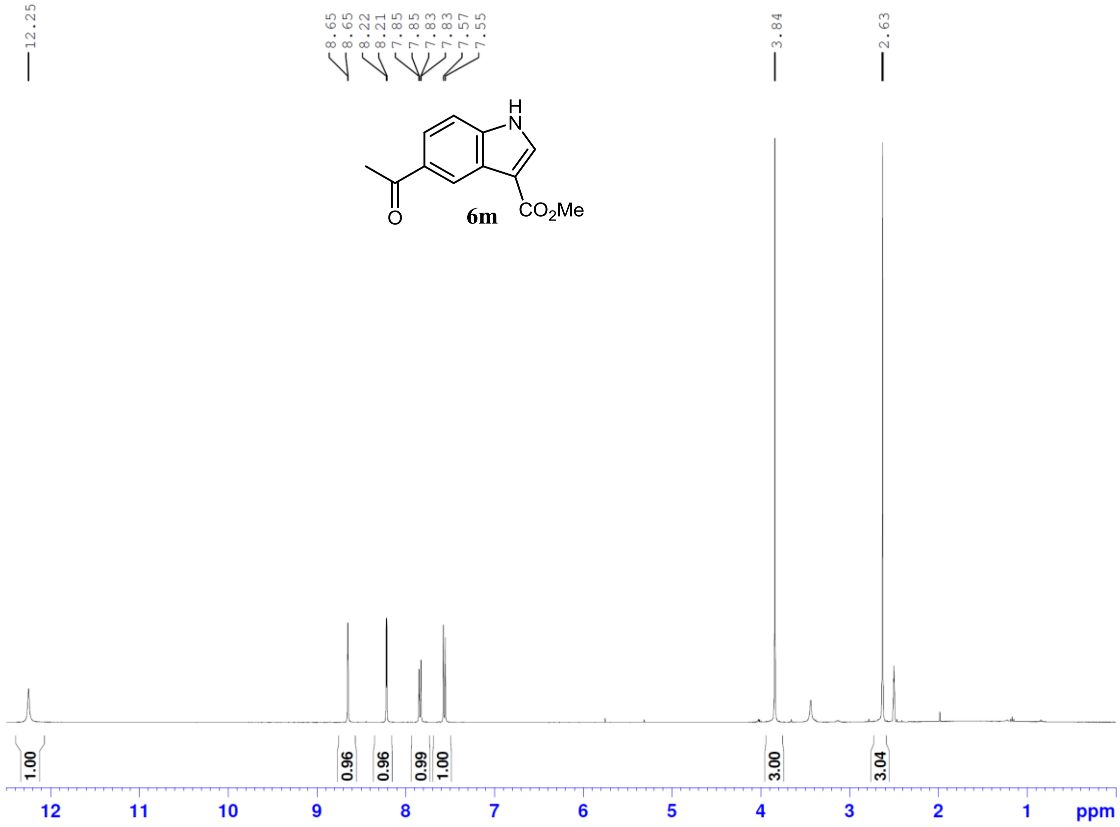
12.25
8.65
8.65
8.22
8.21
7.85
7.85
7.83
7.83
7.57
7.55
3.84
2.63
H
N
O
6m
CO_2Me
1.00
0.96
0.96
0.99
1.00
3.00
3.04
12 11 10 9 8 7 6 5 4 3 2 1 ppm

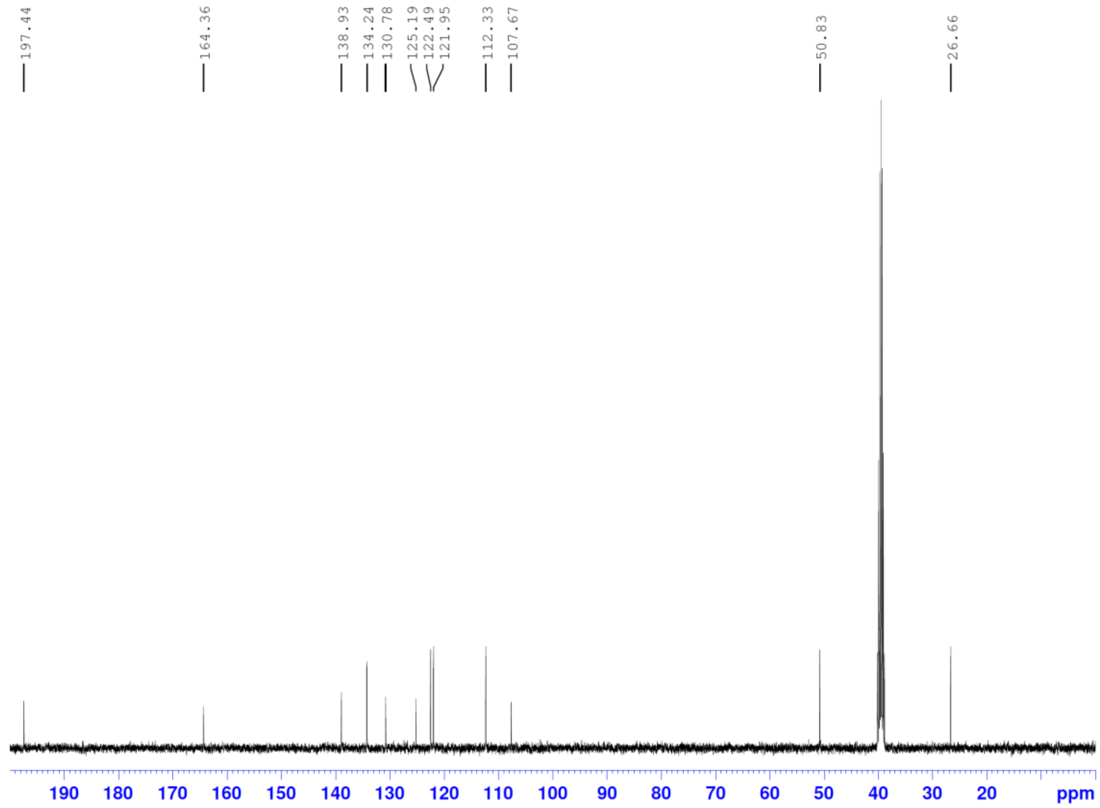
197.44
164.36
138.93
134.24
130.78
125.19
122.49
121.95
112.33
107.67
50.83
26.66
190 180 170 160 150 140 130 120 110 100 90 80 70 60 50 40 30 20 ppm

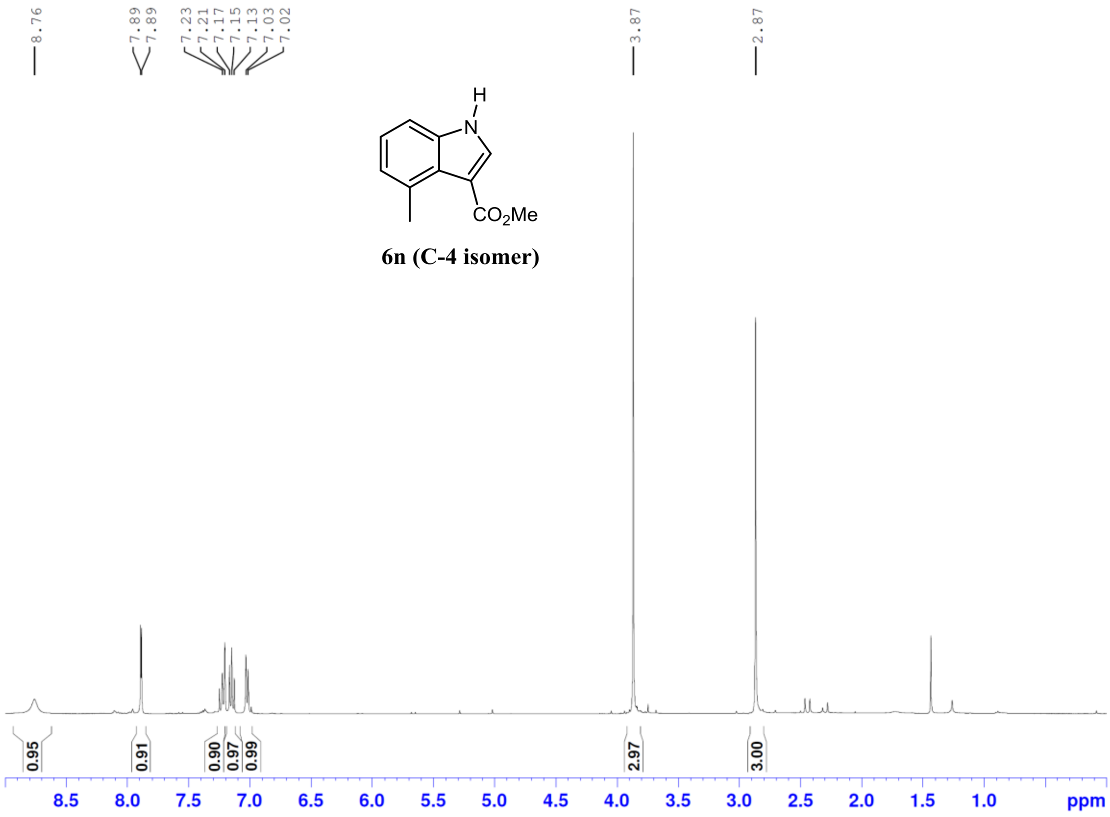
8.76
7.89
7.89
7.23
7.21
7.17
7.15
7.13
7.03
7.02
3.87
2.87
H
N
CO2Me
0.95
0.91
0.90
0.97
0.99
2.97
3.00
8.5 8.0 7.5 7.0 6.5 6.0 5.5 5.0 4.5 4.0 3.5 3.0 2.5 2.0 1.5 1.0 ppm

6n (C-4 isomer)

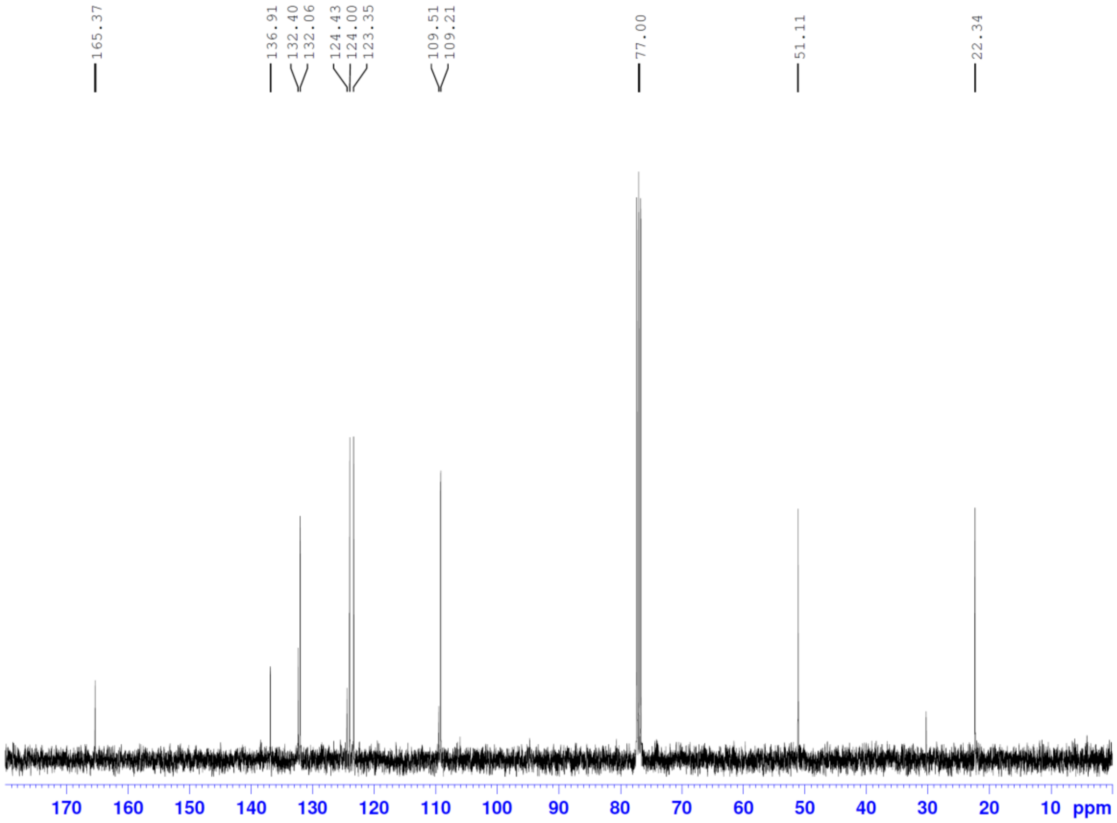
165.37
136.91
132.40
132.06
124.43
124.00
123.35
109.51
109.21
77.00
51.11
22.34
170 160 150 140 130 120 110 100 90 80 70 60 50 40 30 20 10 ppm

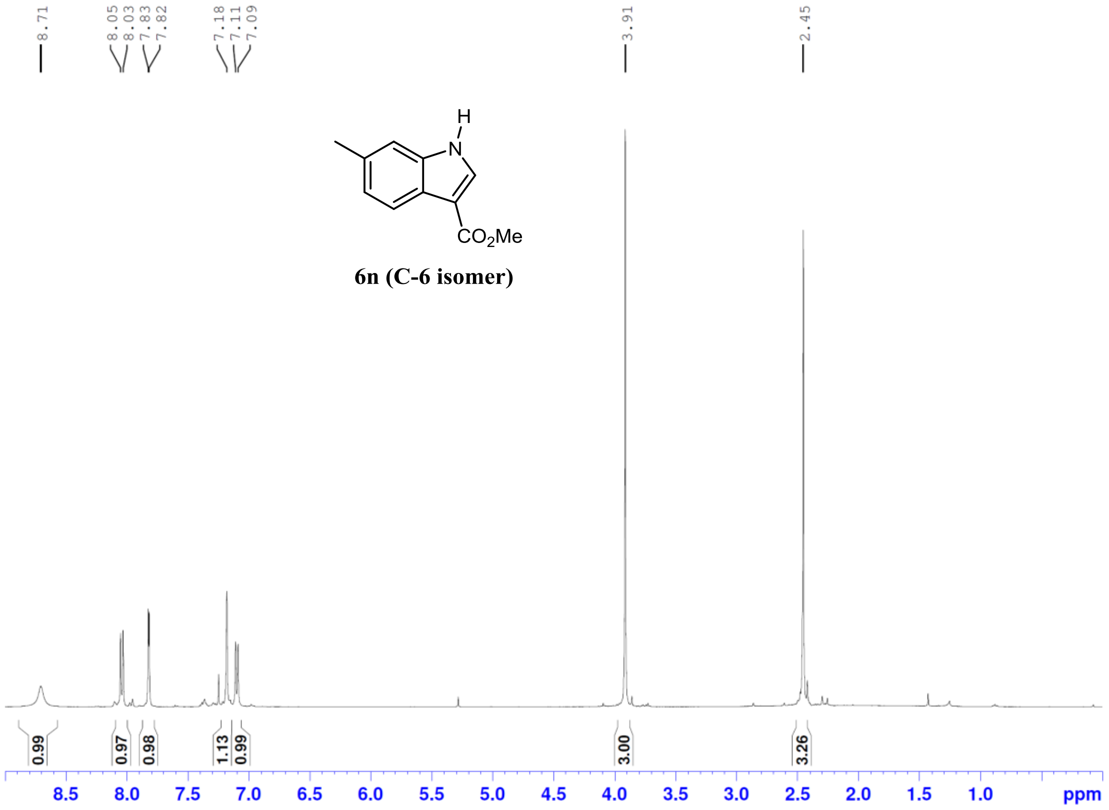
6n (C-6 isomer)
8.71
8.05
8.03
7.83
7.82
7.18
7.11
7.09
3.91
2.45
0.99
0.97
0.98
1.13
0.99
3.00
3.26
8.5 8.0 7.5 7.0 6.5 6.0 5.5 5.0 4.5 4.0 3.5 3.0 2.5 2.0 1.5 1.0 ppm

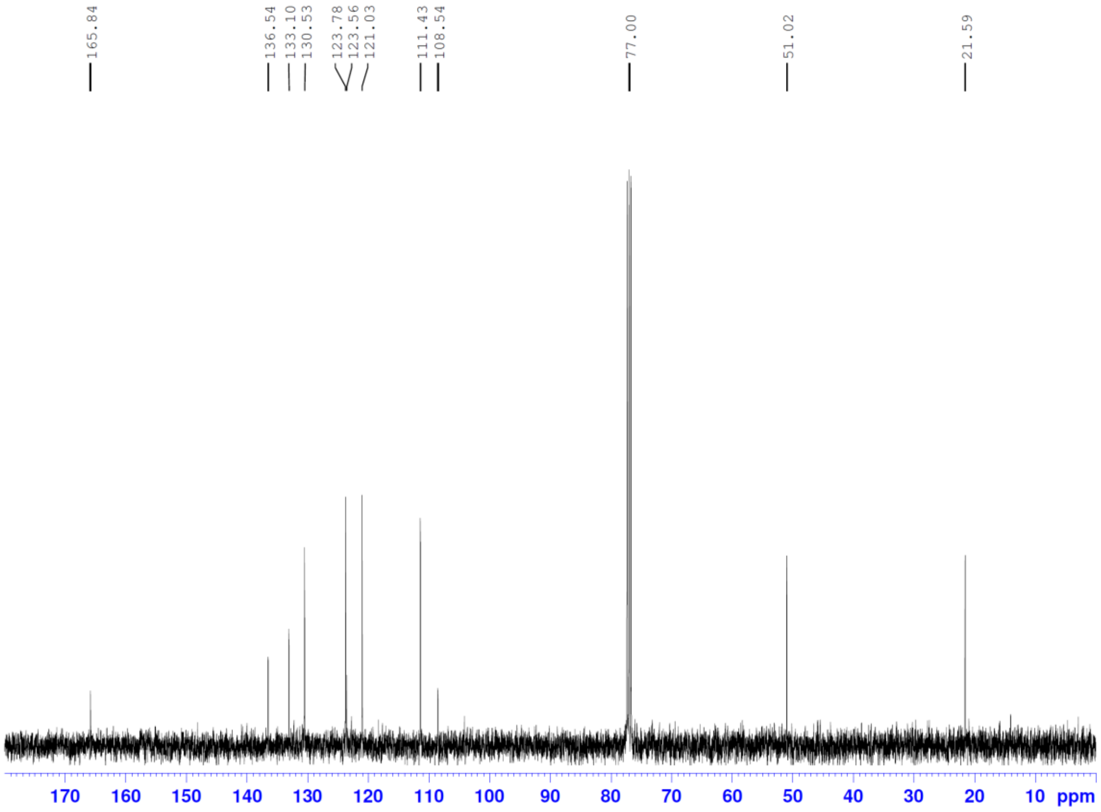
165.84
136.54
133.10
130.53
123.78
123.56
121.03
111.43
108.54
77.00
51.02
21.59
170 160 150 140 130 120 110 100 90 80 70 60 50 40 30 20 10 ppm

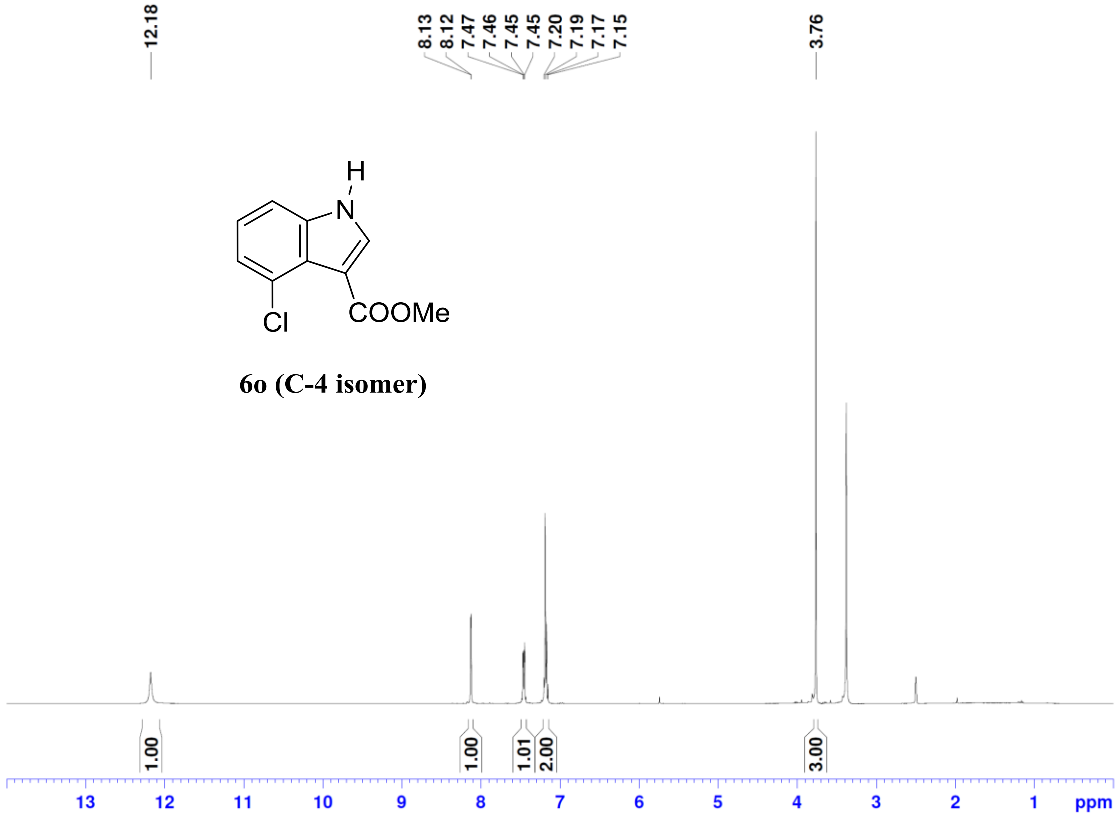

6o (C-4 isomer)

12.18
8.13
8.12
7.47
7.46
7.45
7.45
7.20
7.19
7.17
7.15
3.76
1.00
1.00
1.01
2.00
3.00
13
12
11
10
9
8
7
6
5
4
3
2
1
ppm

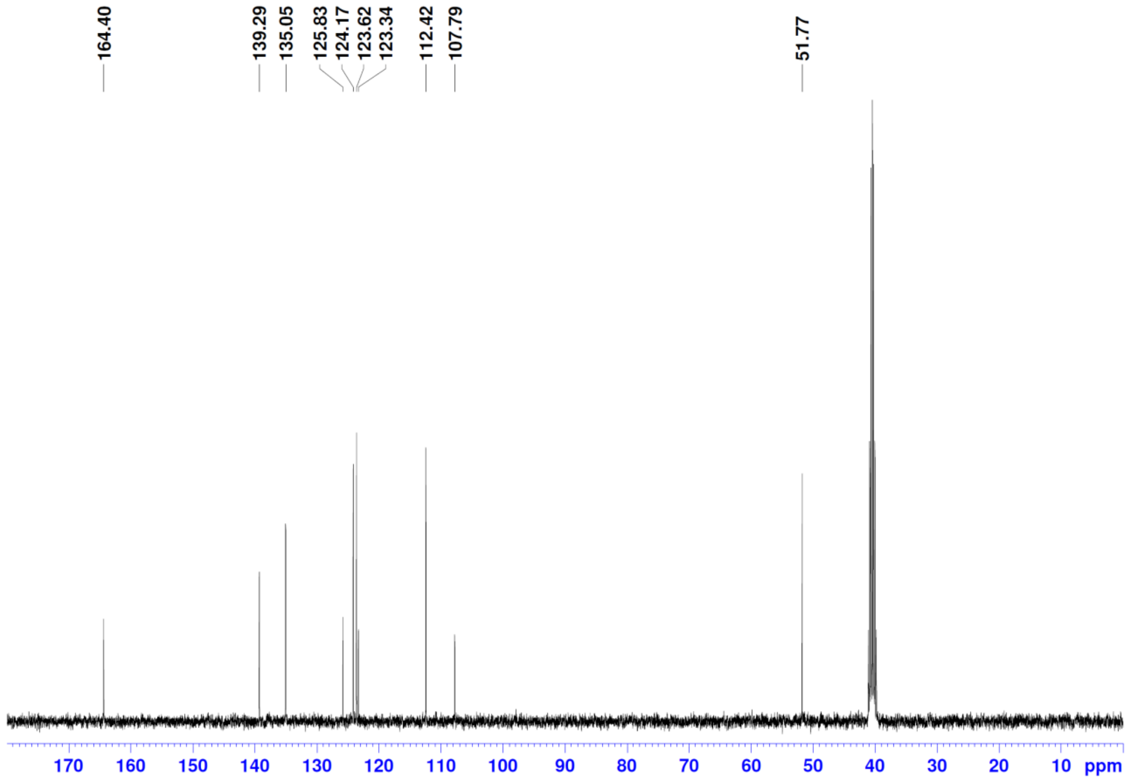

164.40
139.29
135.05
125.83
124.17
123.62
123.34
112.42
107.79
51.77
170
160
150
140
130
120
110
100
90
80
70
60
50
40
30
20
10
ppm

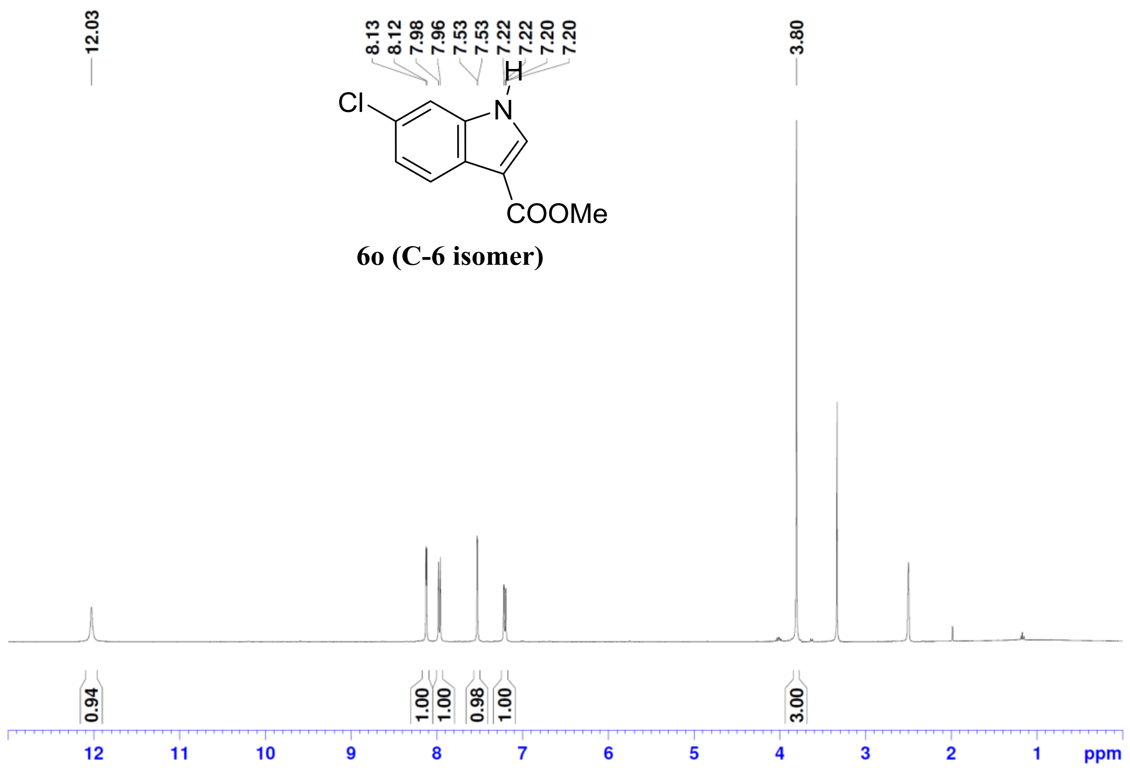
12.03
8.13
8.12
7.98
7.96
7.53
7.53
7.22
7.22
7.20
7.20
3.80
Cl
N
H
COOMe
0.94
1.00
1.00
0.98
1.00
3.00
ppm

6o (C-6 isomer)

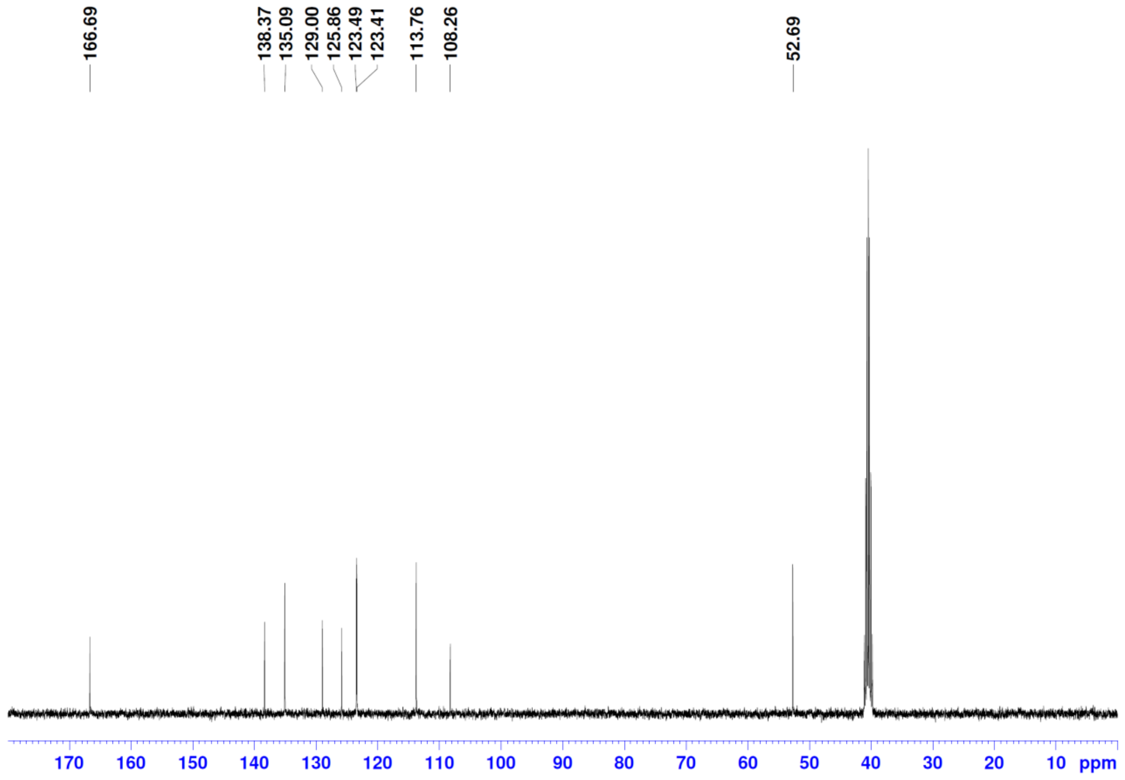
166.69
138.37
135.09
129.00
125.86
123.49
123.41
113.76
108.26
52.69
ppm

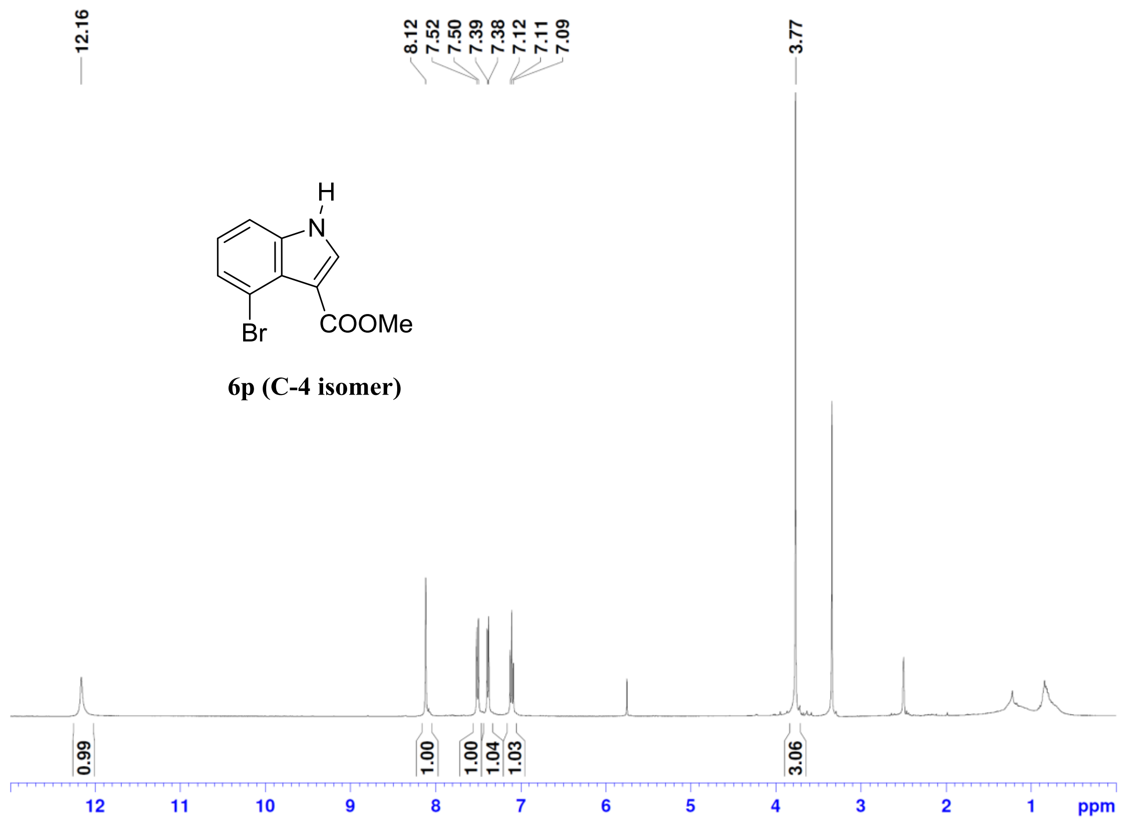
12.16
8.12
7.52
7.50
7.39
7.38
7.12
7.11
7.09
3.77
H
N
Br
COOMe
6p (C-4 isomer)
0.99
1.00
1.00
1.04
1.03
3.06
12 11 10 9 8 7 6 5 4 3 2 1 ppm

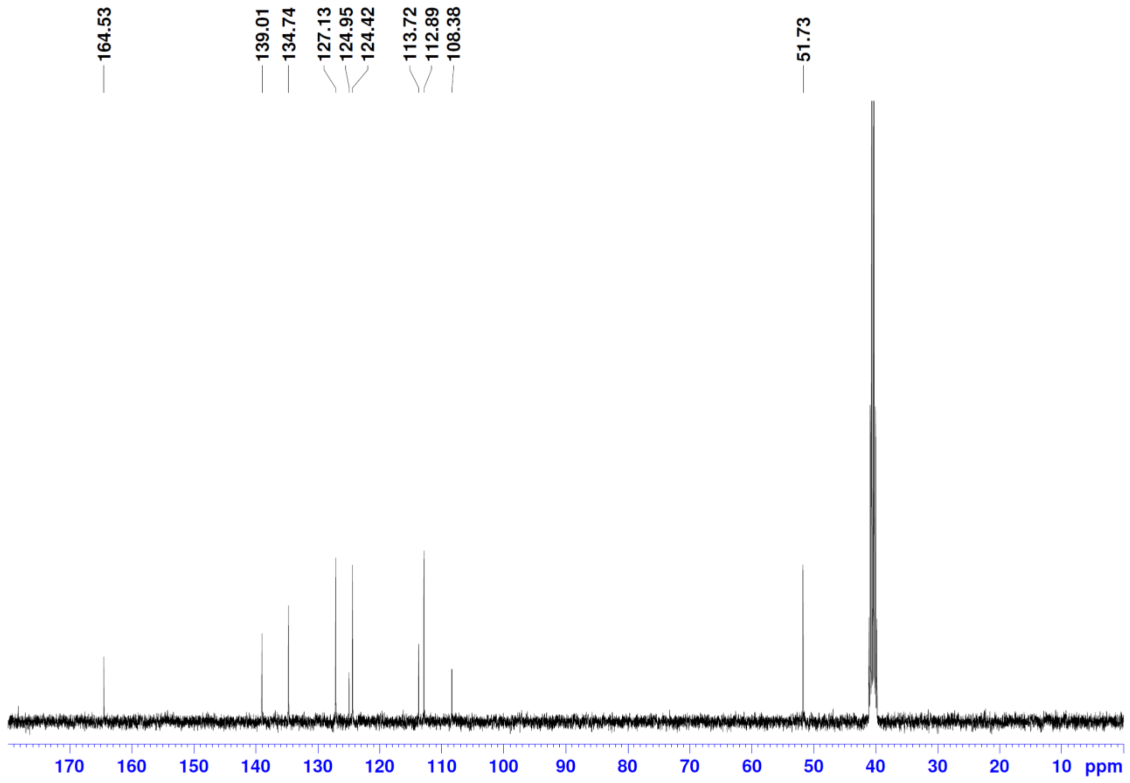
164.53
139.01
134.74
127.13
124.95
124.42
113.72
112.89
108.38
51.73
170 160 150 140 130 120 110 100 90 80 70 60 50 40 30 20 10 ppm

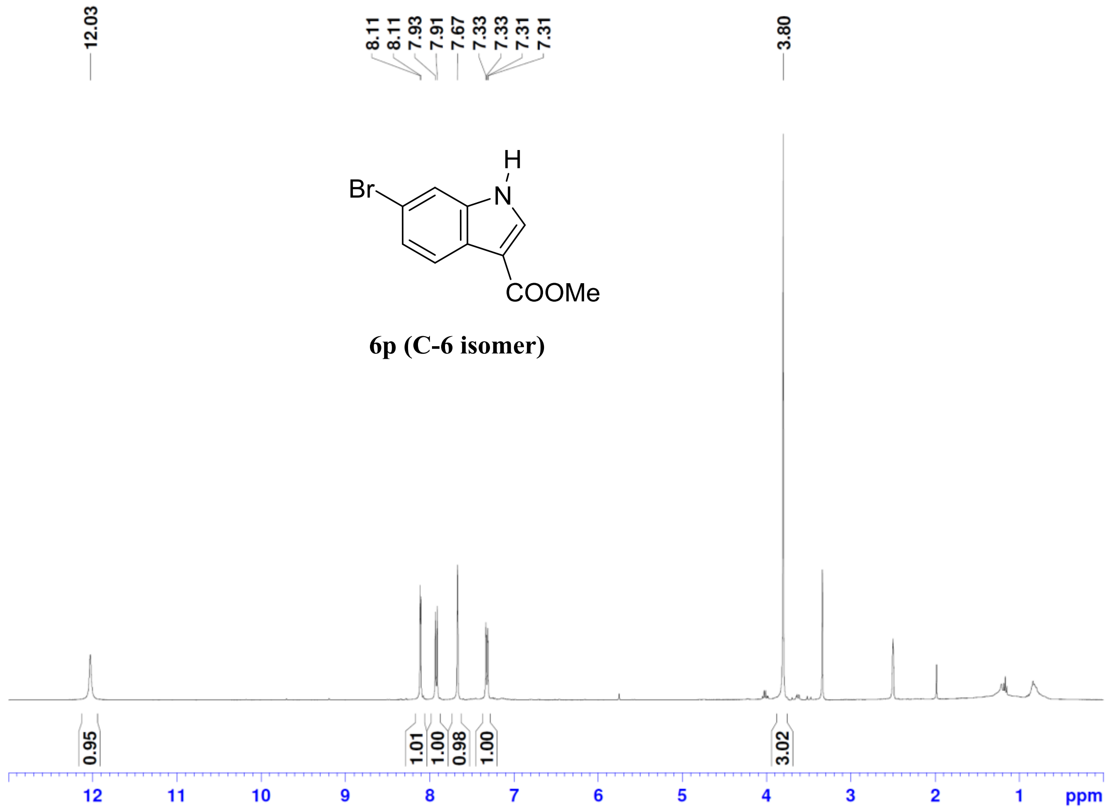
12.03
8.11
8.11
7.93
7.91
7.67
7.33
7.33
7.31
7.31
3.80
Br
H
N
COOMe
6p (C-6 isomer)
0.95
1.01
1.00
0.98
1.00
3.02
12 11 10 9 8 7 6 5 4 3 2 1 ppm

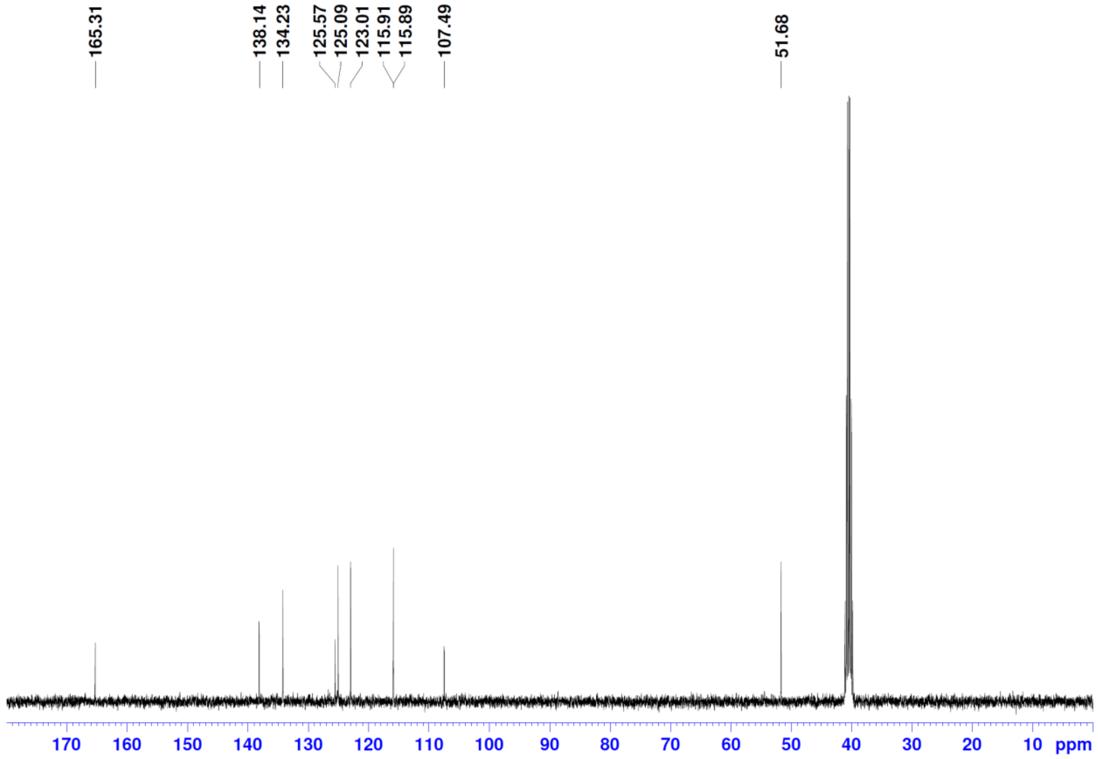
165.31
138.14
134.23
125.57
125.09
123.01
115.91
115.89
107.49
51.68
170 160 150 140 130 120 110 100 90 80 70 60 50 40 30 20 10 ppm

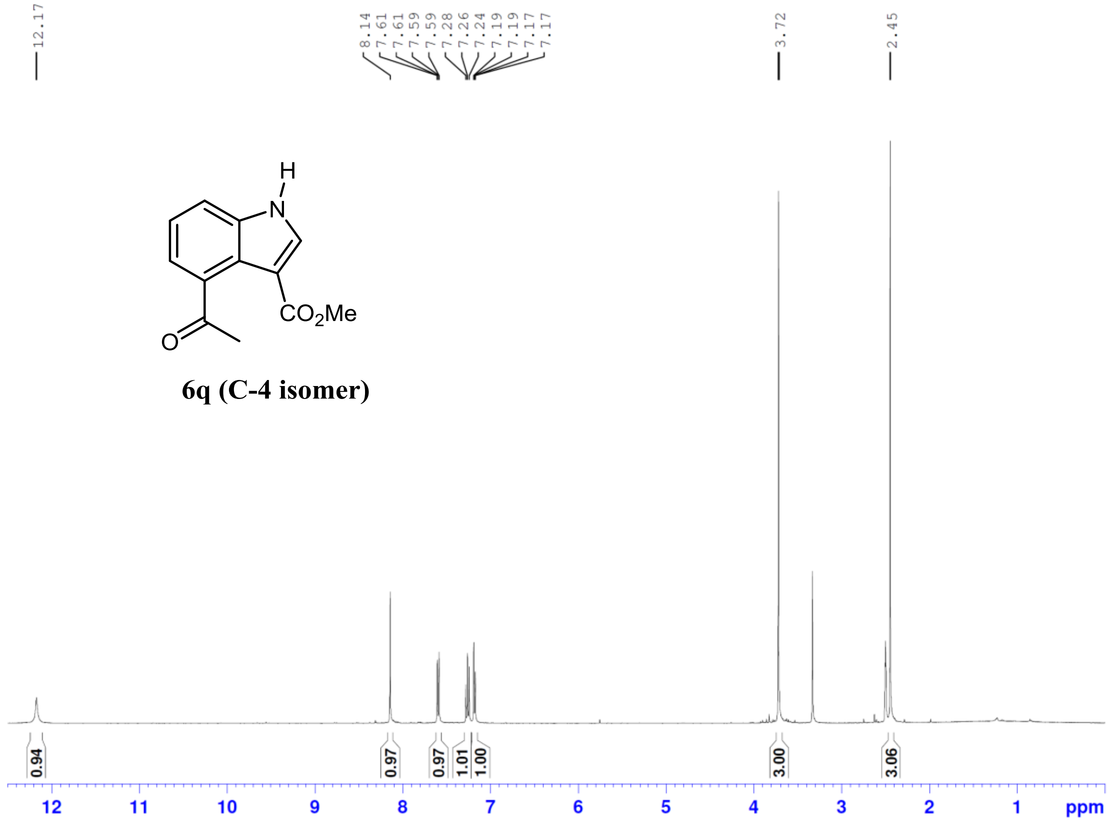
12.17
8.14
7.61
7.61
7.59
7.59
7.28
7.26
7.24
7.19
7.19
7.17
7.17
3.72
2.45
H
N
CO2Me
O
0.94
0.97
0.97
1.01
1.00
3.00
3.06
12 11 10 9 8 7 6 5 4 3 2 1 ppm

6q (C-4 isomer)

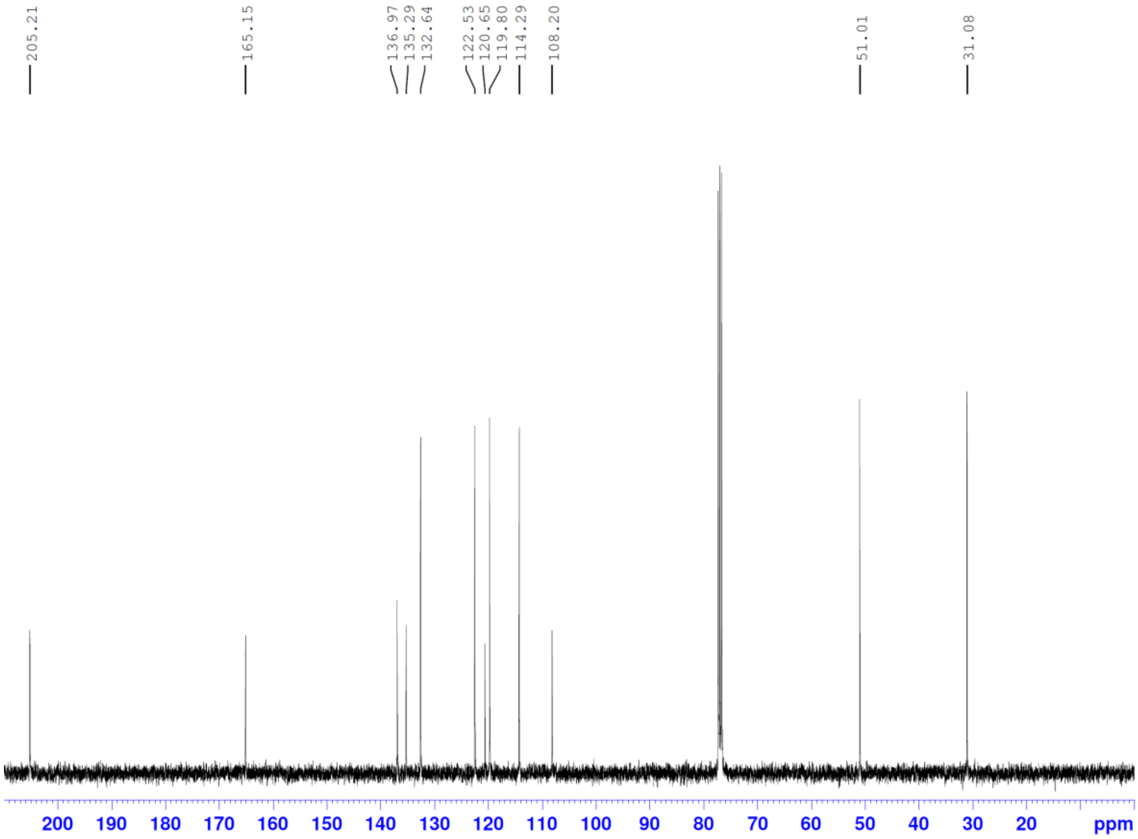
205.21
165.15
136.97
135.29
132.64
122.53
120.65
119.80
114.29
108.20
51.01
31.08
200 190 180 170 160 150 140 130 120 110 100 90 80 70 60 50 40 30 20 ppm

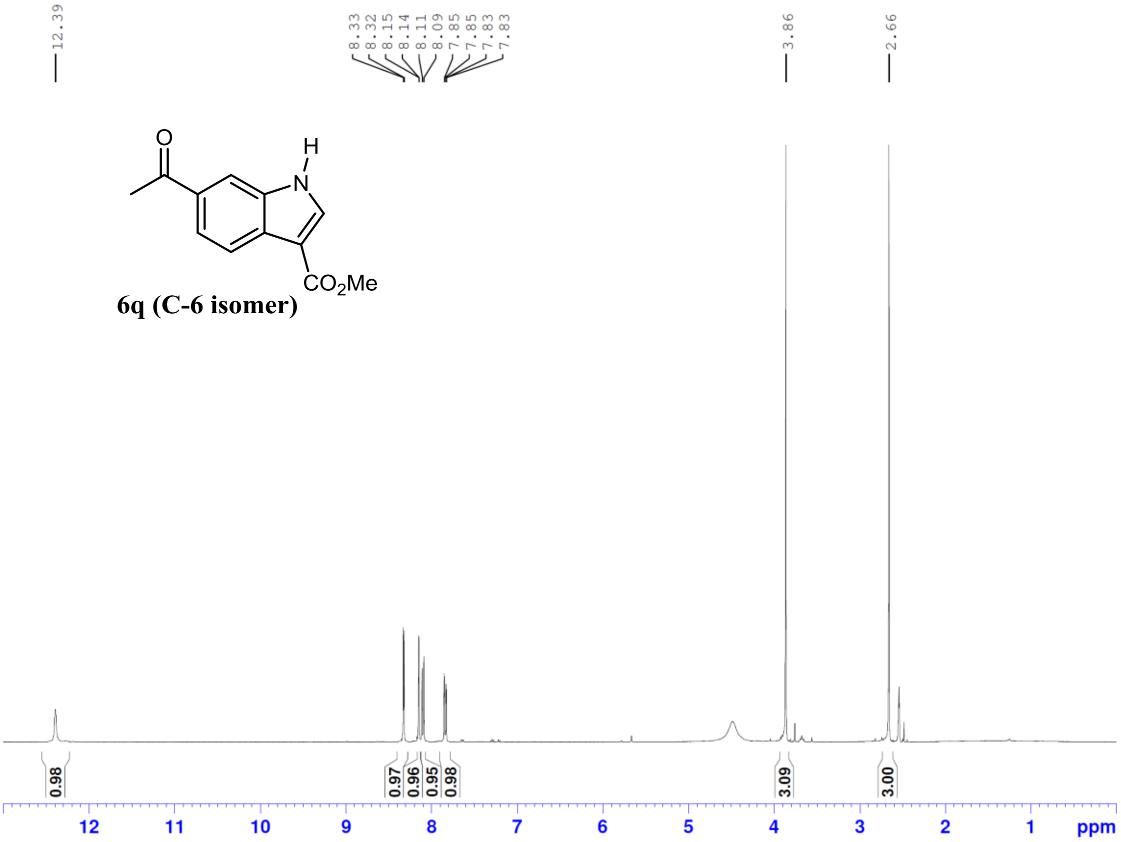
12.39
8.33
8.32
8.15
8.14
8.11
8.09
7.85
7.85
7.83
7.83
3.86
2.66
O
H
N
CO2Me
0.98
0.97
0.96
0.95
0.98
3.09
3.00
12 11 10 9 8 7 6 5 4 3 2 1 ppm

6q (C-6 isomer)

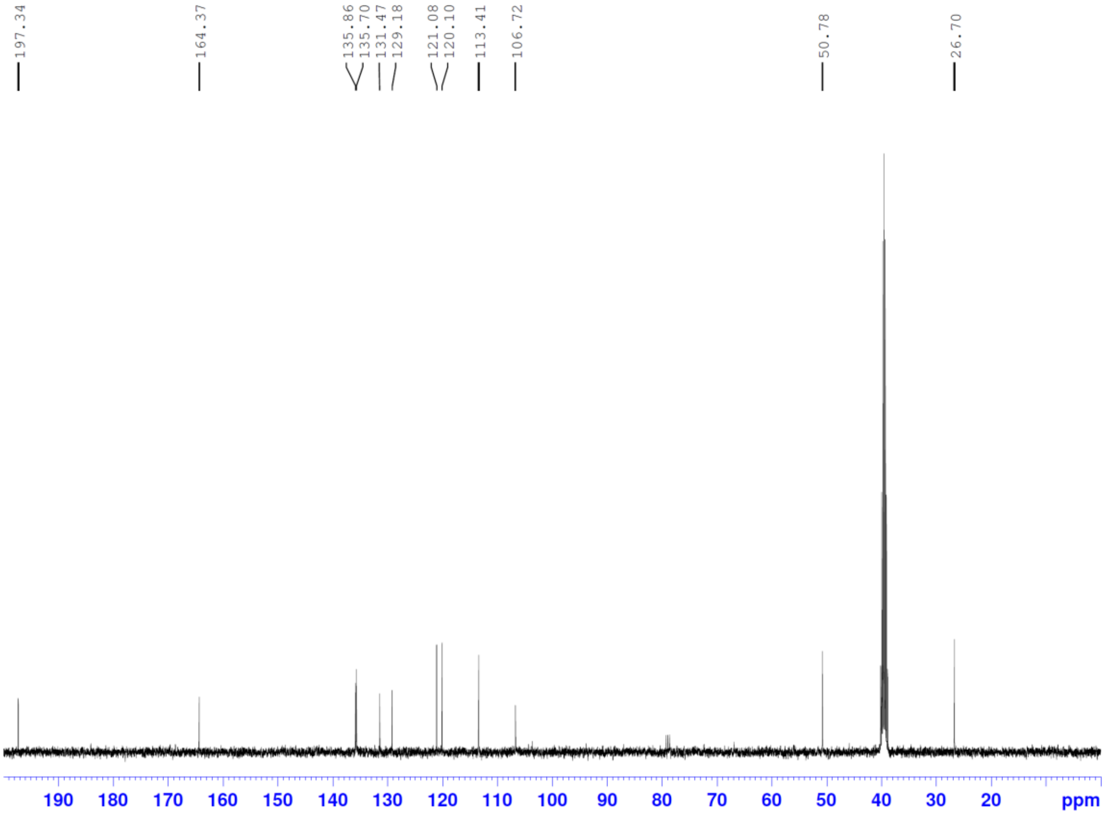
197.34
164.37
135.86
135.70
131.47
129.18
121.08
120.10
113.41
106.72
50.78
26.70
190 180 170 160 150 140 130 120 110 100 90 80 70 60 50 40 30 20 ppm

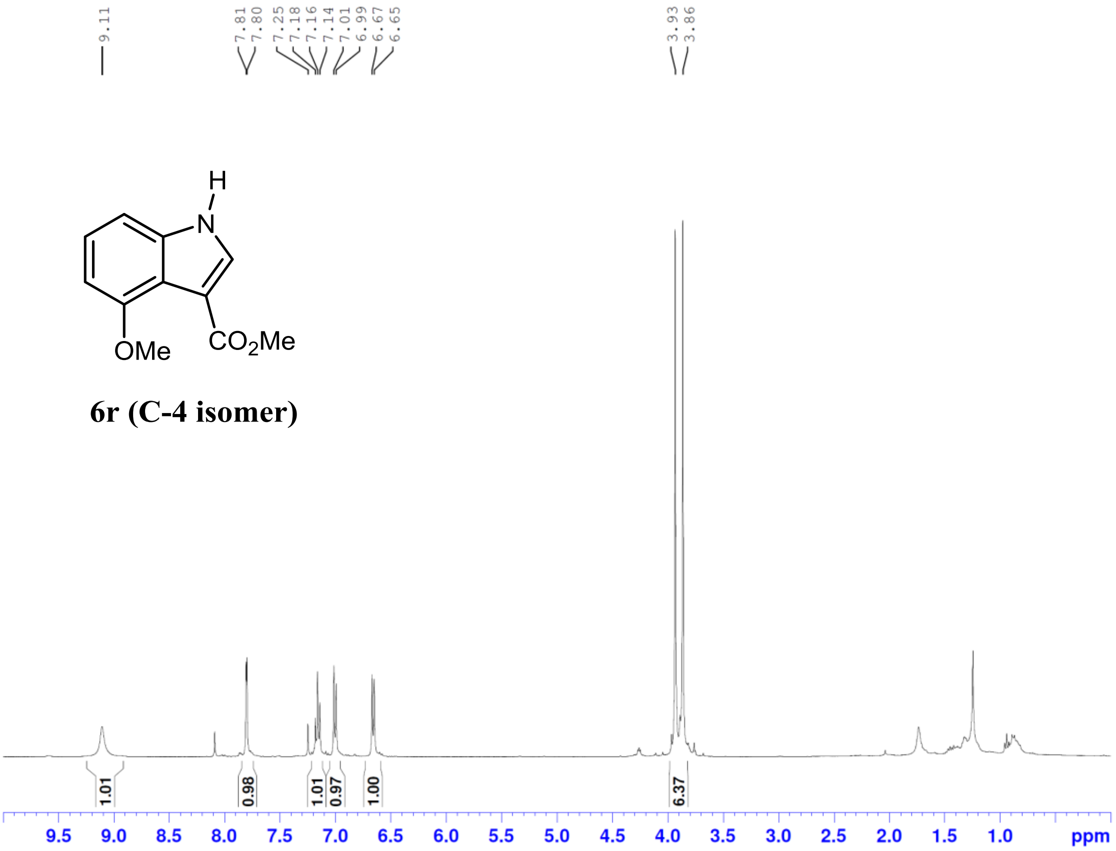
H
N
OMe
CO2Me
6r (C-4 isomer)
9.11
7.81
7.80
7.25
7.18
7.16
7.14
7.01
6.99
6.67
6.65
3.93
3.86
1.01
0.98
1.01
0.97
1.00
6.37
9.5 9.0 8.5 8.0 7.5 7.0 6.5 6.0 5.5 5.0 4.5 4.0 3.5 3.0 2.5 2.0 1.5 1.0 ppm

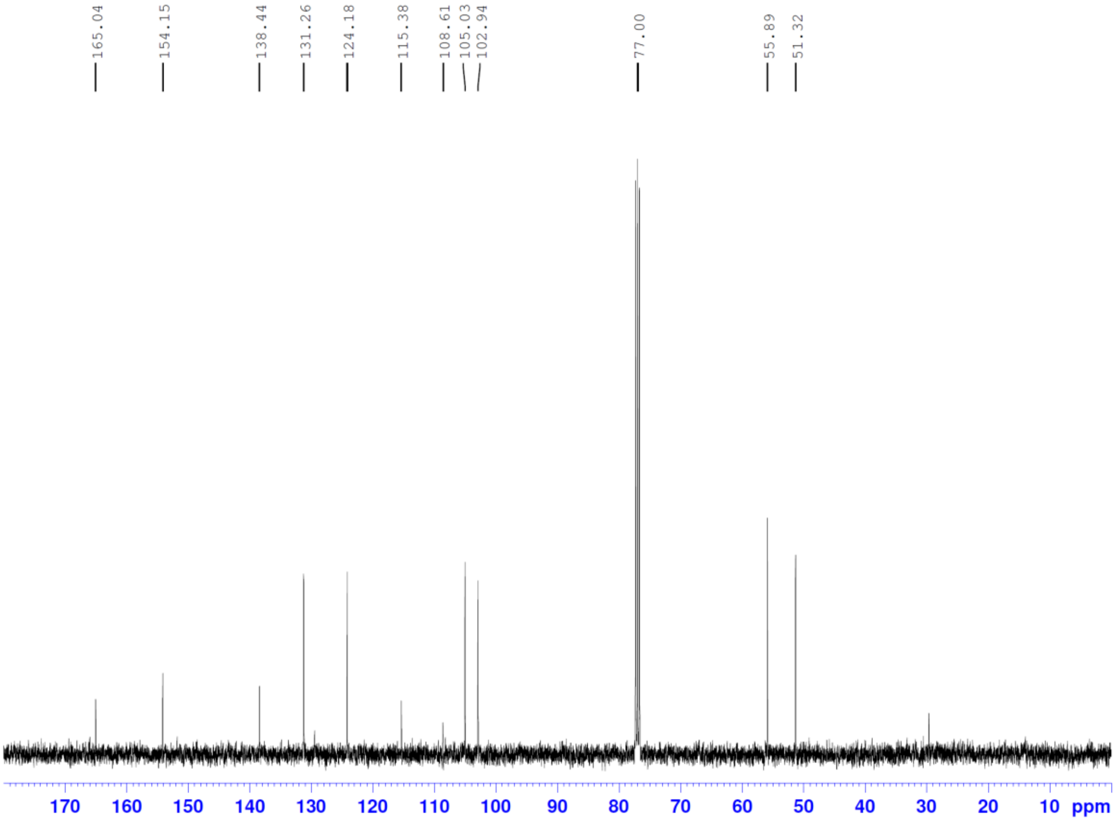
165.04
154.15
138.44
131.26
124.18
115.38
108.61
105.03
102.94
77.00
55.89
51.32
170 160 150 140 130 120 110 100 90 80 70 60 50 40 30 20 10 ppm

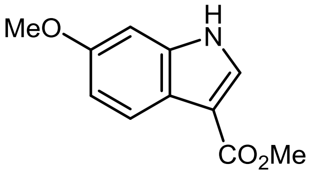
MeO
H
N
CO_2Me

6r (C-6 isomer)

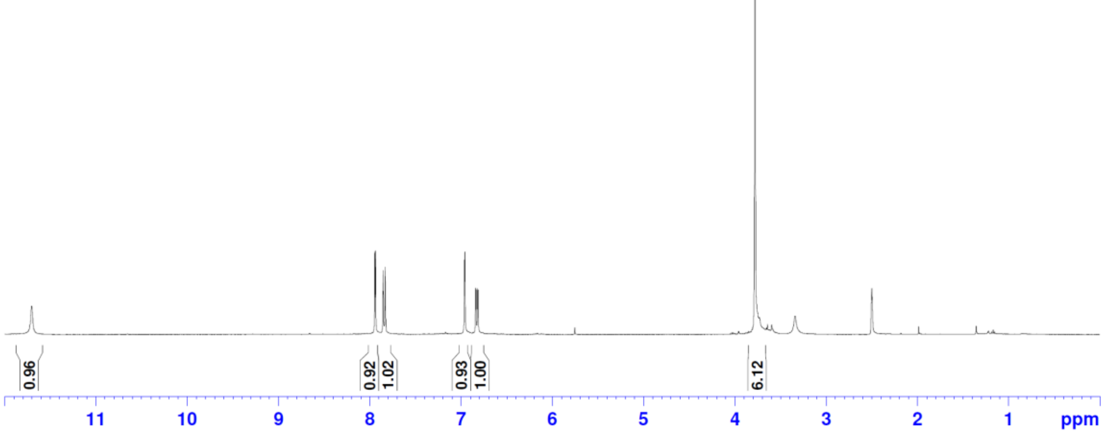
11.70
7.94
7.94
7.85
7.83
6.96
6.96
6.84
6.84
6.82
6.81
3.78
3.78
2.50
2.50
0.96
0.92
1.02
0.93
1.00
6.12
11
10
9
8
7
6
5
4
3
2
1
ppm

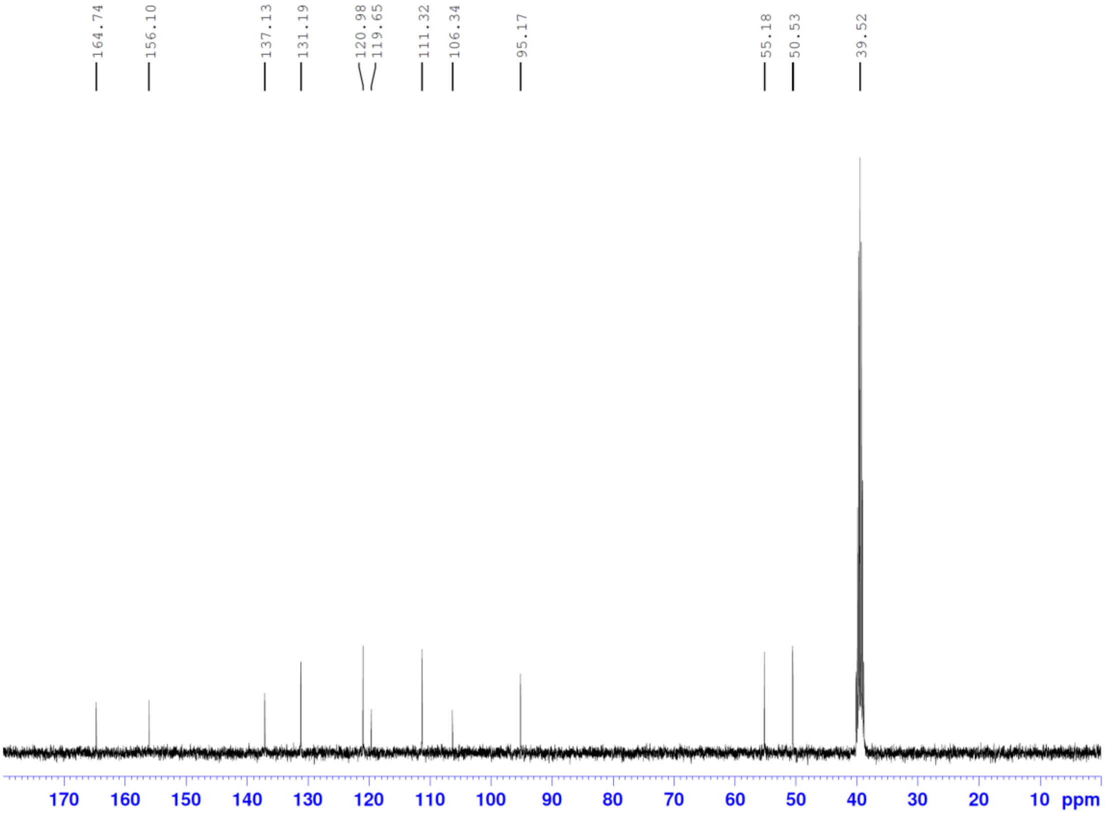
164.74
156.10
137.13
131.19
120.98
119.65
111.32
106.34
95.17
55.18
50.53
39.52
170
160
150
140
130
120
110
100
90
80
70
60
50
40
30
20
10
ppm

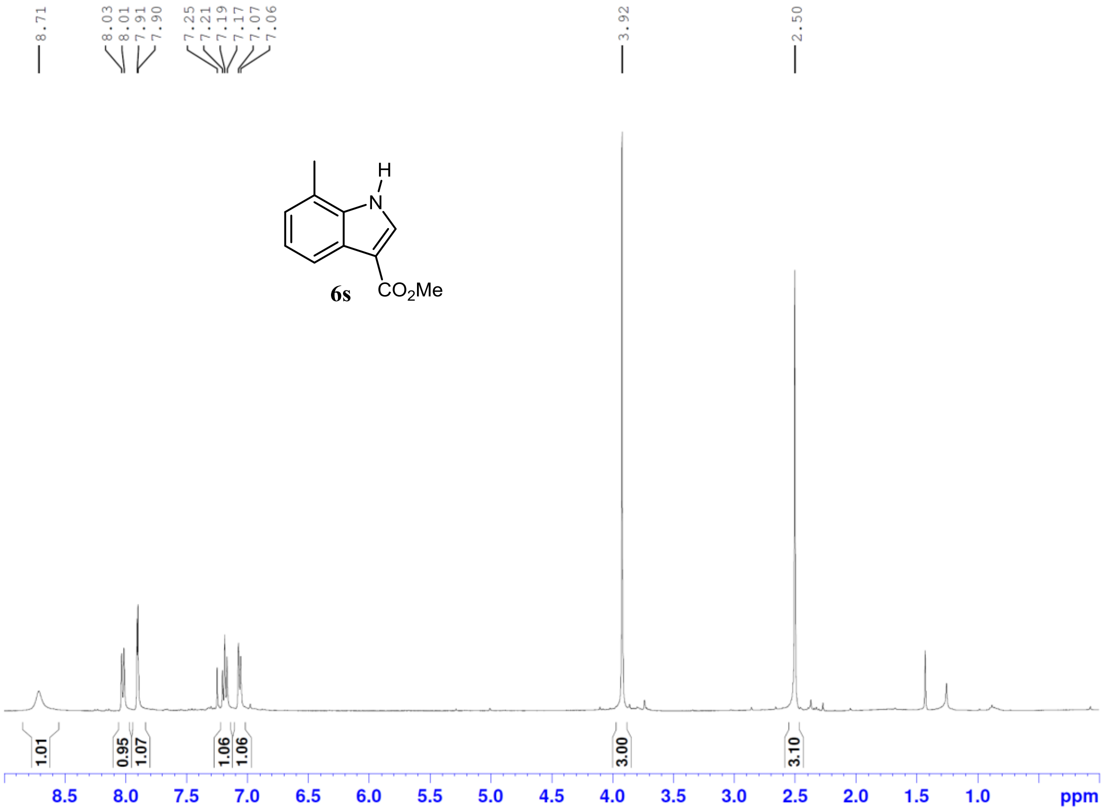
8.71
8.03
8.01
7.91
7.90
7.25
7.21
7.19
7.17
7.07
7.06
3.92
2.50
H
N
6s
CO2Me
1.01
0.95
1.07
1.06
1.06
3.00
3.10
8.5 8.0 7.5 7.0 6.5 6.0 5.5 5.0 4.5 4.0 3.5 3.0 2.5 2.0 1.5 1.0 ppm

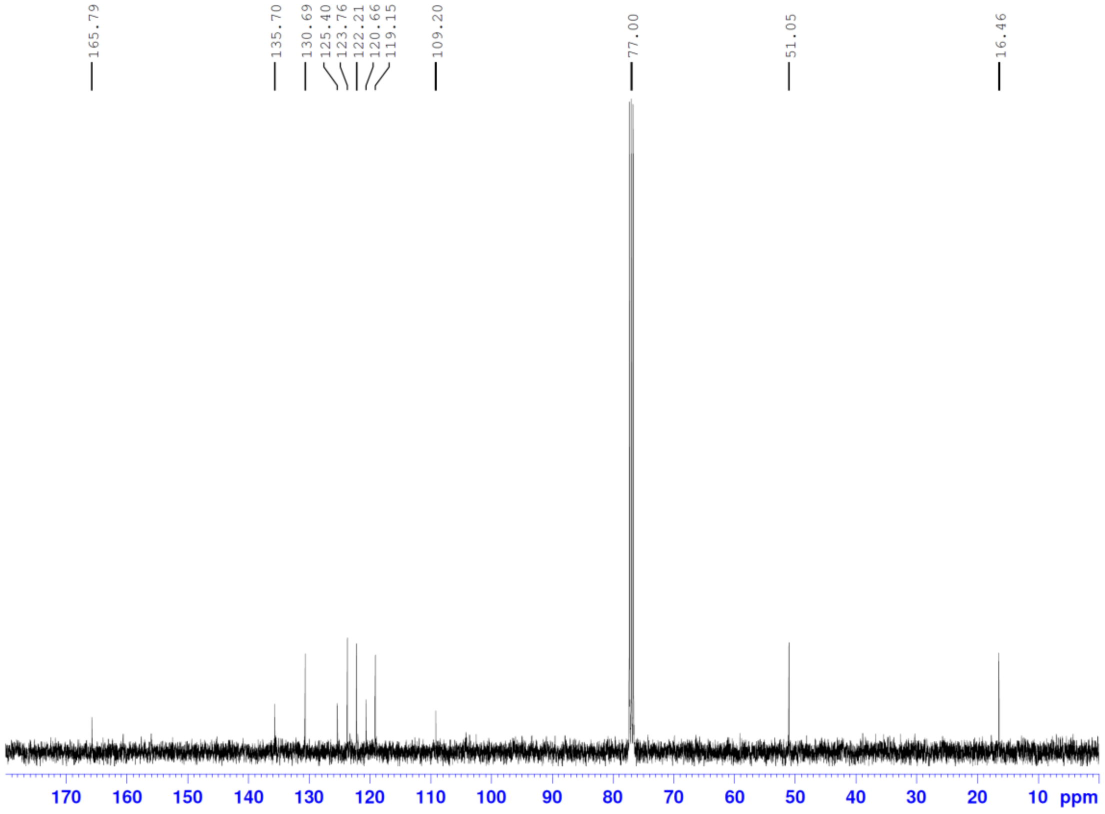
165.79
135.70
130.69
125.40
123.76
122.21
120.66
119.15
109.20
77.00
51.05
16.46
170 160 150 140 130 120 110 100 90 80 70 60 50 40 30 20 10 ppm

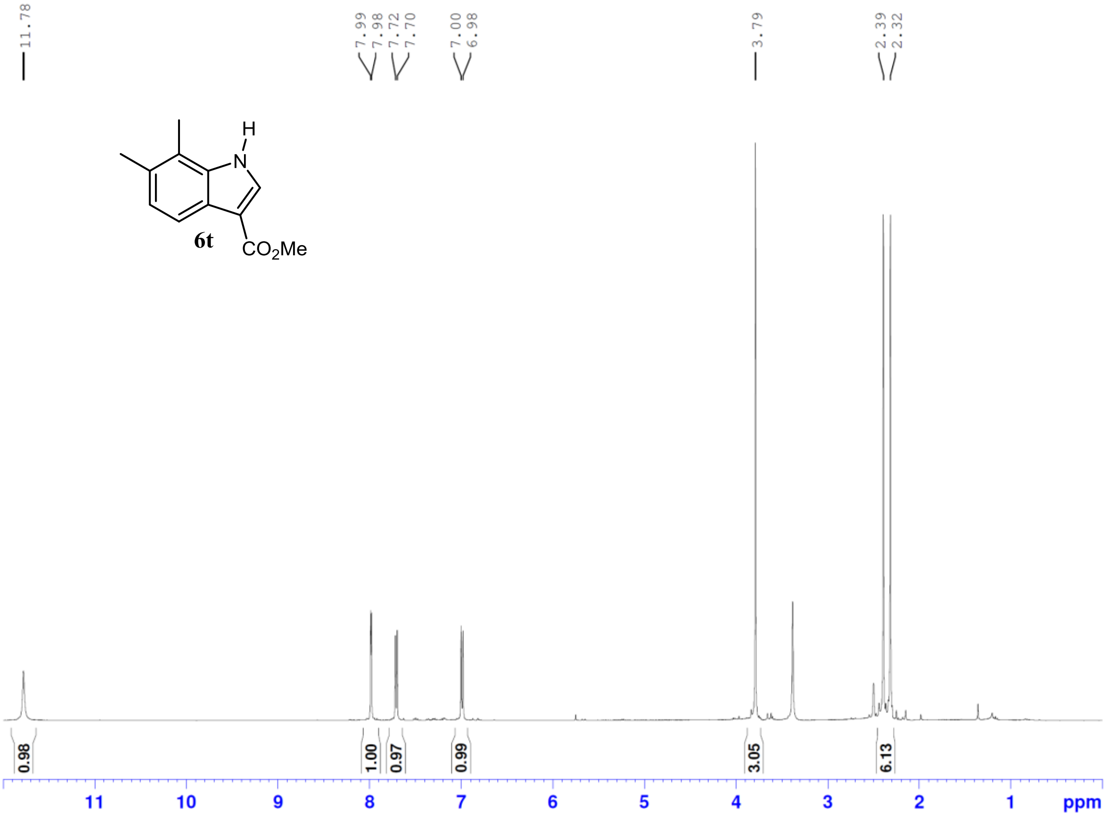
11.78
7.99
7.98
7.72
7.70
7.00
6.98
3.79
2.39
2.32
H
N
6t
CO2Me
0.98
1.00
0.97
0.99
3.05
6.13
11 10 9 8 7 6 5 4 3 2 1 ppm

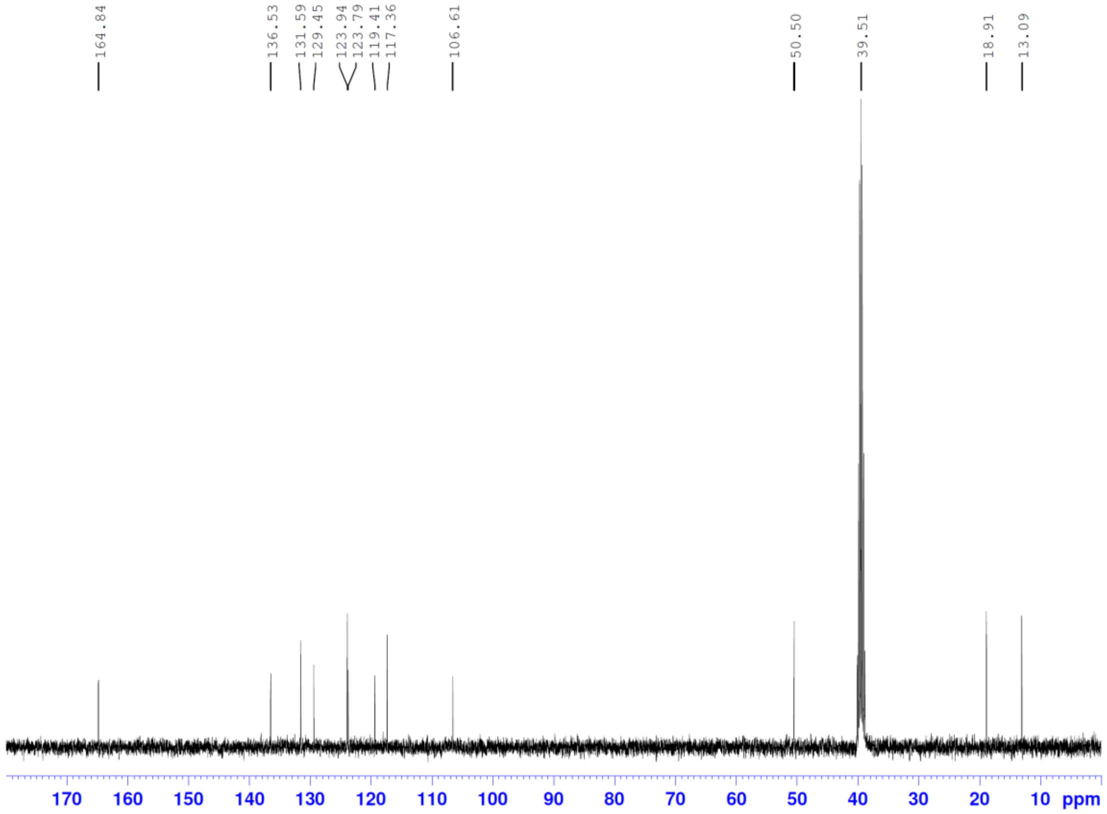
164.84
136.53
131.59
129.45
123.94
123.79
119.41
117.36
106.61
50.50
39.51
18.91
13.09
170 160 150 140 130 120 110 100 90 80 70 60 50 40 30 20 10 ppm

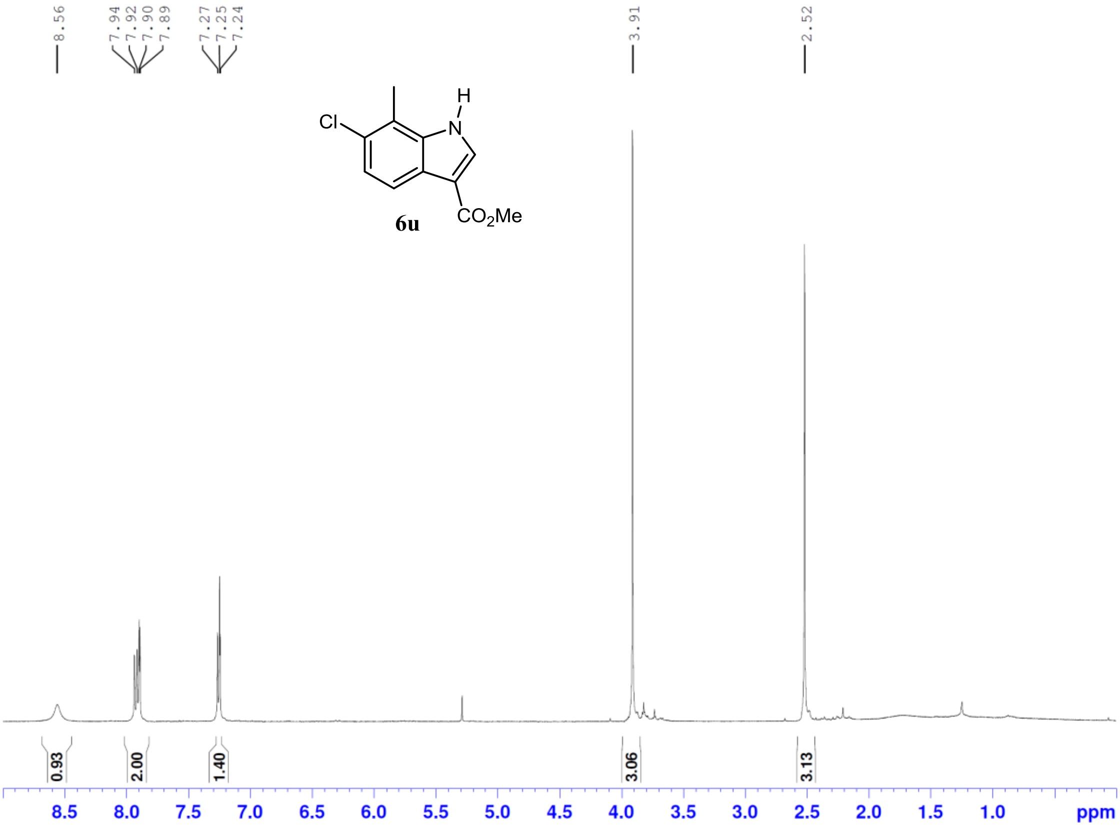
8.56
7.94
7.92
7.90
7.89
7.27
7.25
7.24
3.91
2.52
Cl
H
N
CO2Me
6u
0.93
2.00
1.40
3.06
3.13
8.5 8.0 7.5 7.0 6.5 6.0 5.5 5.0 4.5 4.0 3.5 3.0 2.5 2.0 1.5 1.0 ppm

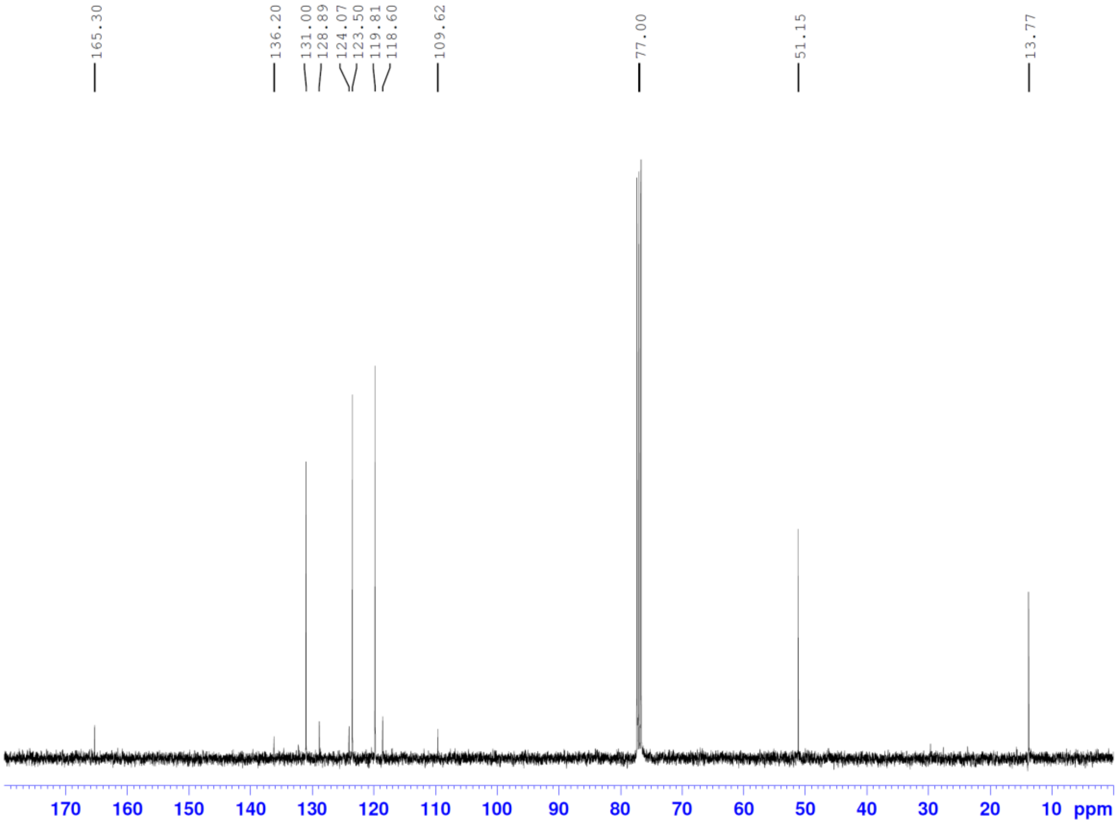
165.30
136.20
131.00
128.89
124.07
123.50
119.81
118.60
109.62
77.00
51.15
13.77
170 160 150 140 130 120 110 100 90 80 70 60 50 40 30 20 10 ppm

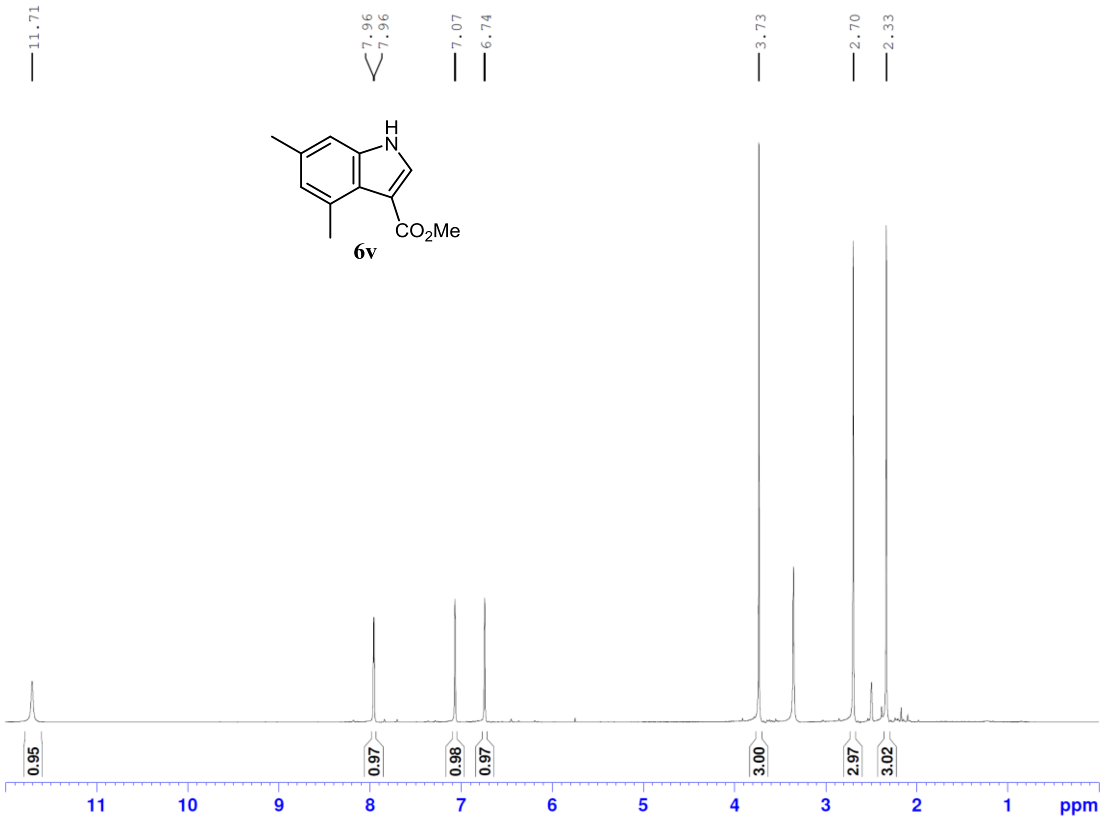
11.71
7.96
7.96
7.07
6.74
3.73
2.70
2.33
H
N
CO2Me
6v
0.95
0.97
0.98
0.97
3.00
2.97
3.02
11
10
9
8
7
6
5
4
3
2
1
ppm

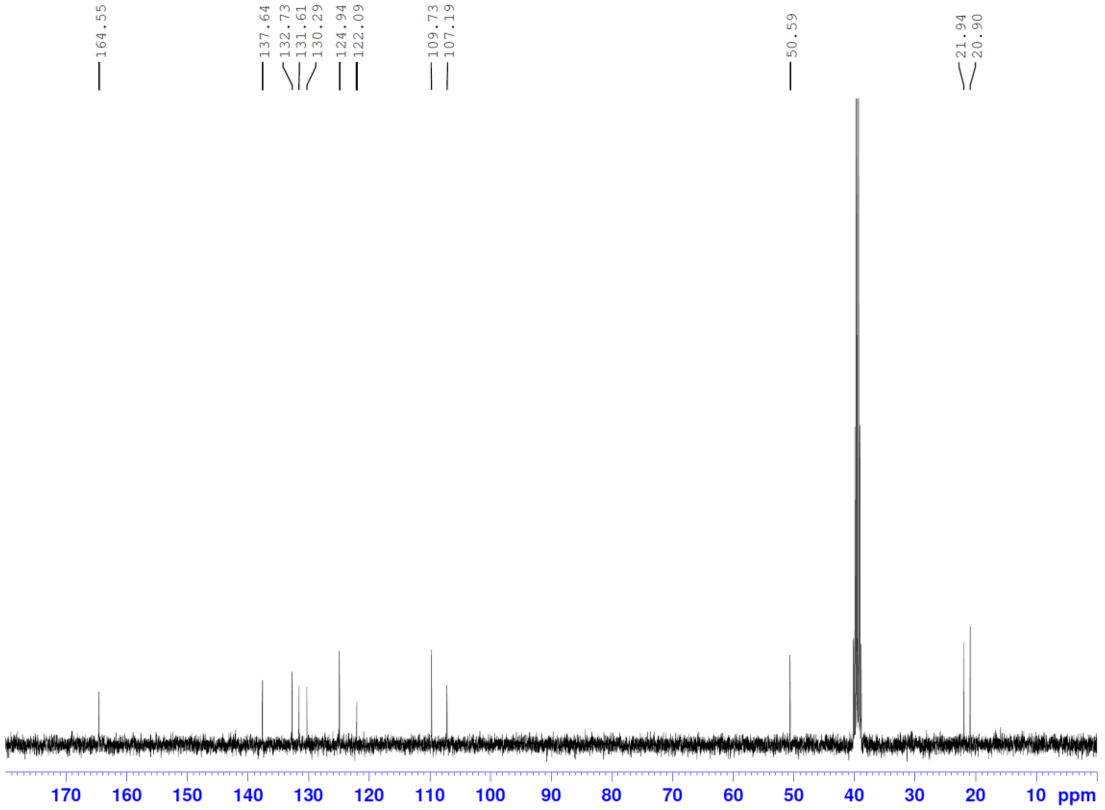
164.55
137.64
132.73
131.61
130.29
124.94
122.09
109.73
107.19
50.59
21.94
20.90
170
160
150
140
130
120
110
100
90
80
70
60
50
40
30
20
10
ppm

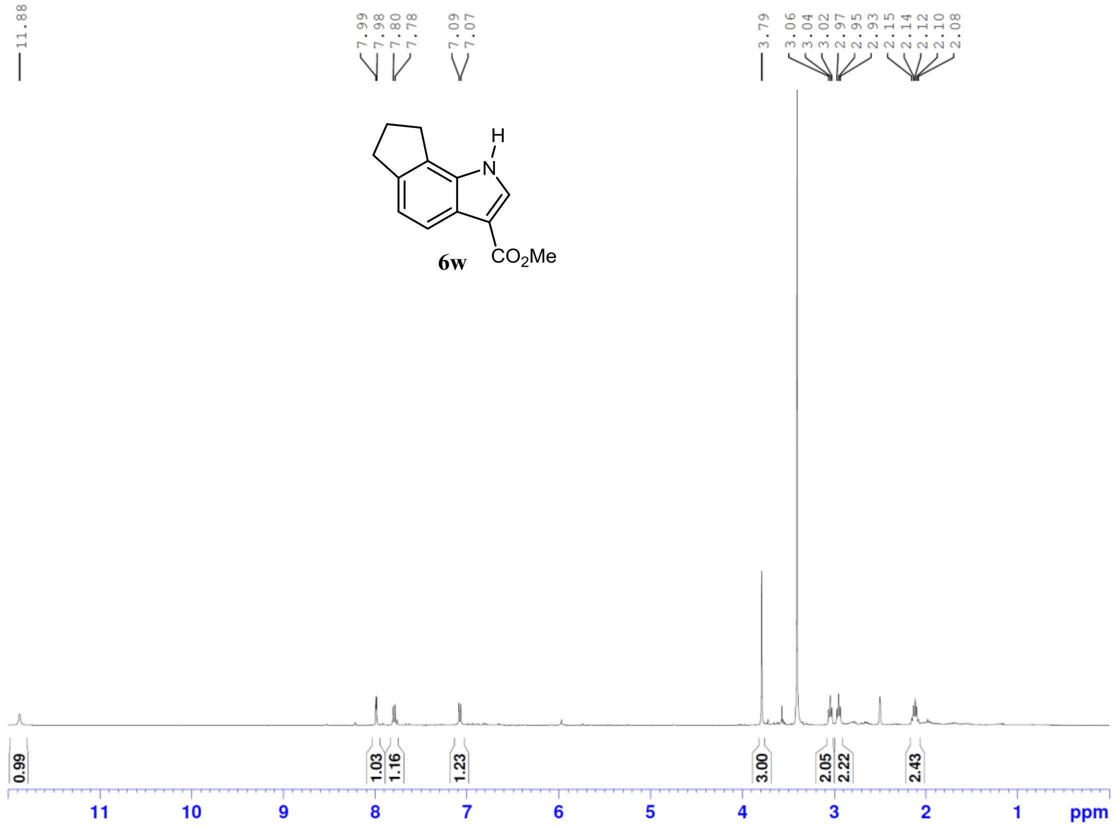
11.88
7.99
7.98
7.80
7.78
7.09
7.07
3.79
3.06
3.04
3.02
2.97
2.95
2.93
2.15
2.14
2.12
2.10
2.08
H
N
CO2Me
6w
0.99
1.03
1.16
1.23
3.00
2.05
2.22
2.43
11 10 9 8 7 6 5 4 3 2 1 ppm

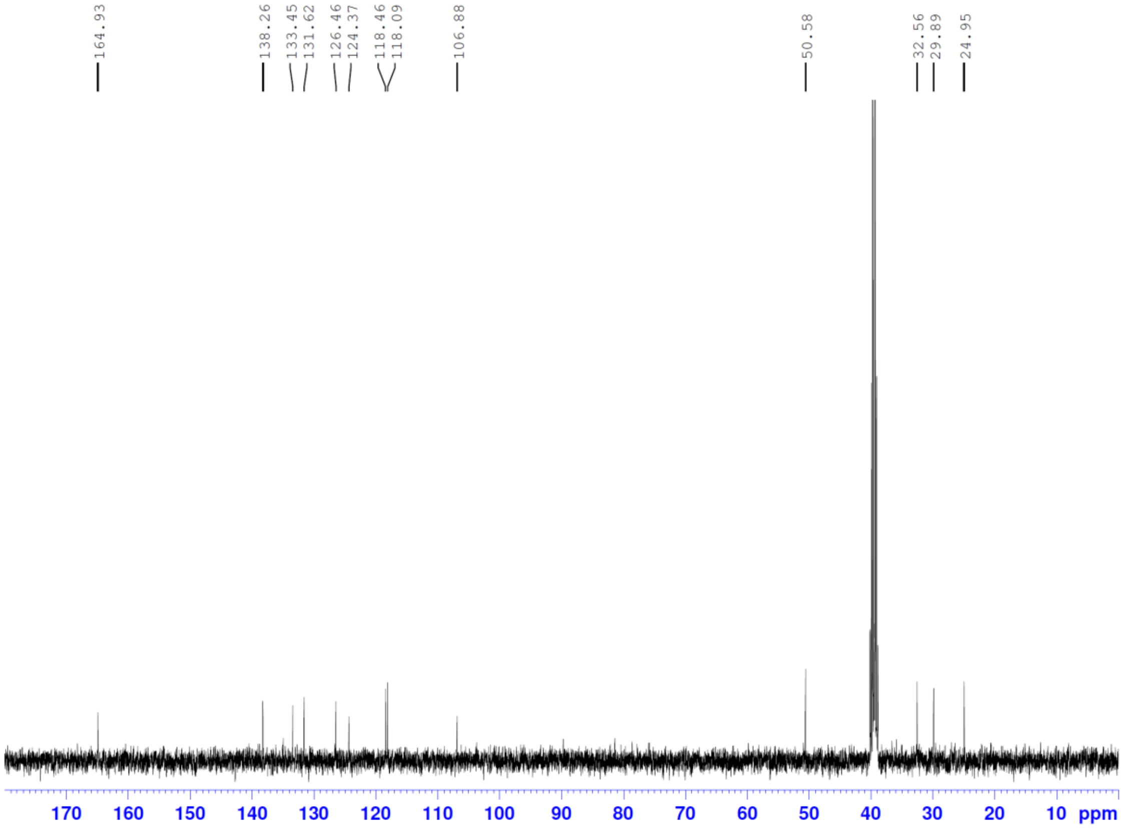
164.93
138.26
133.45
131.62
126.46
124.37
118.46
118.09
106.88
50.58
32.56
29.89
24.95
170 160 150 140 130 120 110 100 90 80 70 60 50 40 30 20 10 ppm

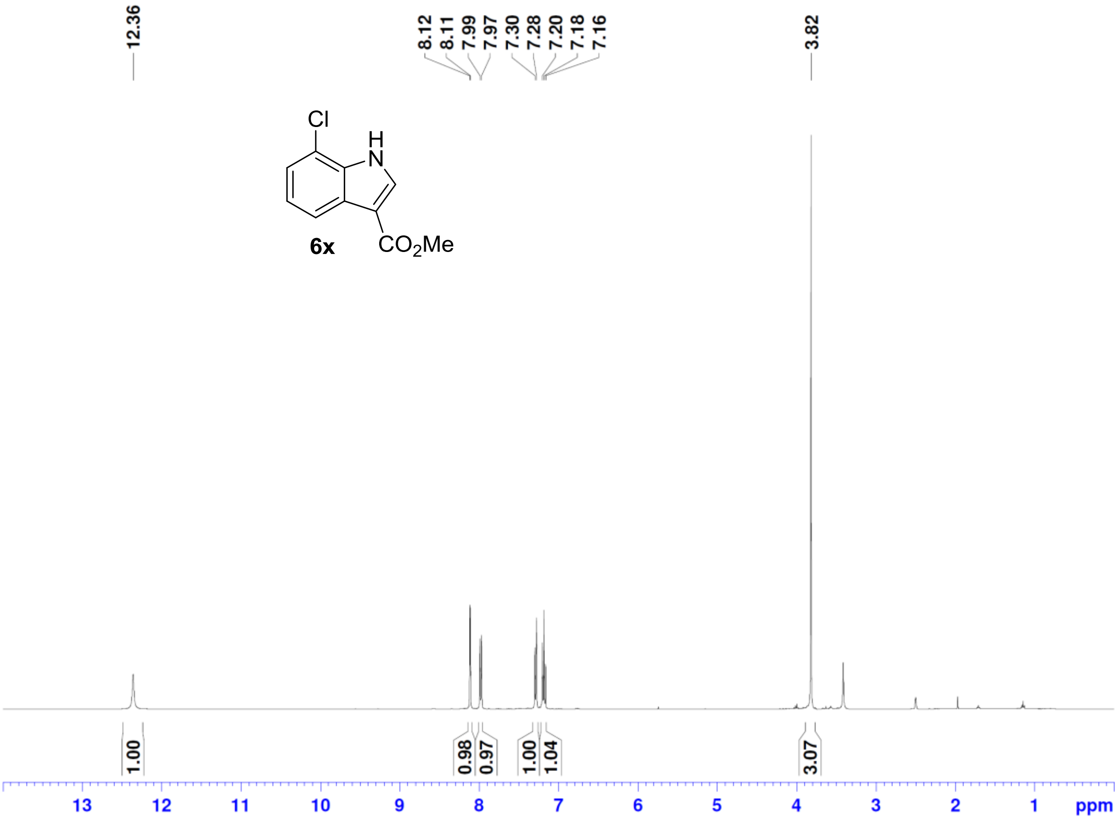

12.36
8.12
8.11
7.99
7.97
7.30
7.28
7.20
7.18
7.16
3.82
Cl
H
N
6x
CO2Me
1.00
0.98
0.97
1.00
1.04
3.07
13 12 11 10 9 8 7 6 5 4 3 2 1 ppm

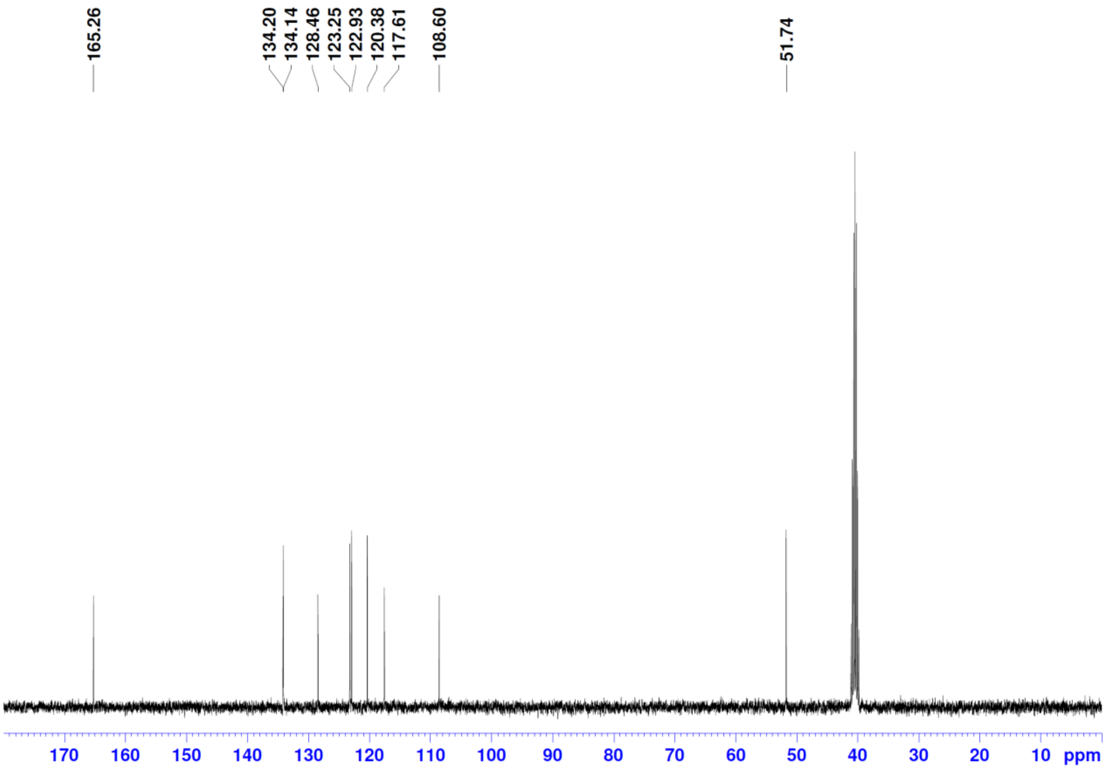

165.26
134.20
134.14
128.46
123.25
122.93
120.38
117.61
108.60
51.74
170 160 150 140 130 120 110 100 90 80 70 60 50 40 30 20 10 ppm

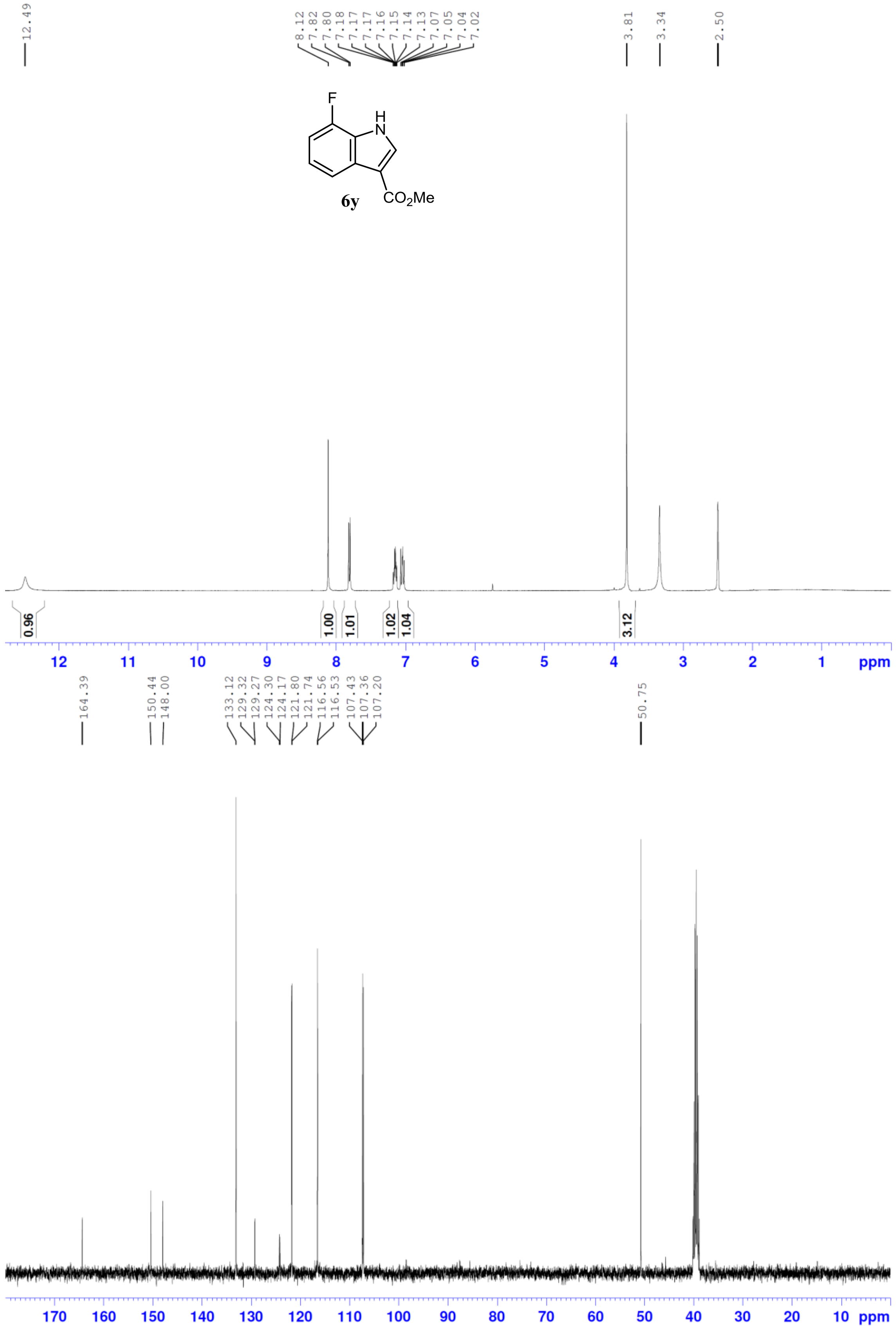
12.49
8.12
7.82
7.80
7.18
7.17
7.17
7.16
7.15
7.14
7.13
7.07
7.05
7.04
7.02
3.81
3.34
2.50
F
H
N
6y
CO2Me
0.96
1.00
1.01
1.02
1.04
3.12
12 11 10 9 8 7 6 5 4 3 2 1 ppm
164.39
150.44
148.00
133.12
129.32
129.27
124.30
124.17
121.80
121.74
116.56
116.53
107.43
107.36
107.20
50.75
170 160 150 140 130 120 110 100 90 80 70 60 50 40 30 20 10 ppm

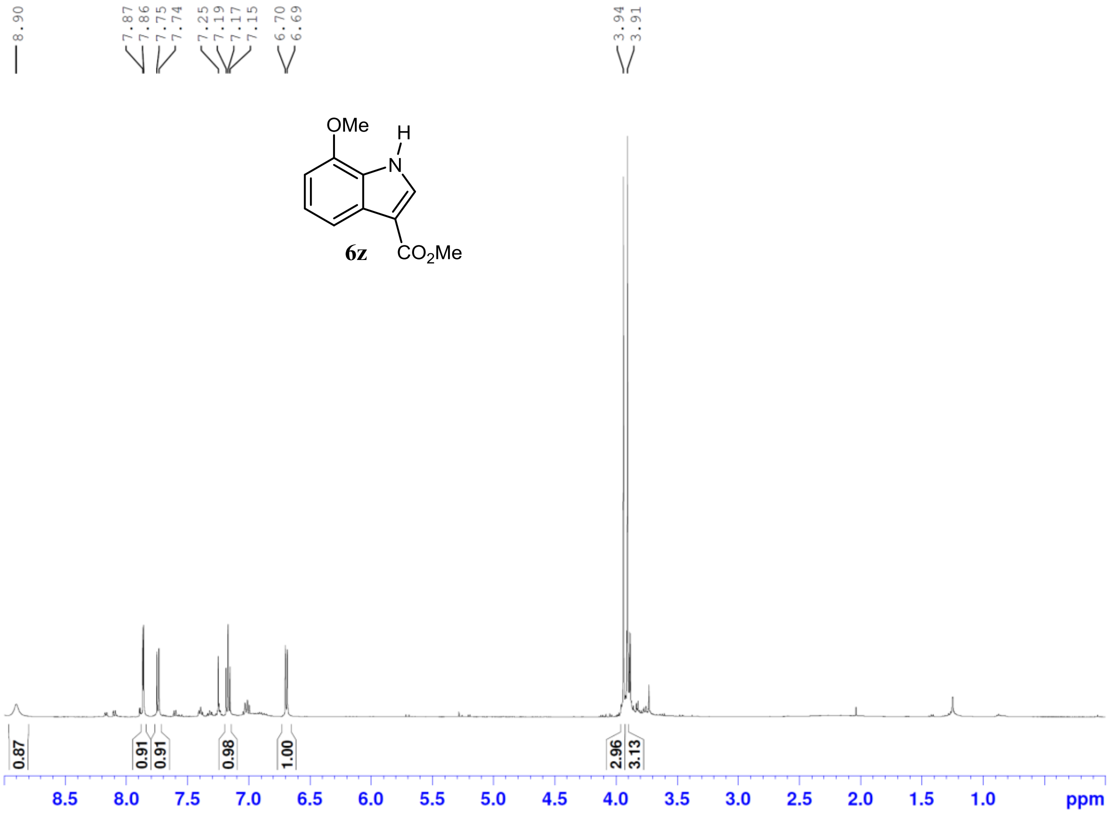
8.90
7.87
7.86
7.75
7.74
7.25
7.19
7.17
7.15
6.70
6.69
3.94
3.91
OMe
H
N
6z
CO_2Me
0.87
0.91
0.91
0.98
1.00
2.96
3.13
8.5 8.0 7.5 7.0 6.5 6.0 5.5 5.0 4.5 4.0 3.5 3.0 2.5 2.0 1.5 1.0 ppm

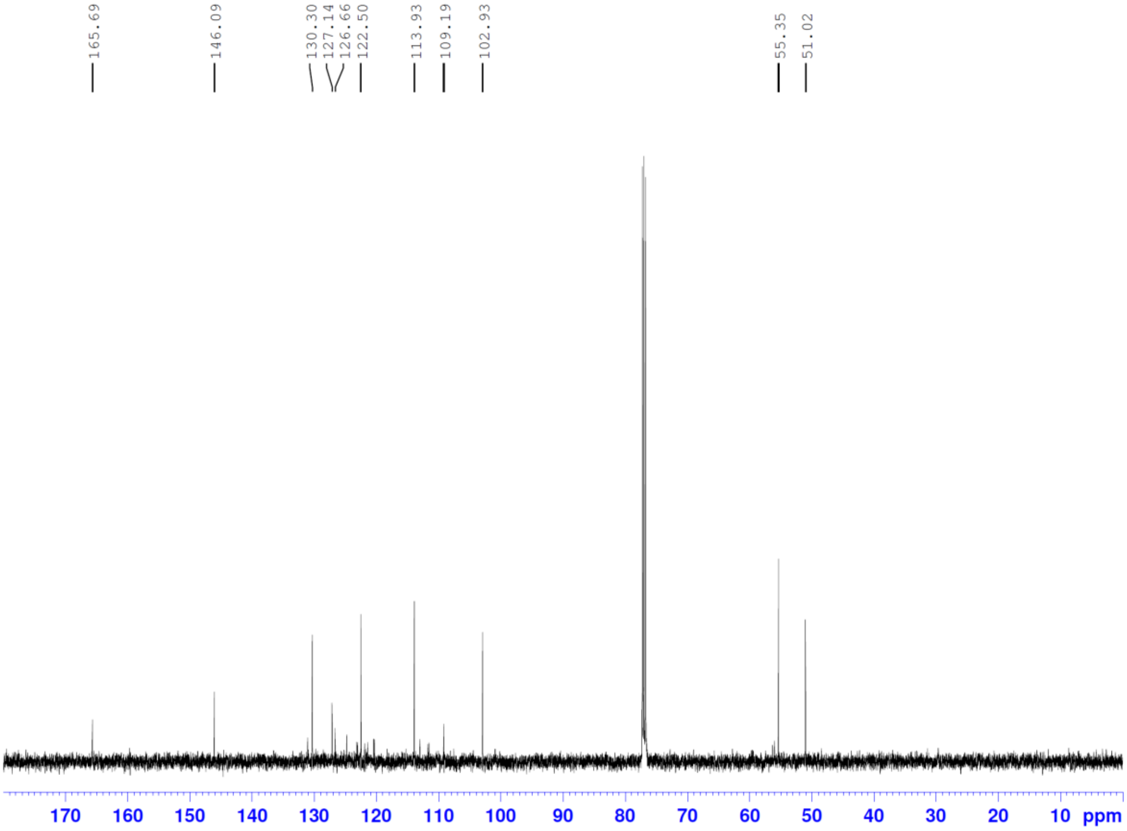
165.69
146.09
130.30
127.14
126.66
122.50
113.93
109.19
102.93
55.35
51.02
170 160 150 140 130 120 110 100 90 80 70 60 50 40 30 20 10 ppm

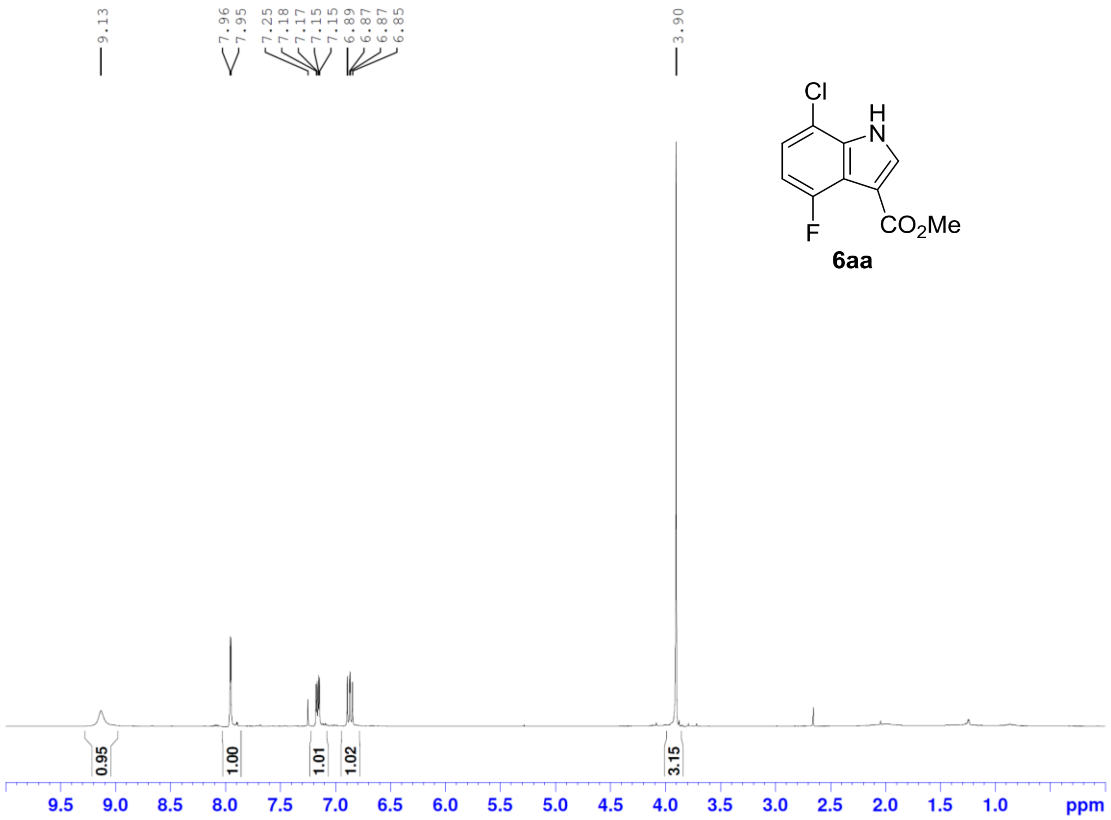
9.13
7.96
7.95
7.25
7.18
7.17
7.15
7.15
6.89
6.87
6.87
6.85
3.90
0.95
1.00
1.01
1.02
3.15
9.5 9.0 8.5 8.0 7.5 7.0 6.5 6.0 5.5 5.0 4.5 4.0 3.5 3.0 2.5 2.0 1.5 1.0 ppm
Cl
H
N
F
CO_2Me

6aa

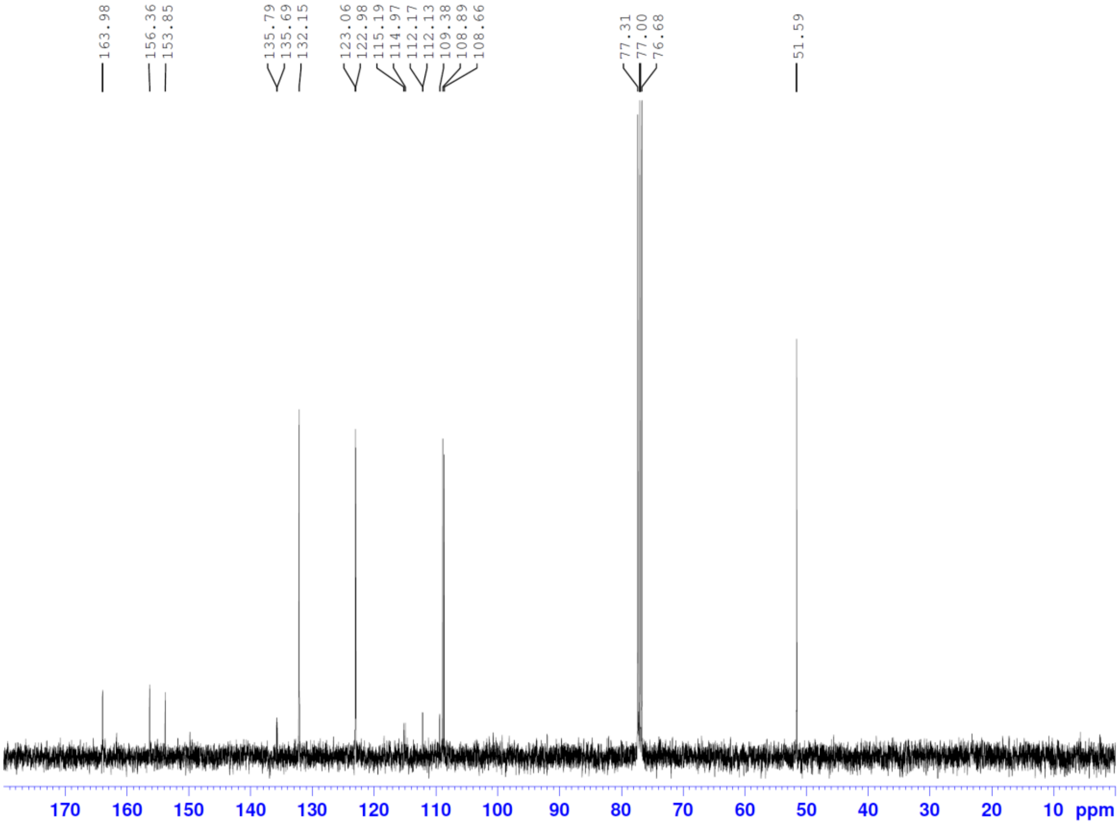
163.98
156.36
153.85
135.79
135.69
132.15
123.06
122.98
115.19
114.97
112.17
112.13
109.38
108.89
108.66
77.31
77.00
76.68
51.59
170 160 150 140 130 120 110 100 90 80 70 60 50 40 30 20 10 ppm

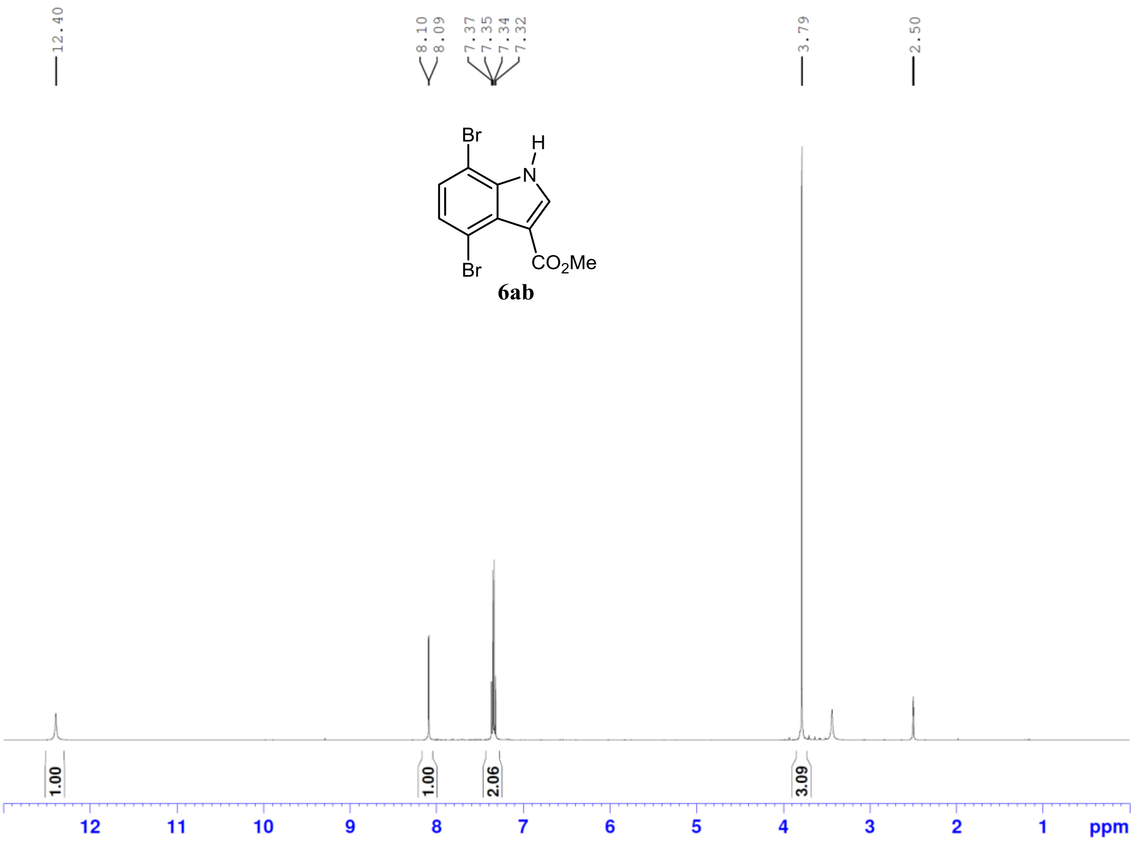
12.40
8.10
8.09
7.37
7.35
7.34
7.32
3.79
2.50
Br
H
N
Br
CO$_2$Me
6ab
1.00
1.00
2.06
3.09
12 11 10 9 8 7 6 5 4 3 2 1 ppm

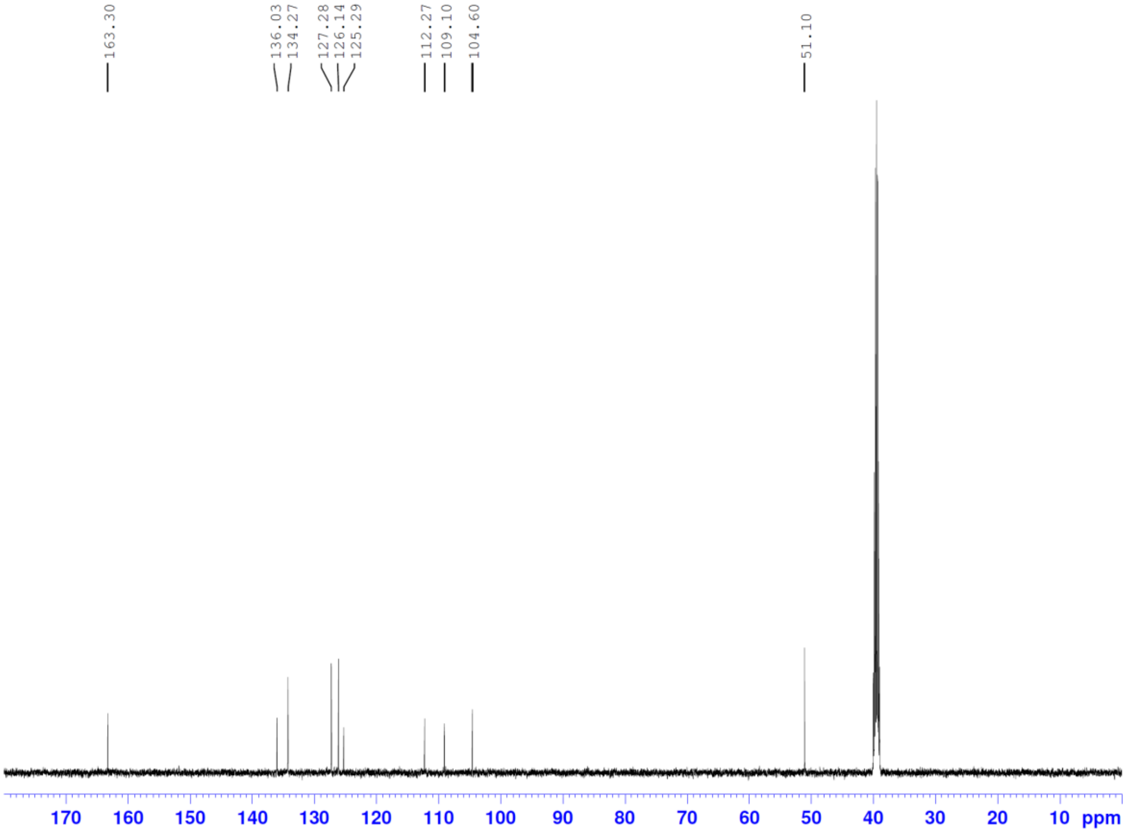
163.30
136.03
134.27
127.28
126.14
125.29
112.27
109.10
104.60
51.10
170 160 150 140 130 120 110 100 90 80 70 60 50 40 30 20 10 ppm

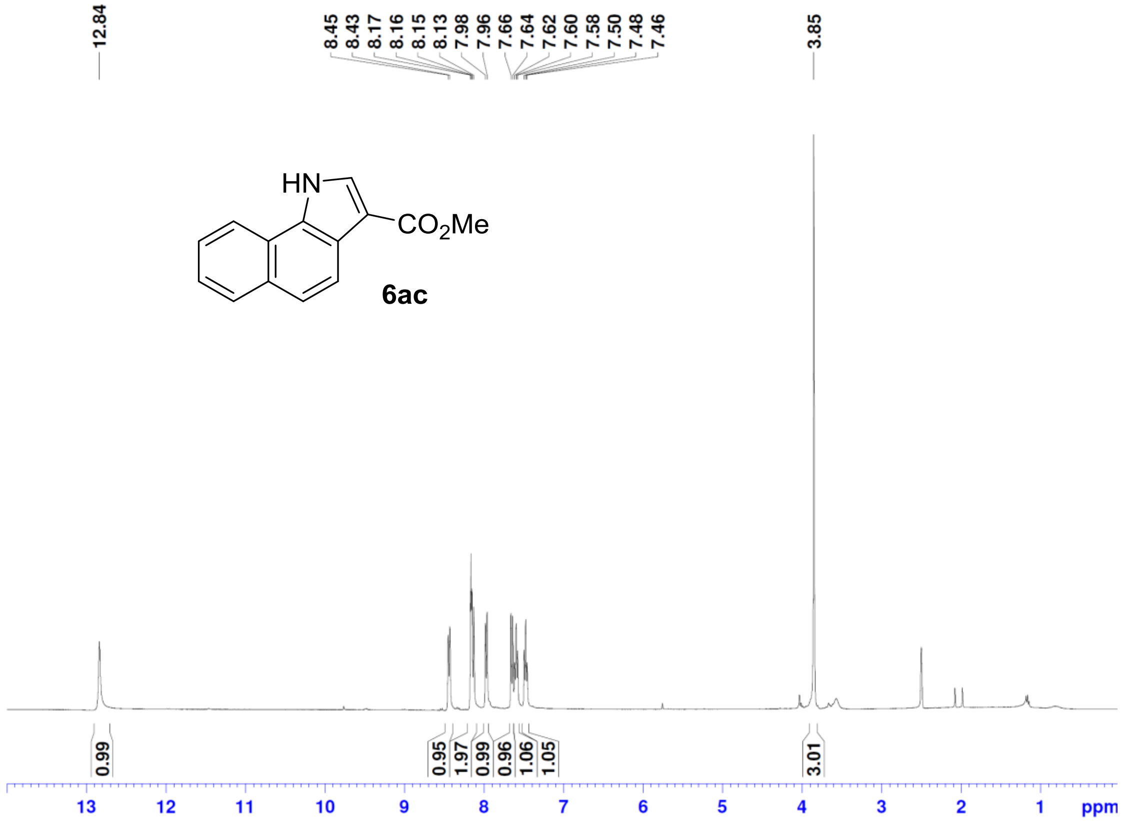
12.84
8.45
8.43
8.17
8.16
8.15
8.13
7.98
7.96
7.66
7.64
7.62
7.60
7.58
7.50
7.48
7.46
3.85
HN
CO2Me
6ac
0.99
0.95
1.97
0.99
0.96
1.06
1.05
3.01
13 12 11 10 9 8 7 6 5 4 3 2 1 ppm

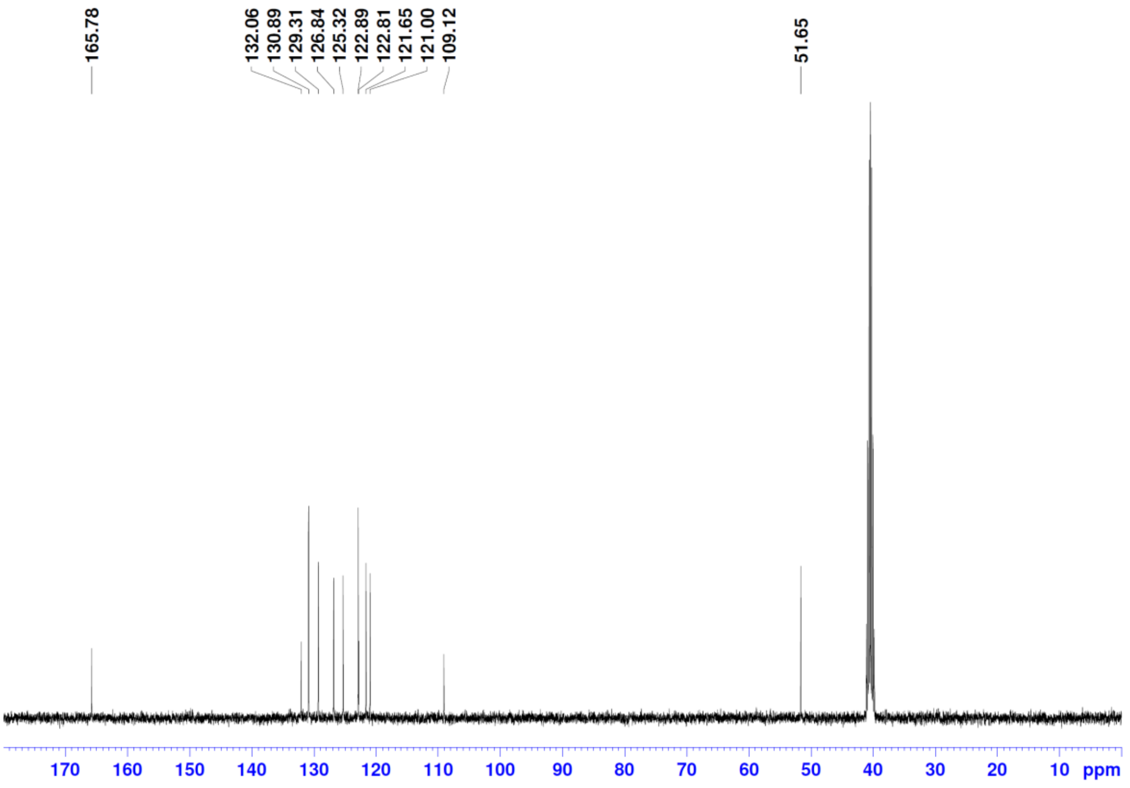
165.78
132.06
130.89
129.31
126.84
125.32
122.89
122.81
121.65
121.00
109.12
51.65
170 160 150 140 130 120 110 100 90 80 70 60 50 40 30 20 10 ppm

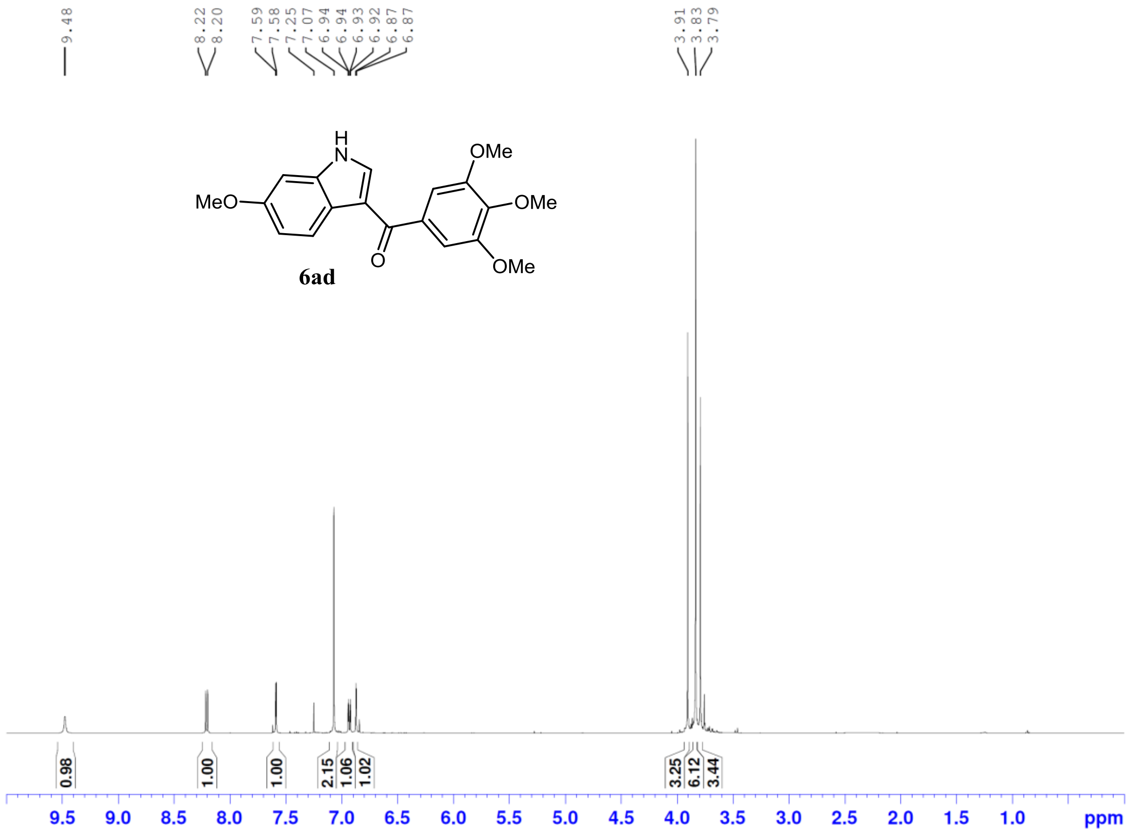
9.48
8.22
8.20
7.59
7.58
7.25
7.07
6.94
6.94
6.93
6.92
6.87
6.87
3.91
3.83
3.79
MeO
H
N
OMe
OMe
OMe
O
6ad
0.98
1.00
1.00
2.15
1.06
1.02
3.25
6.12
3.44
9.5 9.0 8.5 8.0 7.5 7.0 6.5 6.0 5.5 5.0 4.5 4.0 3.5 3.0 2.5 2.0 1.5 1.0 ppm

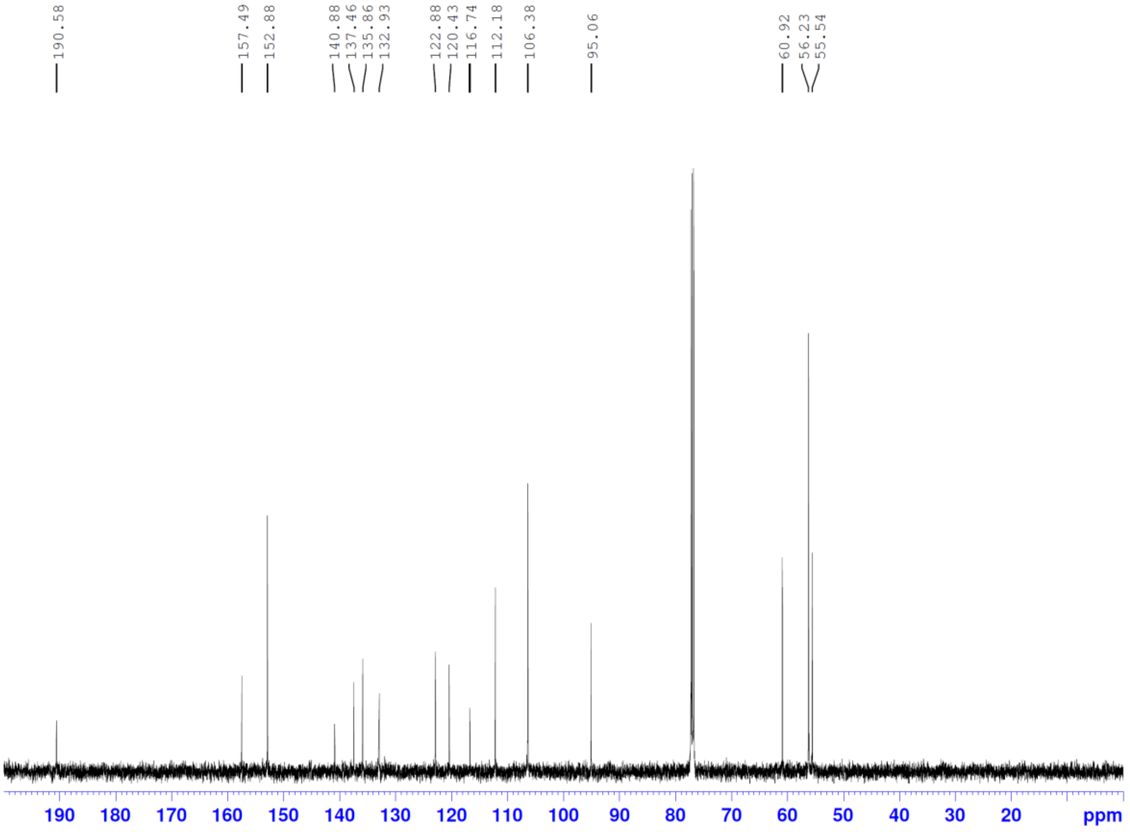
190.58
157.49
152.88
140.88
137.46
135.86
132.93
122.88
120.43
116.74
112.18
106.38
95.06
60.92
56.23
55.54
190 180 170 160 150 140 130 120 110 100 90 80 70 60 50 40 30 20 ppm

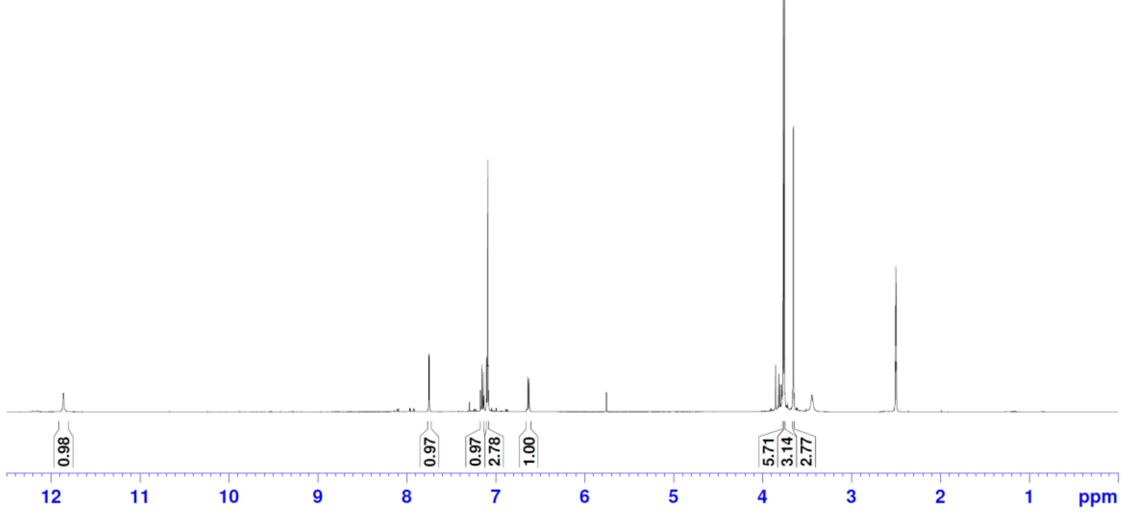

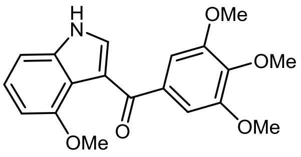

6ad (C-4 isomer)

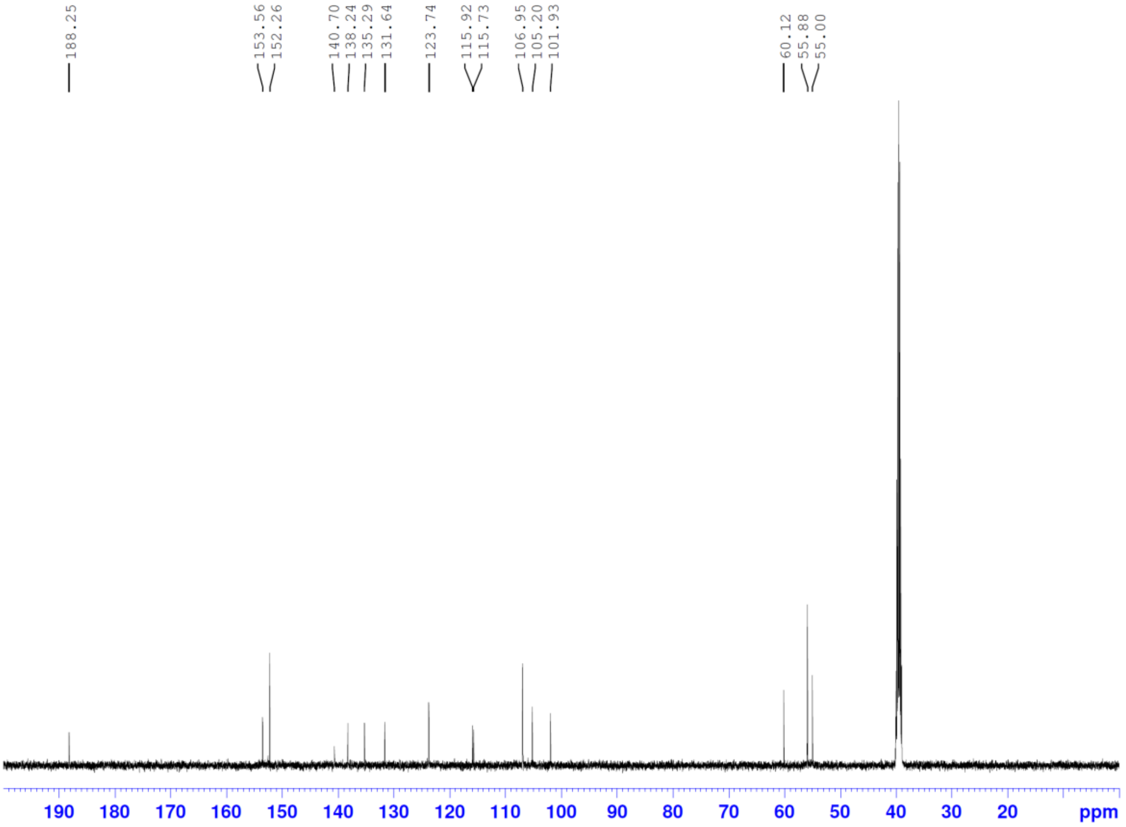

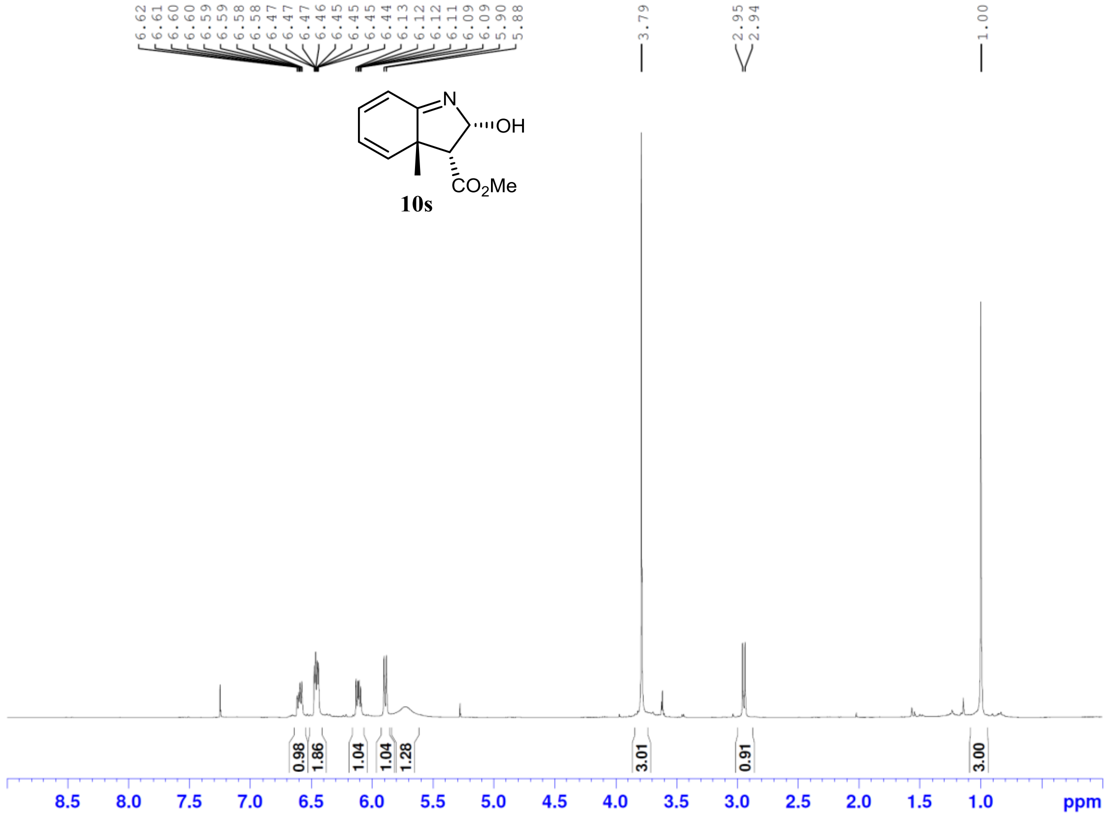
6.62 6.61 6.60 6.60 6.59 6.59 6.58 6.58 6.47 6.47 6.47 6.46 6.45 6.45 6.45 6.44 6.13 6.12 6.12 6.11 6.09 6.09 5.90 5.88
3.79
2.95 2.94
1.00
N
OH
CO2Me
10s
0.98 1.86 1.04 1.04 1.28 3.01 0.91 3.00
8.5 8.0 7.5 7.0 6.5 6.0 5.5 5.0 4.5 4.0 3.5 3.0 2.5 2.0 1.5 1.0 ppm

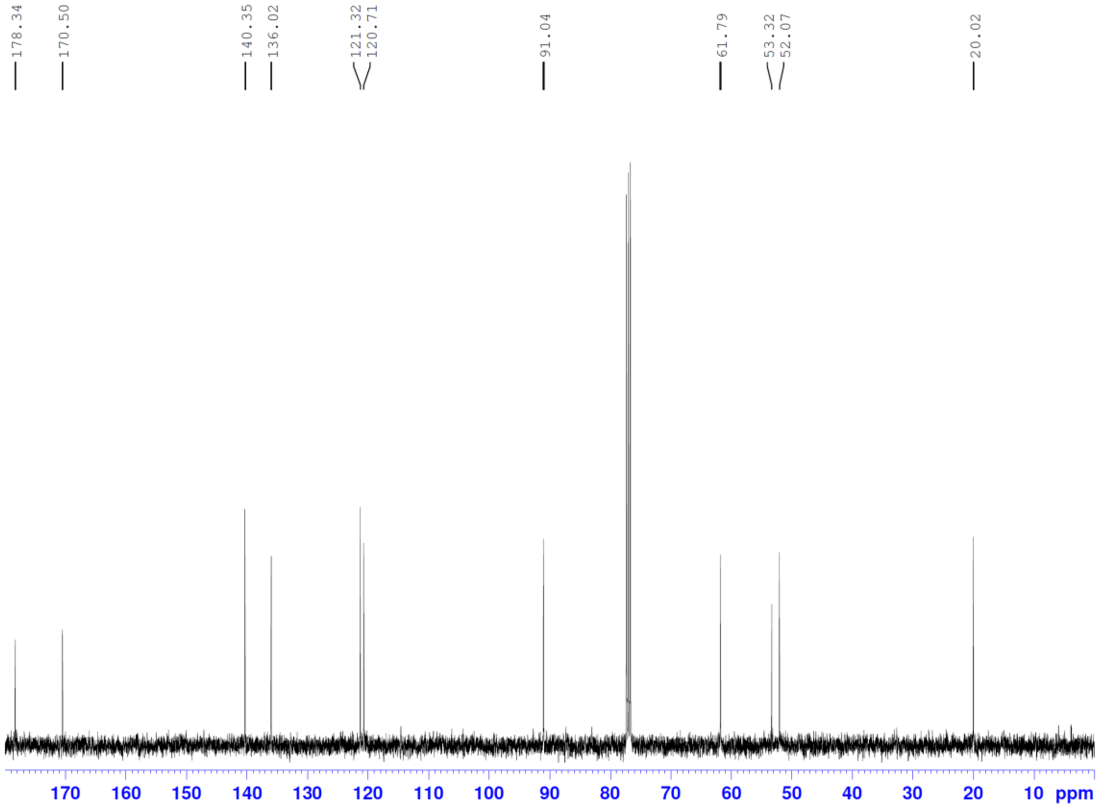
178.34 170.50 140.35 136.02 121.32 120.71 91.04 61.79 53.32 52.07 20.02
170 160 150 140 130 120 110 100 90 80 70 60 50 40 30 20 10 ppm

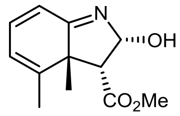
N
OH
CO_2Me

10t

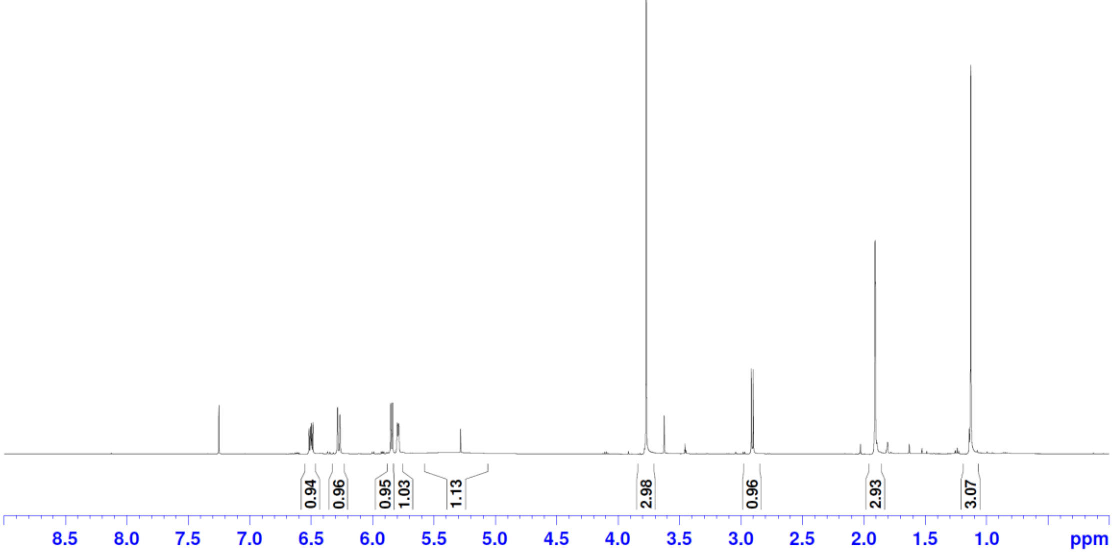
6.52
6.50
6.50
6.48
6.28
6.28
6.26
6.26
5.85
5.84
5.80
5.80
5.79
5.79
5.79
5.78
3.77
2.91
2.90
1.91
1.13
0.94
0.96
0.95
1.03
1.13
2.98
0.96
2.93
3.07
8.5
8.0
7.5
7.0
6.5
6.0
5.5
5.0
4.5
4.0
3.5
3.0
2.5
2.0
1.5
1.0
ppm

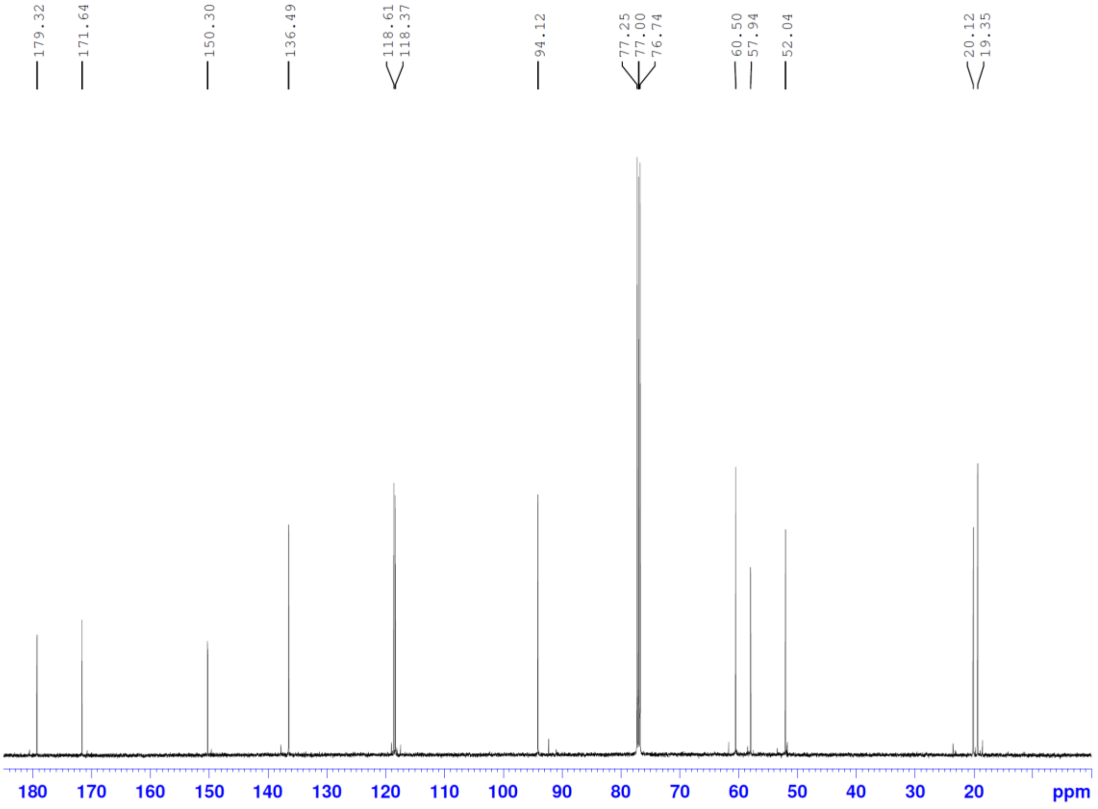
179.32
171.64
150.30
136.49
118.61
118.37
94.12
77.25
77.00
76.74
60.50
57.94
52.04
20.12
19.35
180
170
160
150
140
130
120
110
100
90
80
70
60
50
40
30
20
ppm

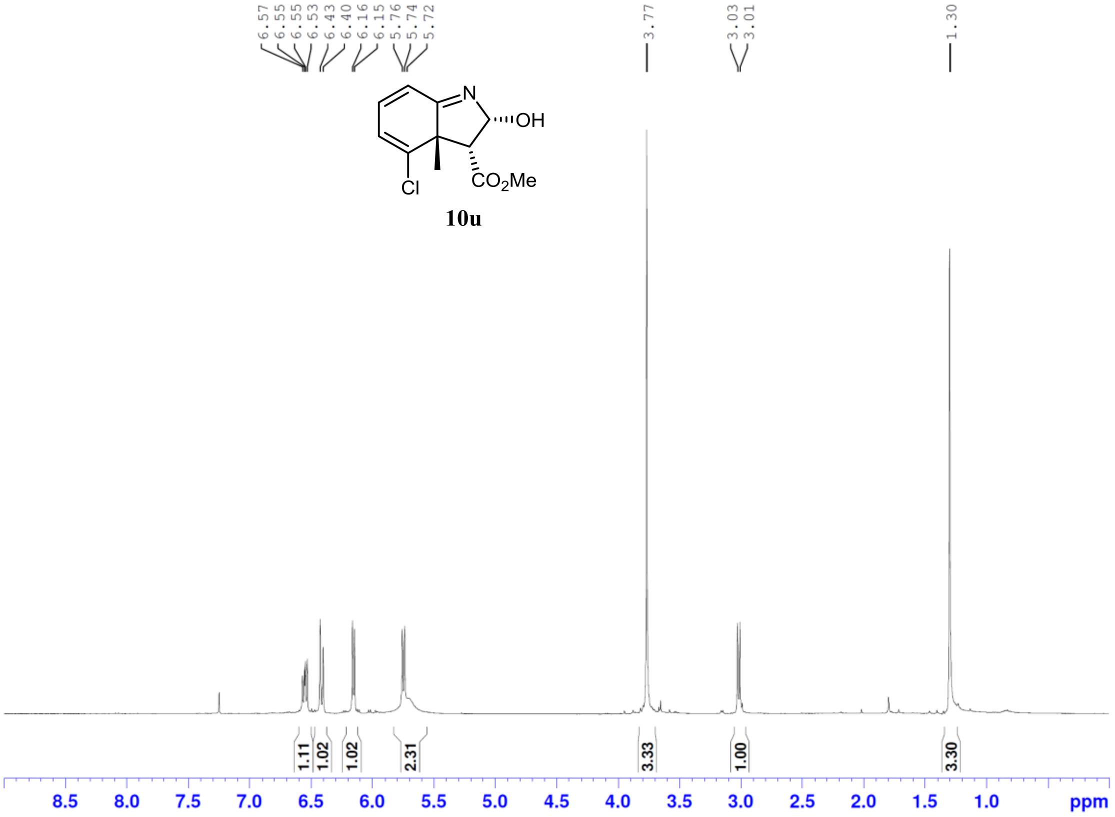
6.57
6.55
6.55
6.53
6.43
6.40
6.16
6.15
5.76
5.74
5.72
3.77
3.03
3.01
1.30
N
OH
Cl
CO_2Me
1.11
1.02
1.02
2.31
3.33
1.00
3.30
8.5 8.0 7.5 7.0 6.5 6.0 5.5 5.0 4.5 4.0 3.5 3.0 2.5 2.0 1.5 1.0 ppm

10u

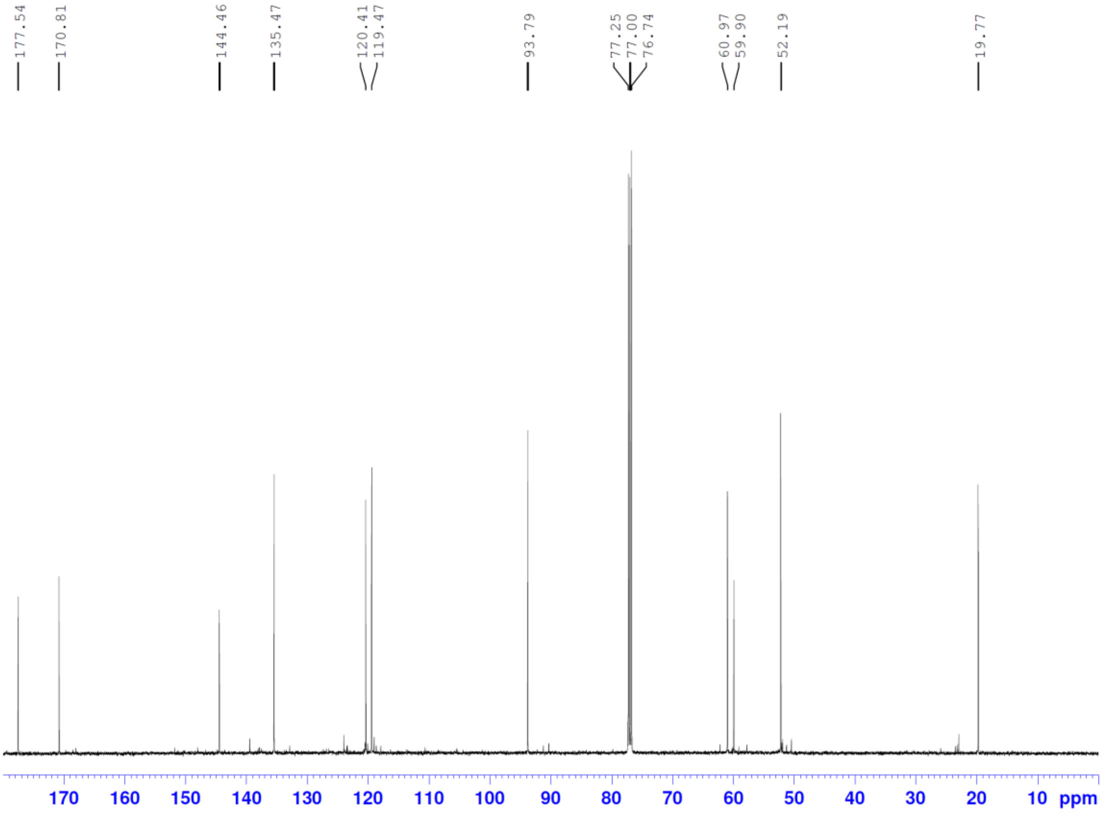
177.54
170.81
144.46
135.47
120.41
119.47
93.79
77.25
77.00
76.74
60.97
59.90
52.19
19.77
170 160 150 140 130 120 110 100 90 80 70 60 50 40 30 20 10 ppm

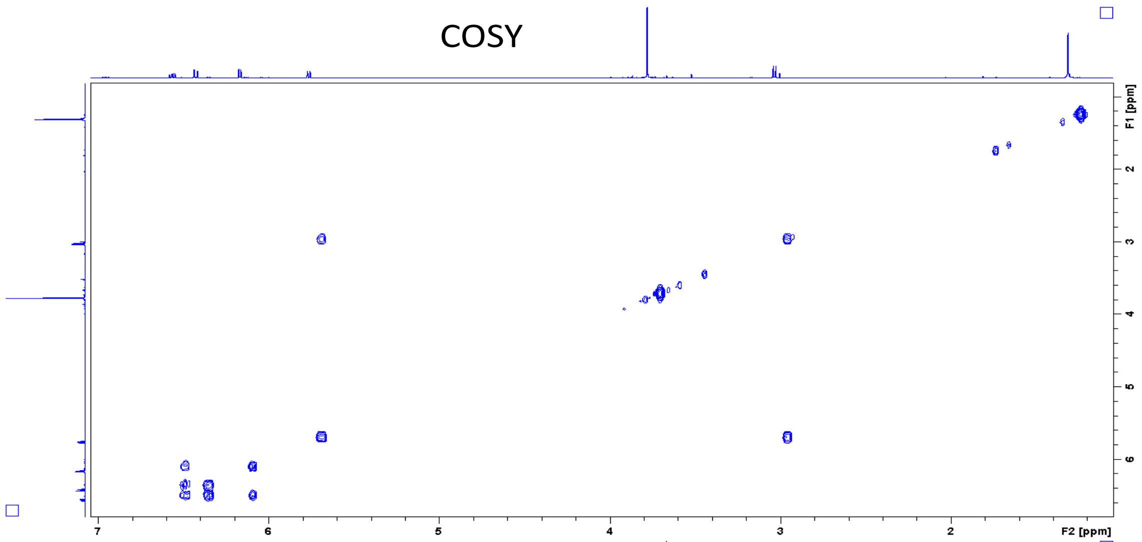
COSY
F1 [ppm]
2
3
4
5
6
7
6
5
4
3
2
F2 [ppm]

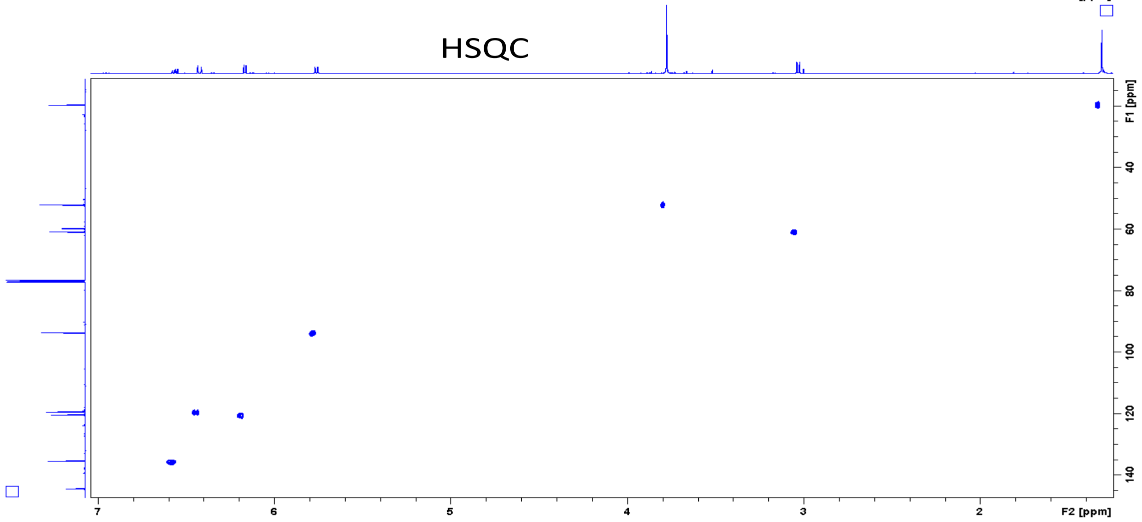
HSQC
F1 [ppm]
40
60
80
100
120
140
7
6
5
4
3
2
F2 [ppm]

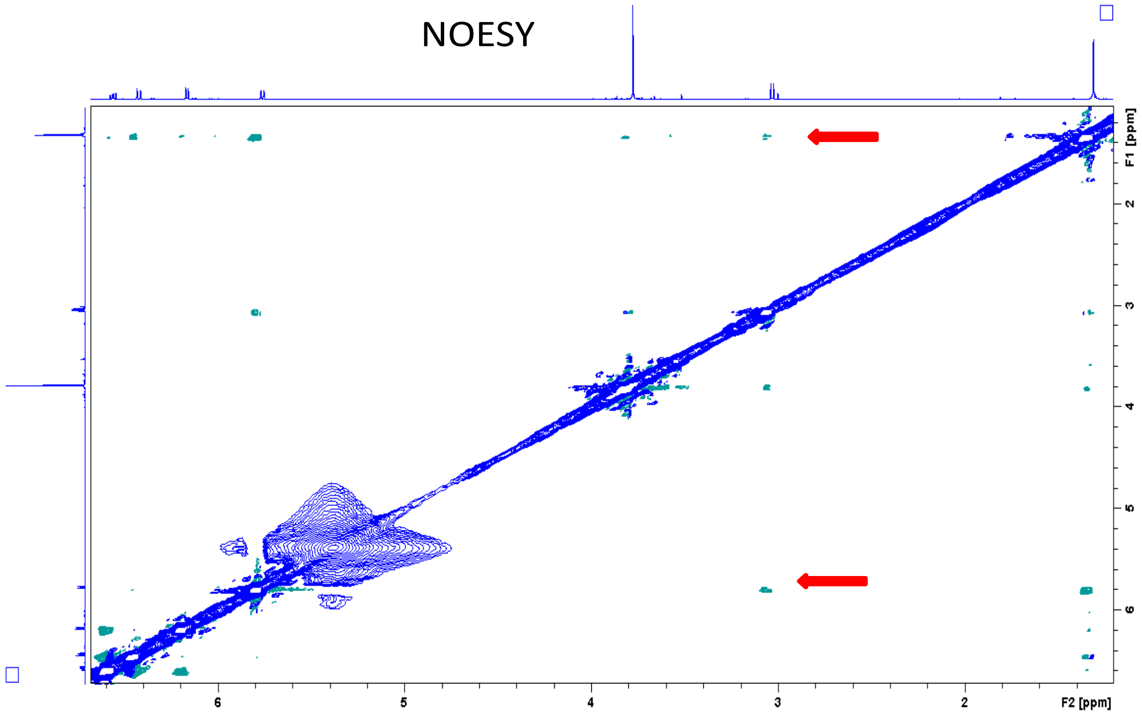
NOESY
F1 [ppm]
2
3
4
5
6
6
5
4
3
2
F2 [ppm]

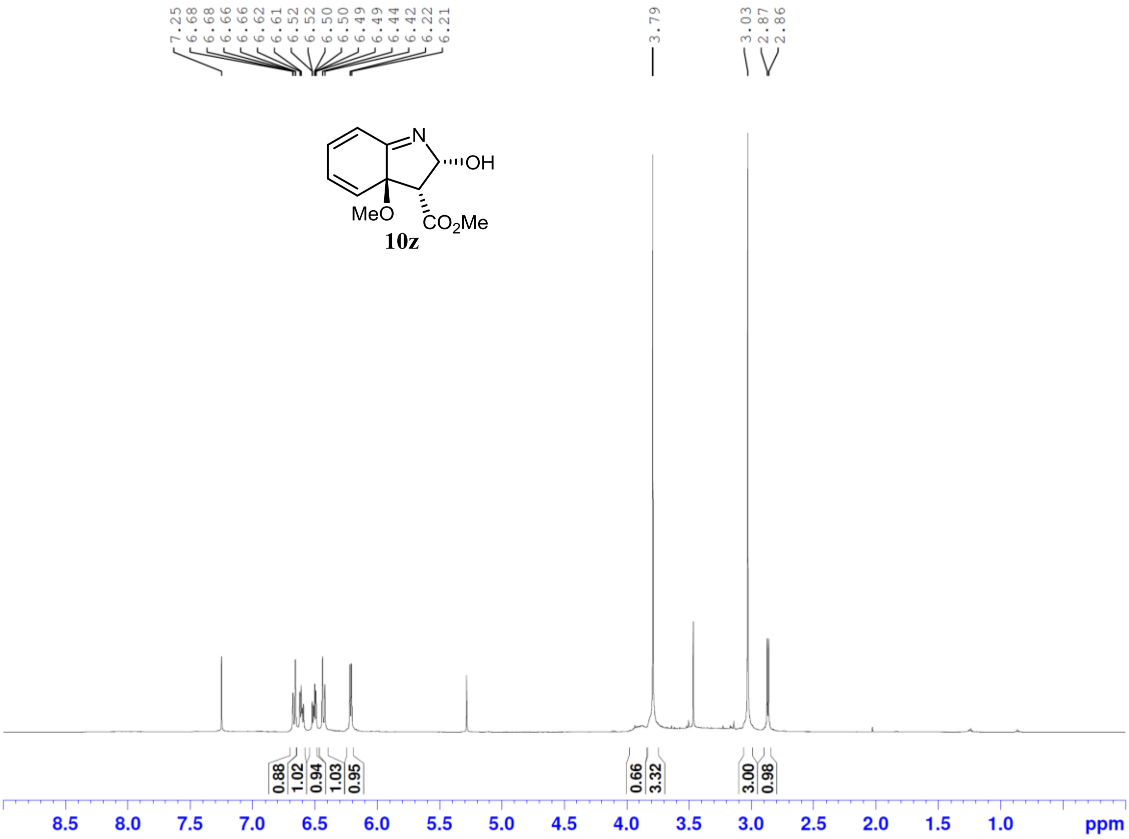
7.25
6.68
6.68
6.66
6.66
6.62
6.61
6.52
6.52
6.50
6.50
6.49
6.49
6.44
6.42
6.22
6.21
3.79
3.03
2.87
2.86
N
OH
MeO
CO_2Me
10z
0.88
1.02
0.94
1.03
0.95
0.66
3.32
3.00
0.98
8.5 8.0 7.5 7.0 6.5 6.0 5.5 5.0 4.5 4.0 3.5 3.0 2.5 2.0 1.5 1.0 ppm

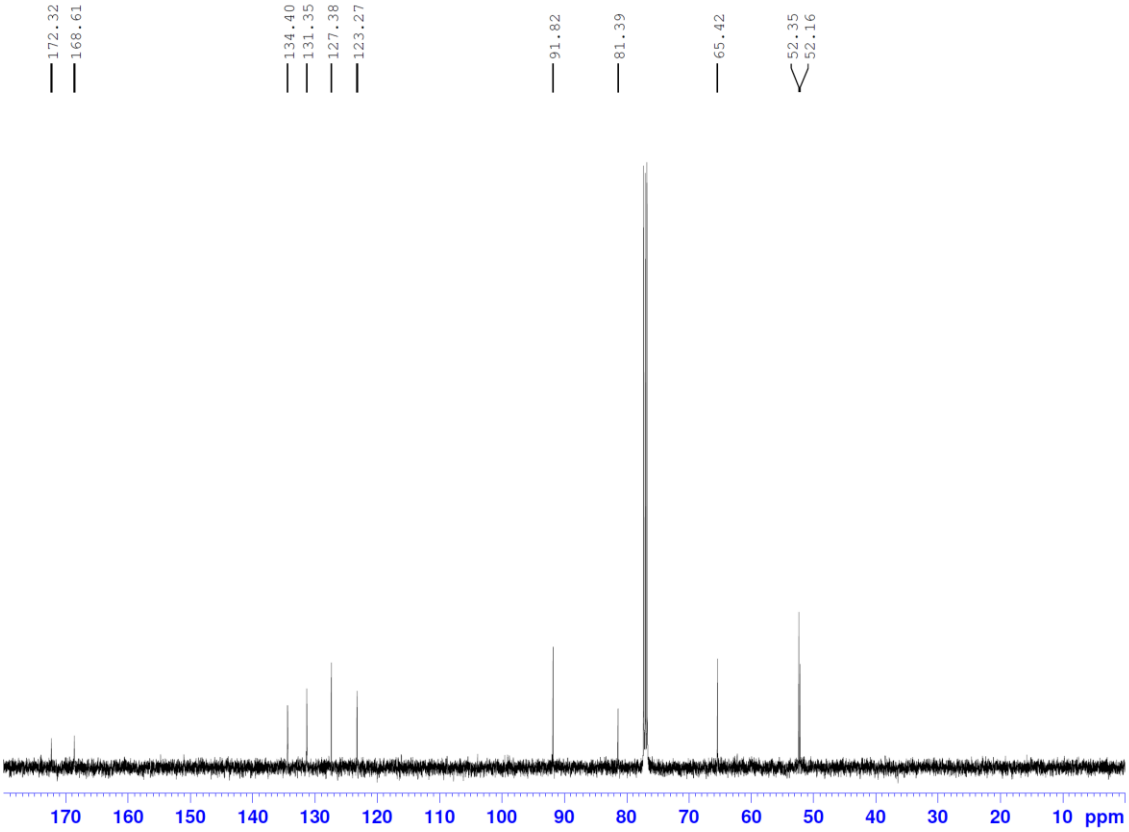
172.32
168.61
134.40
131.35
127.38
123.27
91.82
81.39
65.42
52.35
52.16
170 160 150 140 130 120 110 100 90 80 70 60 50 40 30 20 10 ppm
